# Supplementary material for: Sub-micro droplet reactors for green synthesis of Li3VO4 anode materials in lithium ion batteries
Source: Nat Commun. 2021 May 25;12:3081. doi: 10.1038/s41467-021-23366-8 (PMC8149873; doi:10.1038/s41467-021-23366-8)
Supplement: Supplementary file 1 — Supplementary Information [file 41467_2021_23366_MOESM1_ESM.docx]

**Supplementary Information**

**Sub-micro droplet reactors for green synthesis of Li_3_VO_4_ anode materials in lithium ion batteries**

Ha Tran Huu^1^, Ngoc Hung Vu^2,3^, Hyunwoo Ha^4^, Joonhee Moon^5^, Hyun You Kim^4^_,_

and Won Bin Im^1,*^

*^1^Division of Materials Science and Engineering, Hanyang University, 222, Wangsimni-ro, Seongdong-gu, Seoul, 04763, Republic of Korea*

*^2^Falcuty of Biotechnology, Chemistry and Environmental Engineering, Phenikaa University, Hanoi 10000, Vietnam*

*^3^Phenikaa Research and Technology Institute, A&A Green Phoenix Group, 167 Hoang Ngan, Hanoi 10000, Vietnam*

*^4^* *Department of Materials Science and Engineering, Chungnam National, University, Daejeon, 34134, Korea*

*^5^Advanced Nano-Surface Research Group, Korea Basic Science Institute, Daejeon 34133, Republic of Korea*

^*^ To whom correspondence should be addressed.

Tel : +82-2-2220-0404

E-mail : imwonbin@hanyang.ac.kr

**Supplementary Methods**

*Calculation of water droplet size*

In order to investigate the size-controlling function of steam droplet, an application of Koehler theory^1, 2^ was conducted. In details, in the initial stage, the reaction only begins when water evaporates from liquid form to molecular vapor. Following the Antoine equation^3^, the vapor pressure of water at 80^o^C was estimated while the saturated value was derived from application of Clapeyron – Clausius equation. Accordingly, the approximate saturation degree, S, of synthesis system was only 0.872 illustrating that the environment inside the vial reactor is sub-saturated condition and not sufficient for the homogeneous or spontaneous nucleation of small water cluster to form further droplet condensation,^4^ which could be clarified *via* Kelvin or curvature effect.^5^ However, growth of vapor droplet could be accelerated by heterogeneous nucleation based on the Raoult or solution effect in which the presence of soluble molecular reduces evaporation rate and hastens the formation as well as survival of droplet. Following the Koehler theory, a combination of two above effects, which could be briefly presented by the Supplementary Equation 1, the droplet growth *via* condensation and coalescence with surrounding water molecules is determine by the critical saturation degree, S_c_:

$S=1+\frac{a}{r}-\frac{b}{r^{3}}$ (1)

where,

*S*: saturation degree of surrounding environment

$$a=\frac{2\sigma}{n_{L}RT}$$

$$b=\frac{3iN_{S}}{4\pi n{}_{L}}$$

$\text{σ}$: surface tension of water, at 80^o^C, 6.26$\times$10^-2^ (N/m^2^)

$\text{n}_{\text{L}}$: molar volume of water

$\text{R}$: ideal gas constant

$\text{i}$: Van’t Hoff factor presenting the number of components split from solute when it is dissolved

$\text{N}_{\text{s}}$: total number of moles of solute, typically in range of 10^-18^ to 10^-15^ moles

and,

$\text{S}_{\text{c}}\text{=}\sqrt{\frac{\text{4}\text{a}^{\text{3}}}{\text{27b}}}\text{+1}$ > S_system_

*Method for estimation of saturation degree, S*

*Estimate the vapor pressure, e, by application of Antoine Equation:*

Vapor Pressure of Water calculator Formula:

P = 10^A-B/(C+T)^ (2)
Where:

*P*: Vapor Pressure of Water

*T*: Water Temperature, in Celsius

*A*, *B*, *C*: Antoine Constants for Water.

When water temperature in the range of 1 – 100^o^C:

A = 8.07131, B = 1730.63, C = 233.426;

When the temperature in the range of 99 – 374^o^C:

A = 8.14019, B = 1810.94, C = 244.485.

At 80^o^C, P = e = 354.5323 mmHg

*Estimate the saturation vapor pressure, e_S_, by using Clapeyron-Clausius Equation:*

ln$\left( \frac{\text{e}_{\text{sT1}}}{\text{e}_{\text{sT2}}} \right)$=$\frac{\text{∆}\text{H}_{\text{evaporation}}}{\text{R}}\left( \frac{\text{1}}{\text{T2}}\text{-}\frac{\text{1}}{\text{T1}} \right)$ (3)

*e_sT_*: saturated vapor pressure at T (K)

*R*: Ideal gas constant = 8.314 J⸳mol^-1^⸳K^-1^

$\Delta H_{evaporation}:$Latent heat of water evaporation = 40650 J⸳mol^-1^

At T_1_ = 273K, e_s1_ = 4.58 mmHg

$\to$ At T_2_ = 353K, e_s2_ = 406.573 mmHg

$\boldsymbol{\to}$ **S = e/e_S_ = 0.872.**

*Lithium ion diffusion coefficient determination method*

The lithium ion diffusion coefficient ($\text{D}_{\text{Li}^{\text{+}}}$) was also estimated using the following equation:

$D_{Li^{+}}=\frac{R^{2}T^{2}}{2A^{2}n^{4}F^{4}C^{2}\sigma^{2}}$ (4)

in which, *R* = 8.314 J⸳mol^-1^⸳K^-1^ is the ideal gas constant, *T* = 298.15 K is room temperature in Kelvin scale, *A* is the effective surface area of the electrode (= specific surface area $\times$ mass of active material), *n* = 1 is number of exchange electrons in the redox process, *F* = 96485 C⸳mol^-1^ is Faraday constant, *C* (mol⸳cm^-3^) is concentration of lithium ions (as concentration of LiPF_6_ in electrolyte of 1 mol⸳dm^-3^ = 1000 mol⸳cm^-3^) and *σ* (Ω⸳s^-0.5^) is the Warburg coefficient that is deduced as the slope from linear relationship between the real impedance *Z’* and the angular frequency *ω^-0.5^* using Supplementary Equation (5) as shown in Supplementary Figure 13.

Z’ = R_e_ +R_ct_+ σ$\times$ω^-0.5^ (5)

*Pseudocapacitance contribution calculation*

Fundamentally, the nano-sized materials could store lithium ions in several following pathways: i) electrical double-layer capacitance (EDLC), referring to the charge separation in a Helmholtz double layer at the electrode/electrolyte interface; ii) pseudocapacitive process, which is the charge transfer process primarily occurring at the electrode/electrolyte interface according to three faradaic mechanisms, underpotential deposition, redox pseudocapacitance, and intercalation pseudocapacitance; and iii) diffusion-controlled redox process, in which the kinetics mostly are limited by the slow rate of the solid phase transition.^6, 7, 8, 9, 10^

To calculate the pseudocapacitive charge contribution to the total charge storage of the 36LCVO-ABR, the 3LCVO-ABR electrode was tested CV at different scan rates from 0.1 to 5 mV⸳s^-1^ between 0.2 and 3.0 V vs. Li^+^/Li. Based on the concept of power-law and cumulative principle as below:^11^

i = *a*ν*^b^* (6)

where, i represents the obtained reduction peak current (mA) corresponding to a certain scan rate, ν (mV⸳s^-1^); *a* and *b* are constants. The *b*-value, an indicative for each charge storage process, could be calculated from linear plot of log(i) *vs.* log(ν) as shown in Figure 5d. In detail, the intercalation – alloying effect was determined by the diffusion-controlled Supplementary Equation S7:

i = nFAC^*^D^1/2^ν^1/2^(αnF/RT)^1/2^π^1/2^χ(bt) (7)

in which, *n* is number of transferring electrons, *F* is Faraday constant, *A* is effective contact area between electrode and electrolyte, *C^*^* is the surface concentration of electrode materials, *D* is chemical diffusion coefficient, *α* is the transfer coefficient, *R* is the ideal gas constant, *T* is the Kelvin temperature and *χ(bt)* is the normalized current function.^11^ According to Supplementary Equation (7), the *b*-value of the diffusion-controlled process should be assigned as closed to 0.5. On the other hand, the capacitive effect could be assigned via linear or near-linear relationship between capacitive current and scan rate:^11^

i = C_d_Aν (8)

where C_d_ corresponds to capacitance. Consequently, *b*-value of capacitive effect is determined as 1.0 for EDLC and $\sim$1.0 for pseudocapacitive process.^12^

For further analysis, to study the contribution of each process in total capacity, the cumulative principle was applied based on the concept of separating response current into two components:^13^

i = k_1_ν + k_2_ν^1/2^ (9)

or the mathematic-transferring form:

iν^-1/2^ = k_1_ν^1/2^ + k_2_ (10)

Where, k_1_ is related to capacitive effect and k_2_ corresponds to diffusion-controlled constant.

According to the Supplementary Equations (8, 9), the linear plot of i/ν^1/2^ vs. ν^1/2^ was established at several collected potentials (Supplementary Figure 15a). The k_1_ and k_2_ were calculated to be contribution constant of, capacitance and diffusion current, respectively. In detail, based on the slopes calculated via linear plots of Supplementary Equation 10 collecting from selected potential points (e.g, Supplementary Figure 15a), individual respective k_1_ and k_2_ were obtained. Then, a pseudocapacitance response current i_p_ = k_1_ν was plotted in each certain scan rate as shown in Supplementary Figure 15b-f. By using mathematic integral method, the contribution of i_p_ in total charge was estimated via:

$\text{\%pseudo-capacitance = }\frac{\text{S}_{\text{i}_{\text{p}}}}{\text{S}_{\text{i}}}\text{ ×}\text{100\%}\text{ }$ (11)

in which, $\text{S}_{\text{i}_{\text{p}}}$ presents for area portion under i_p_ curves corresponding to contribution of pseudocapacitance while $\text{S}_{\text{i}}$ exhibits for area potion under original curve related to total charge storage capacity.

**
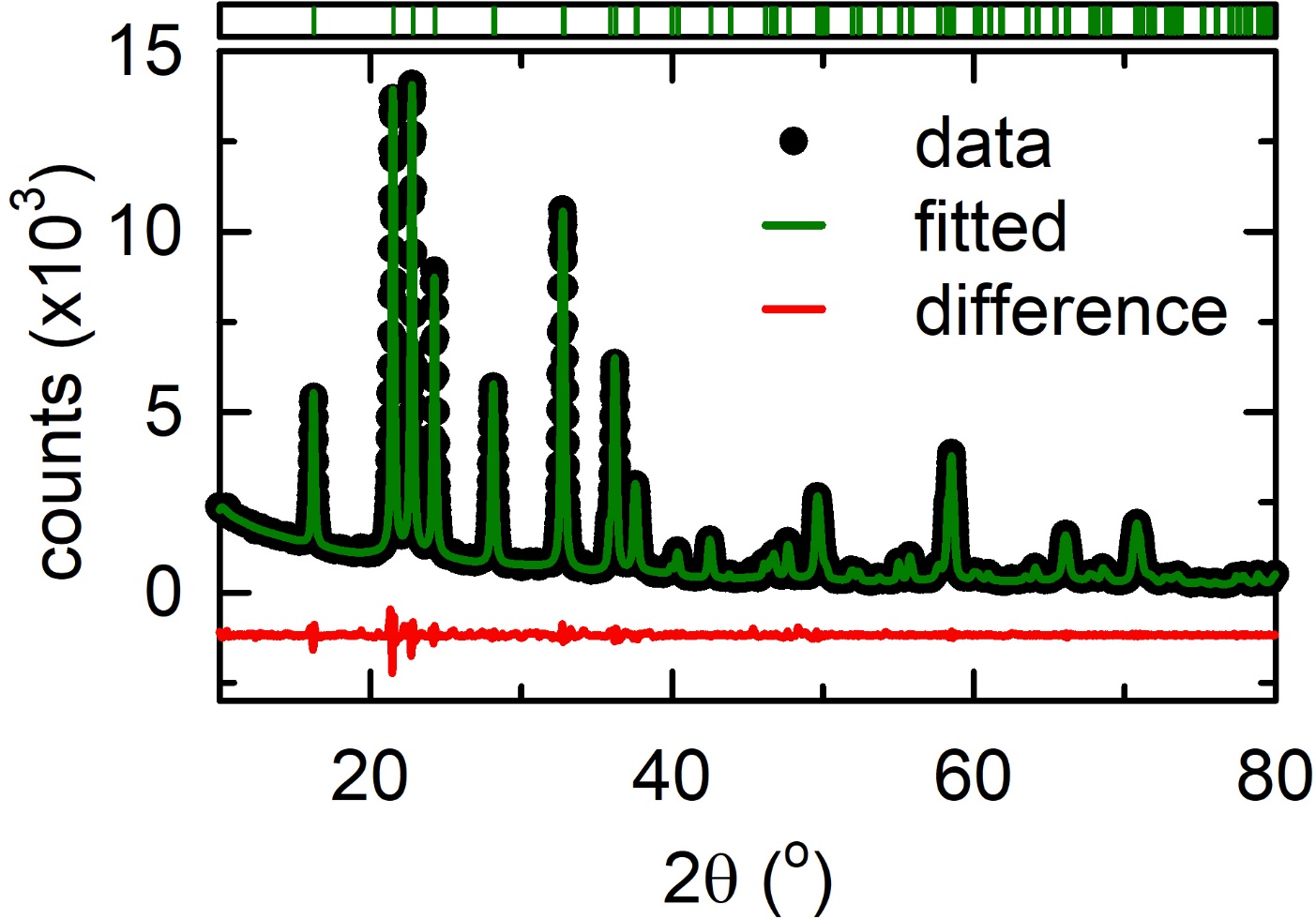
**

**Supplementary Figure 1.** Rietveld refinement based on HR-XRD of 1LCVO-ABR.

**
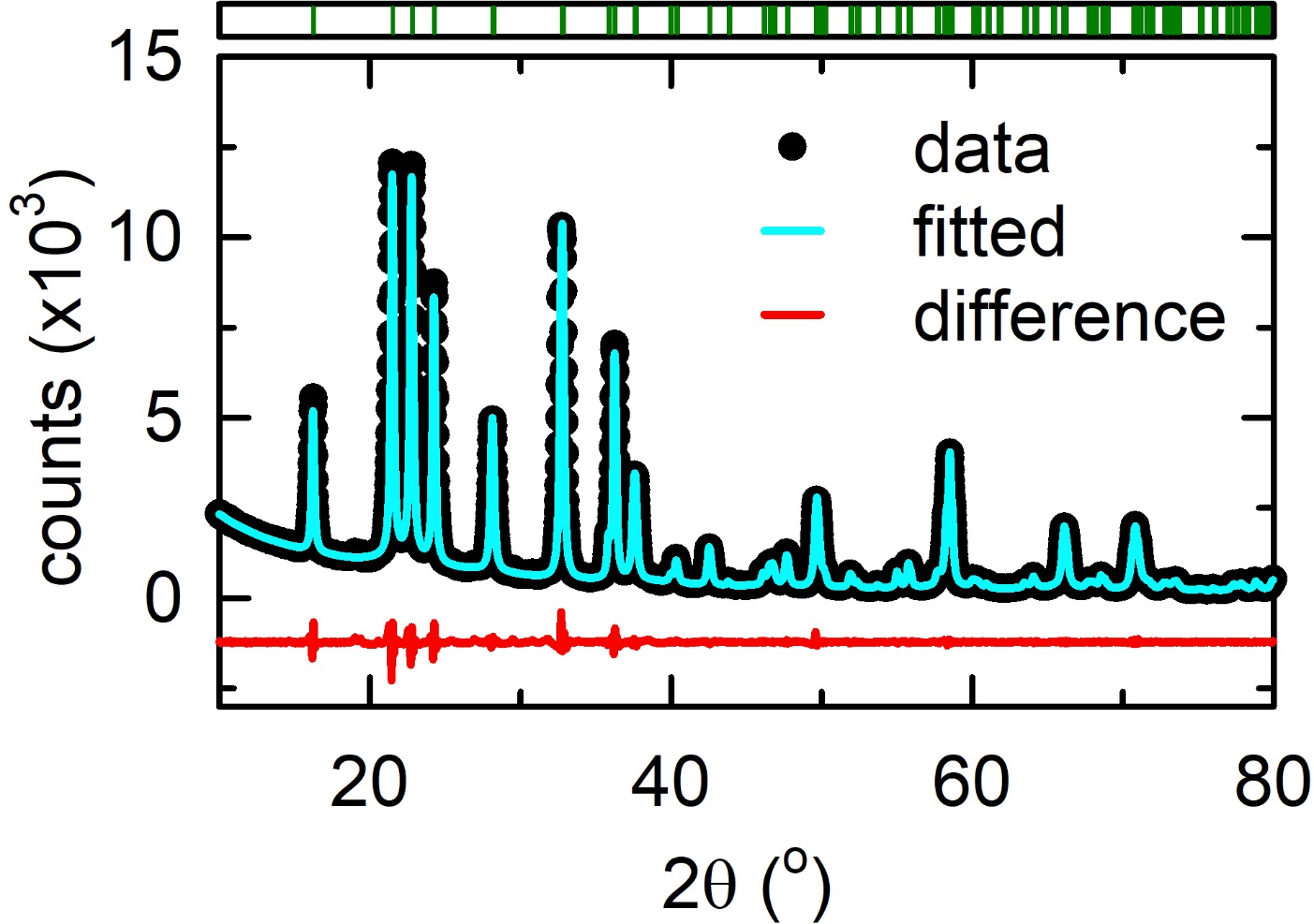
**

**Supplementary Figure 2.** Rietveld refinement based on HR-XRD of 2LCVO-ABR.


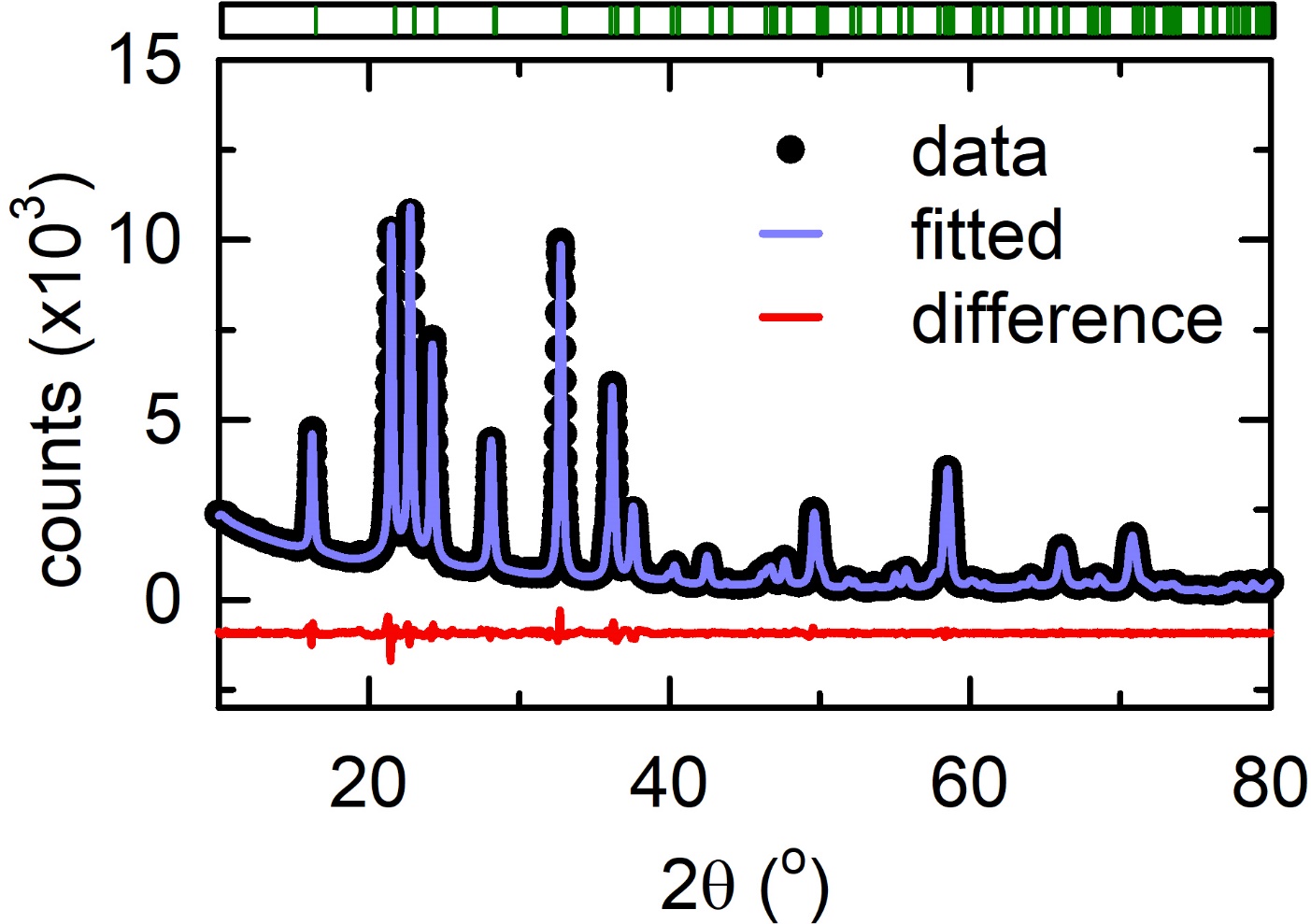


**Supplementary Figure 3.** Rietveld refinement based on HR-XRD of 5LCVO-ABR.


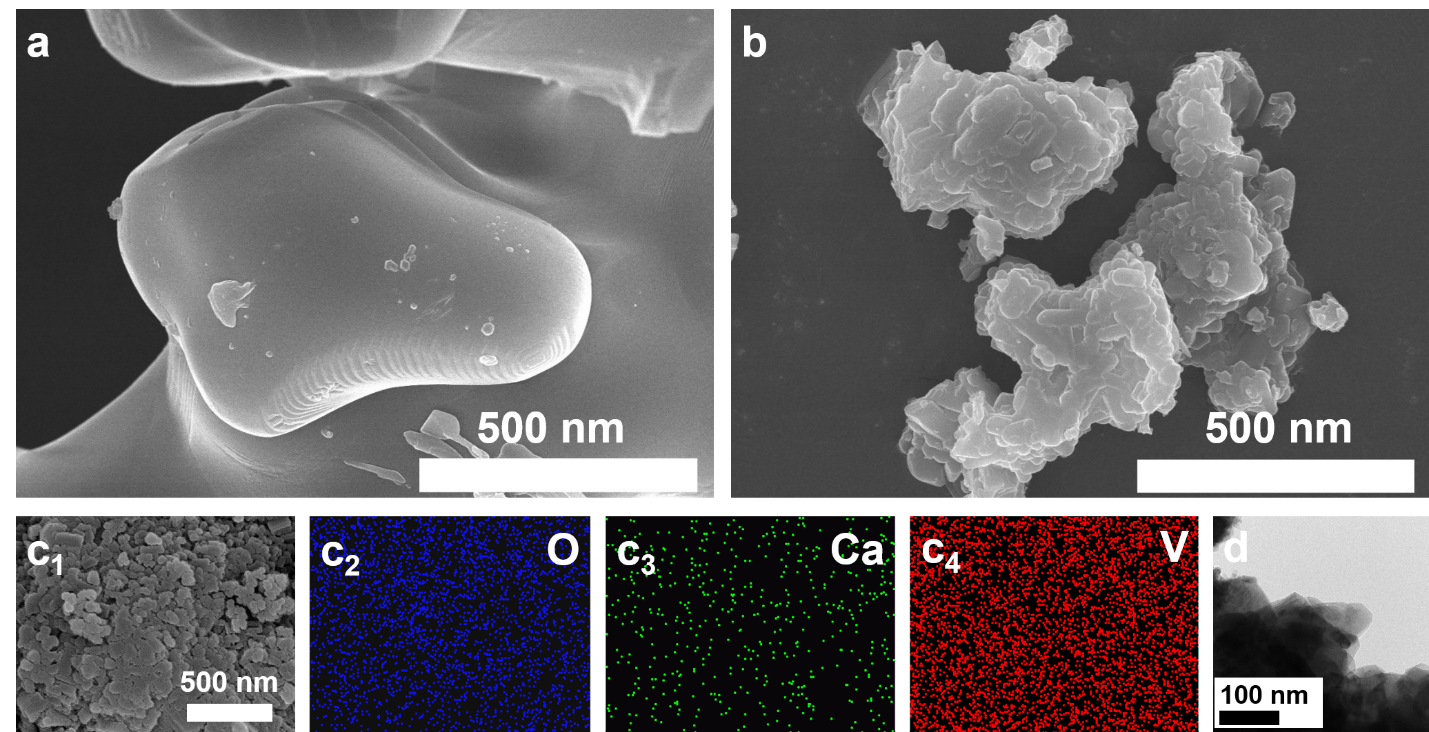


**Supplementary Figure 4.** SEM images of (a) 0LCVO-SSR and (b) 3LCVO-SSR; (c1) SEM images of mapping area, EDS mapping of 3LCVO-ABR for (c2) oxygen, (c3) calcium and (c4) vanadium; (d) TEM image of 0LCVO-ABR.


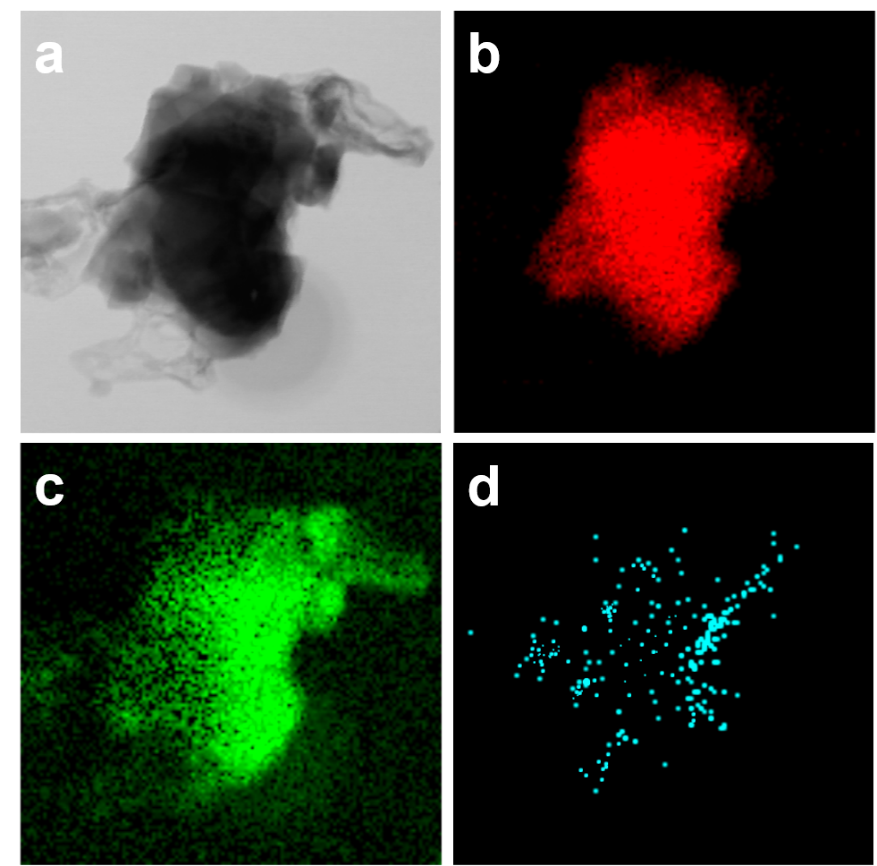


**Supplementary Figure 5.** TEM elemental mapping of 3LCVO: (a) TEM image of mapping area; mapping elements as (b) V; (c) O; and (d) Ca.


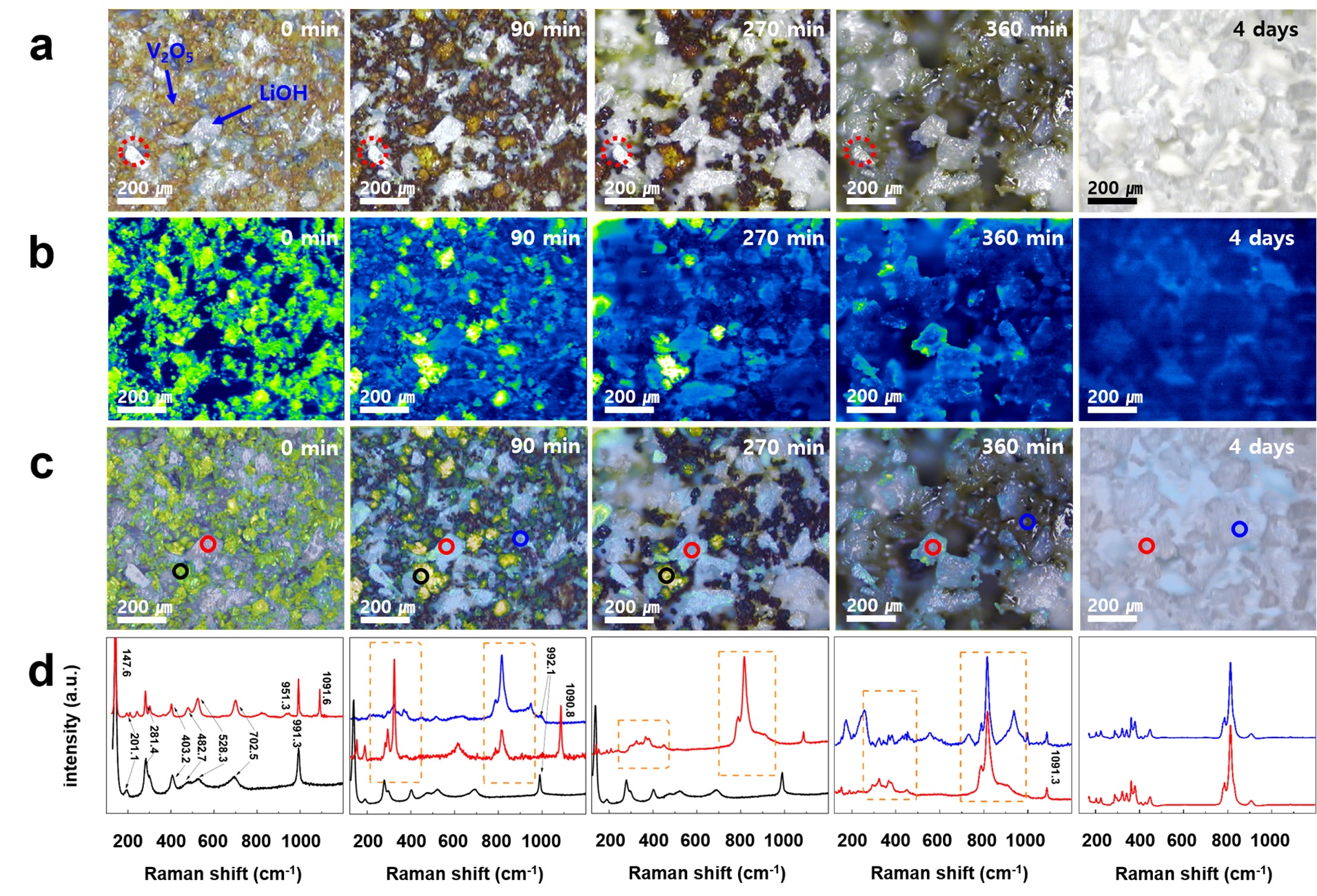


**Supplementary Figure 6.**  The digital photographs of synthesized sample Li_3_VO­_4_ under (a) naked eyes, (b) laser green light, (c) white light, and (d) the respective in situ Raman spectra at interval times during ABR synthesis process.


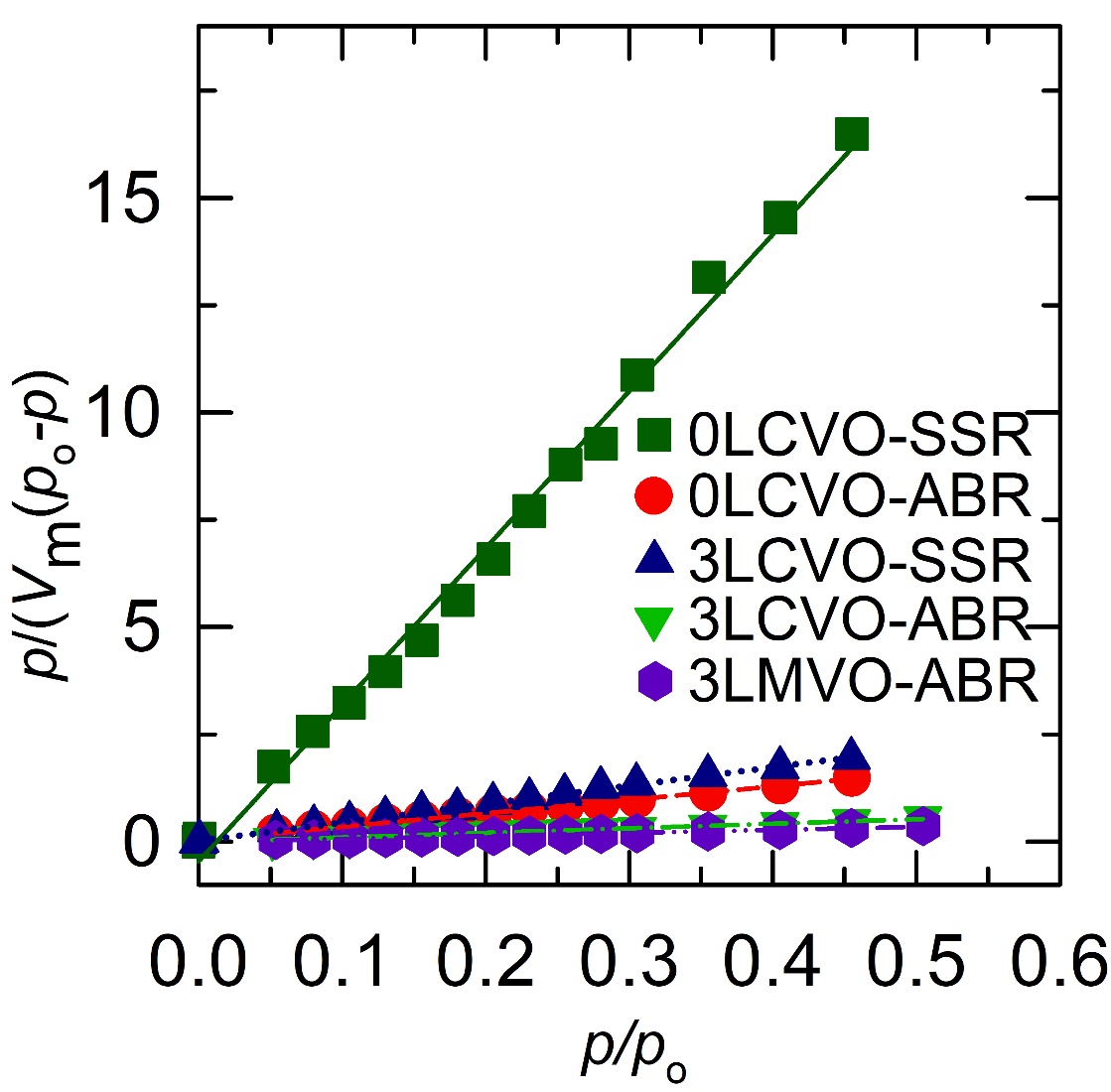


**Supplementary Figure 7.** The BET linear plot of as-prepared samples.


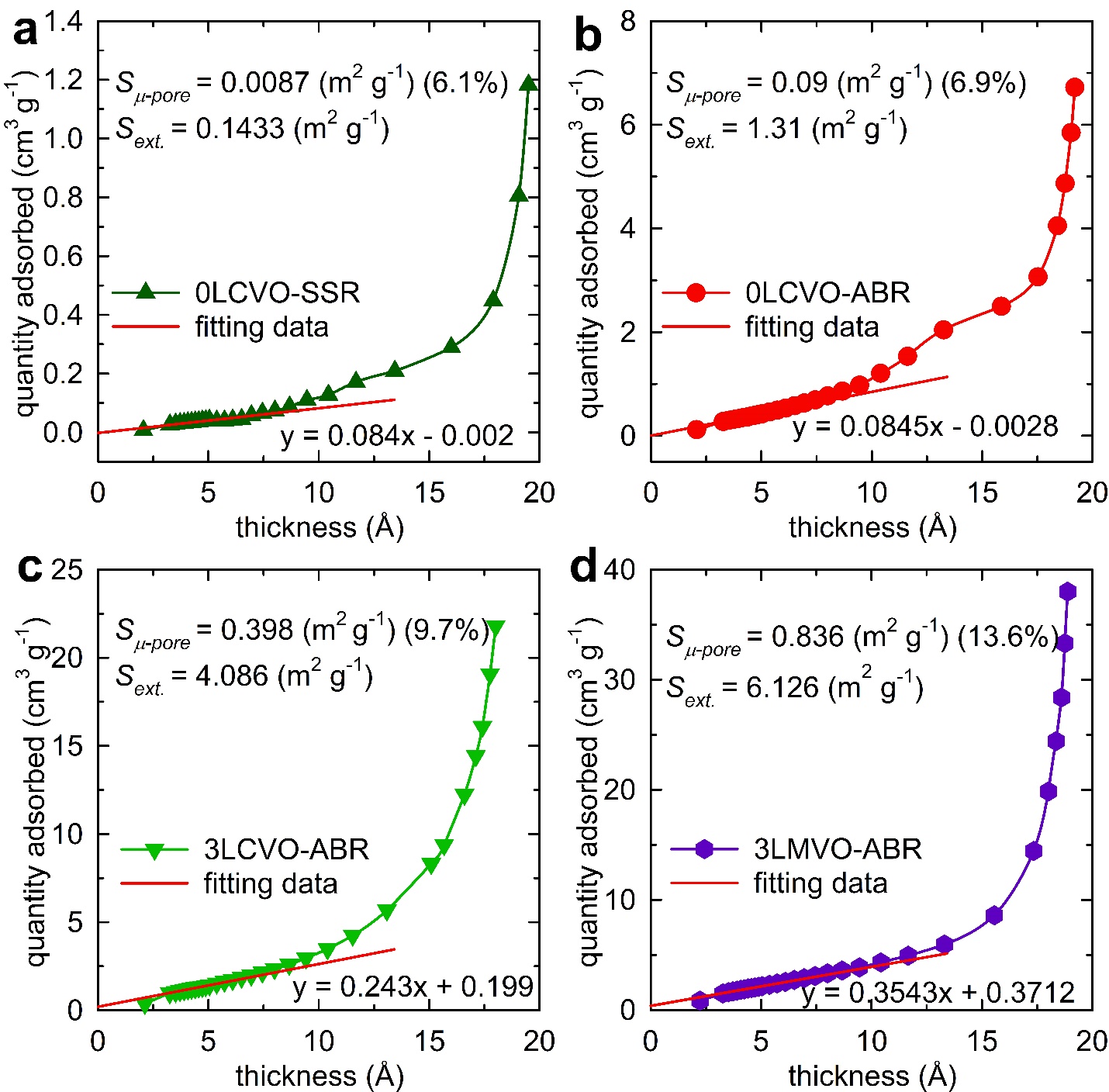


**Supplementary Figure 8.** *t*-plot for determination of external and m-pore surface area of (a) 0LCVO-SSR; (b) 0LCVO-ABR; (c) 3LCVO-ABR and (d) 3LMVO-ABR.


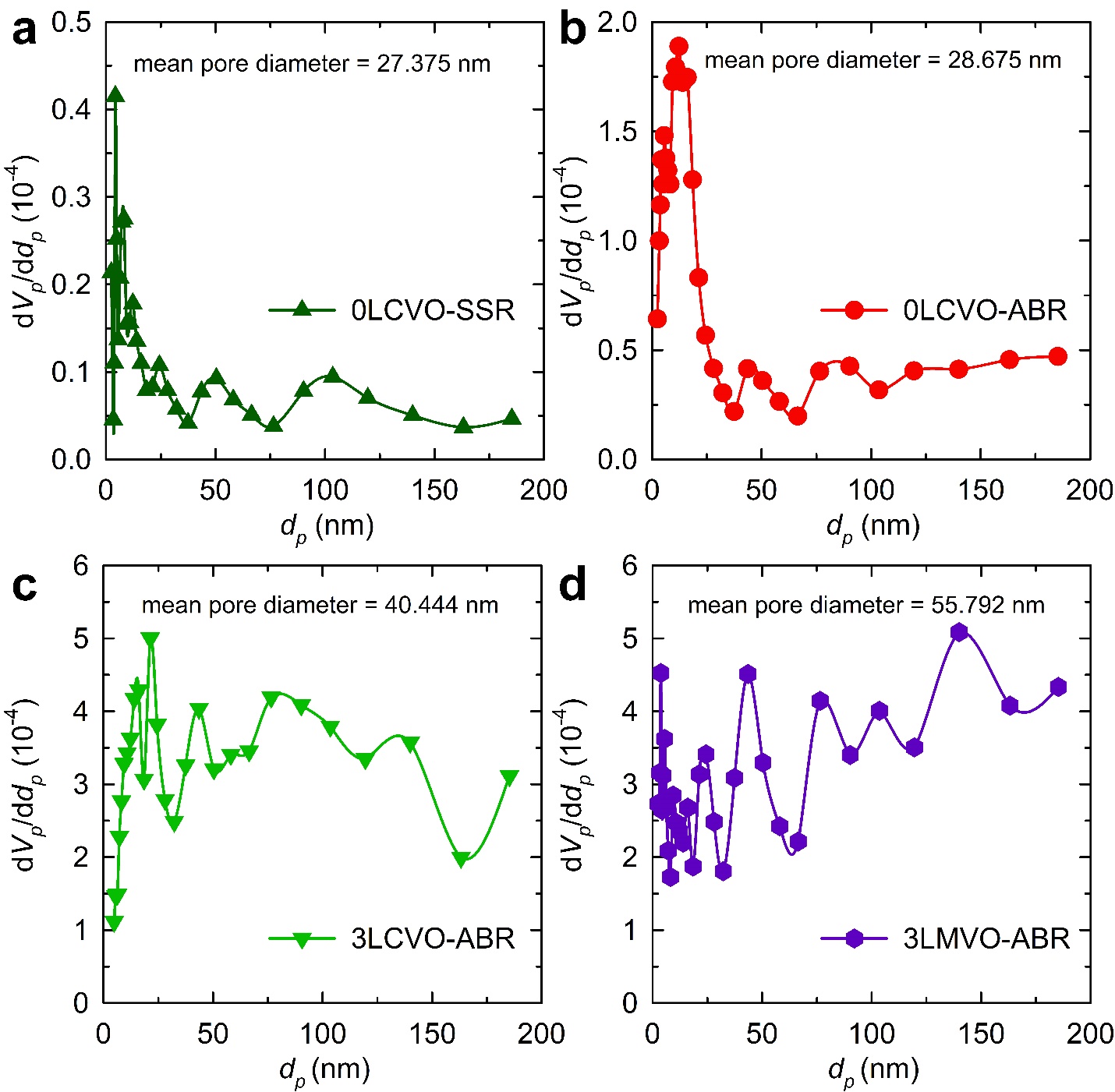


**Supplementary Figure 9.** BJH pore size distribution derived from BET data of (a) 0LCVO-SSR; (b) 0LCVO-ABR; (c) 3LCVO-ABR and (d) 3LMVO-ABR.


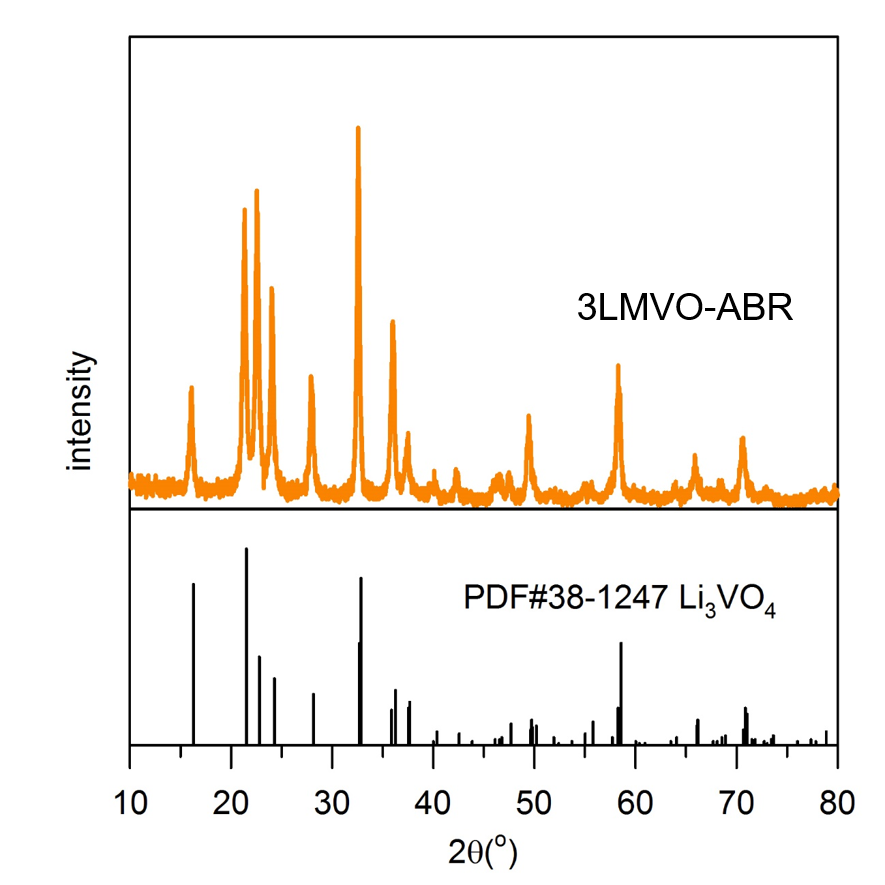


**Supplementary Figure 10.** XRD pattern of 3LMVO-ABR.


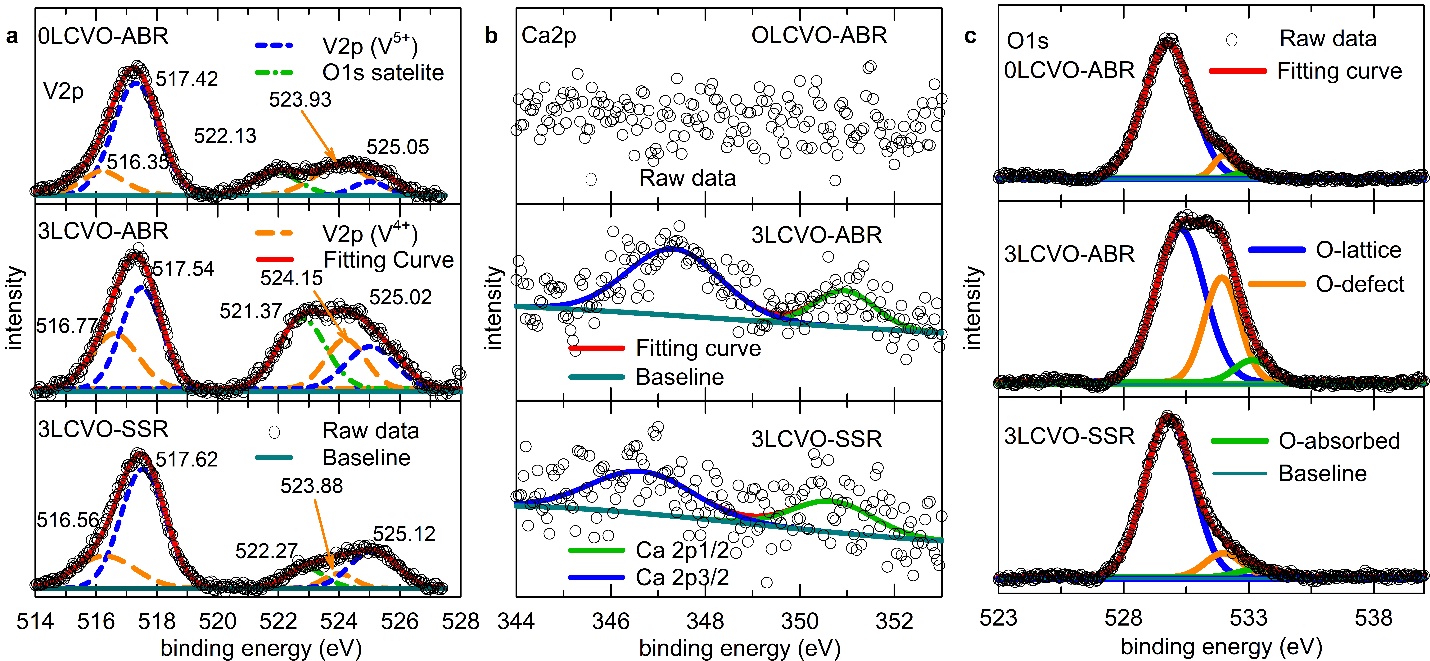


**Supplementary Figure 11.** High resolution XPS spectra of (a) V2*p*, (b) Ca2*p*, and (c) O1*s* of 0LCVO-ABR, 3LCVO-ABR and 3LCVO-SSR.


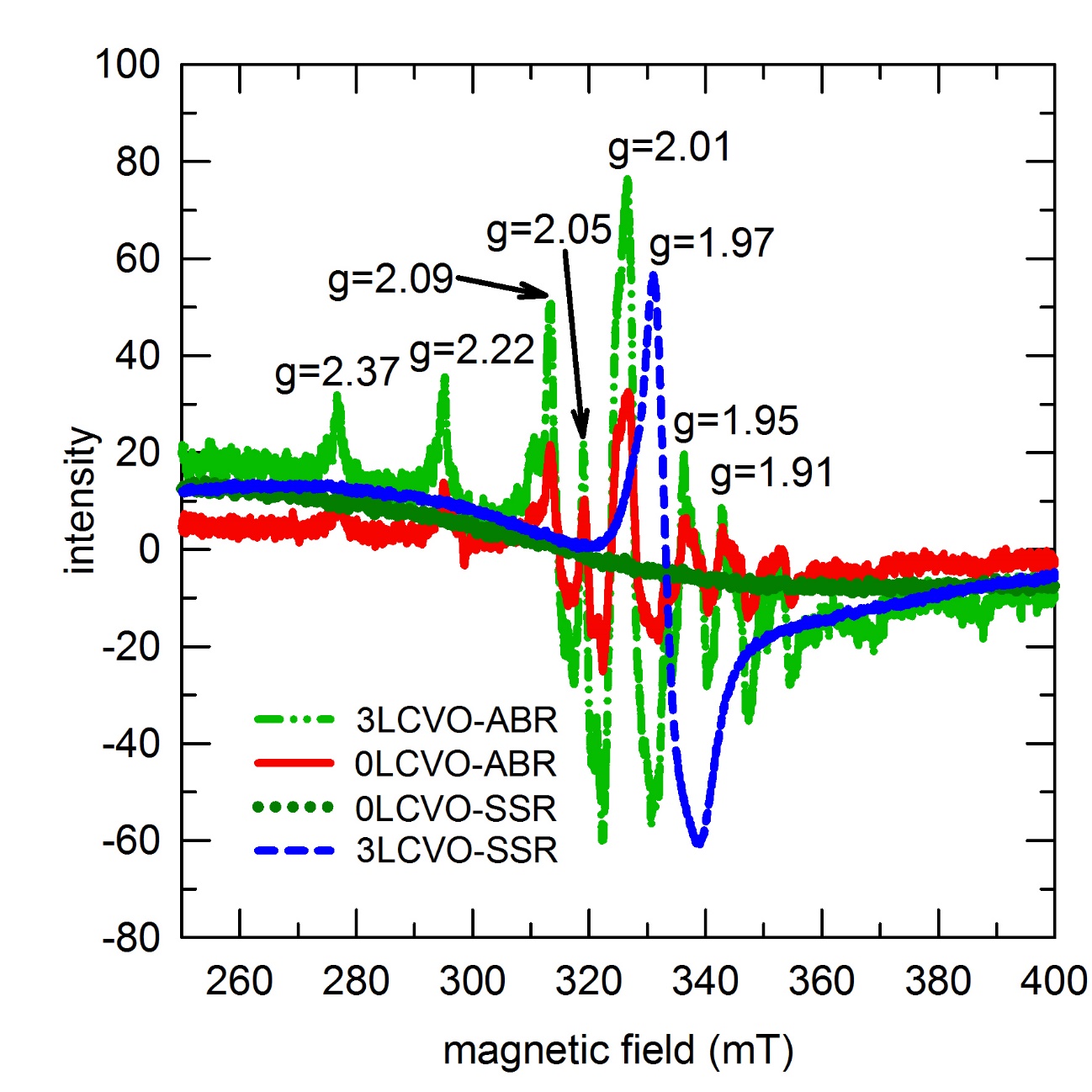


**Supplementary Figure 12.** EPR spectra of as-prepared sample.


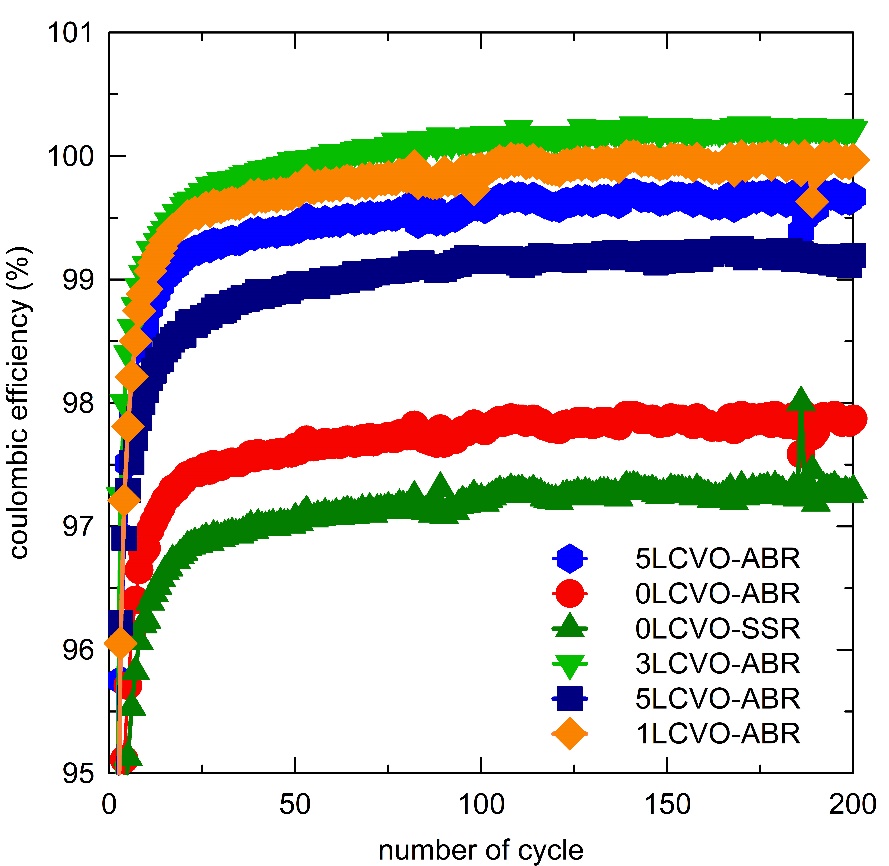


**Supplementary Figure 13.** Enlargement of coulombic efficiency of xLCVO-ABR and –SSR at current density of 100 mA⸳g^-1^.


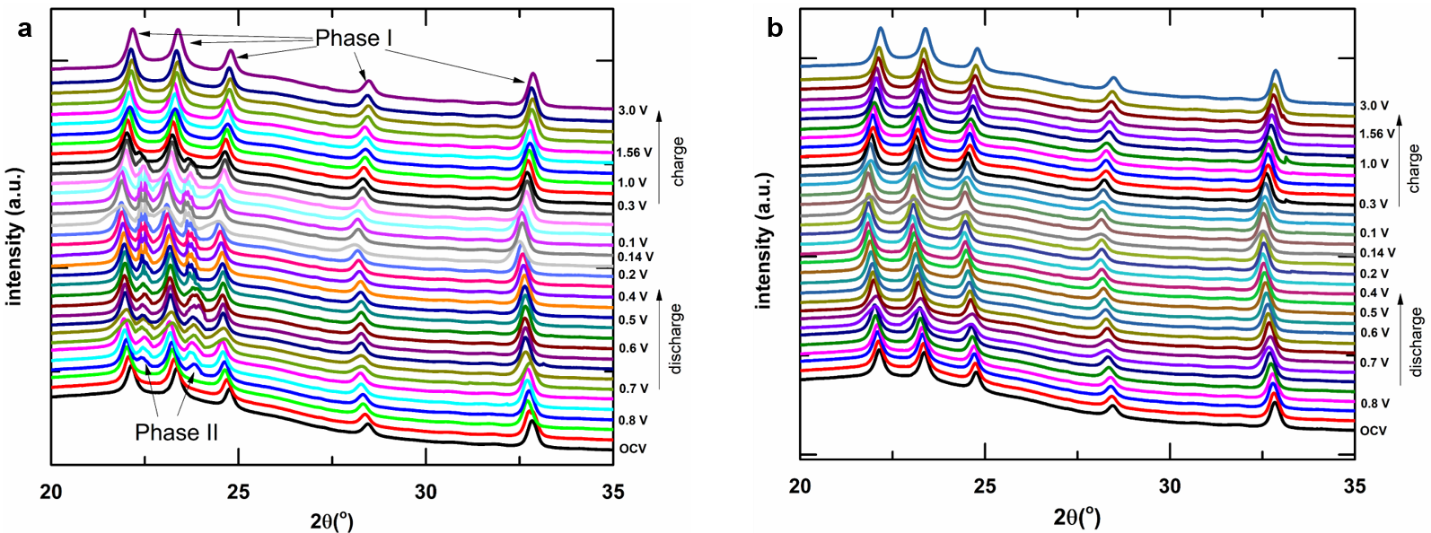


**Supplementary Figure 14.** *In situ* XRD of (a) 0LCVO-ABR; and (b) 3LCVO-ABR for the first cycle of discharge/charge in 2θ-range of 20 - 35°.


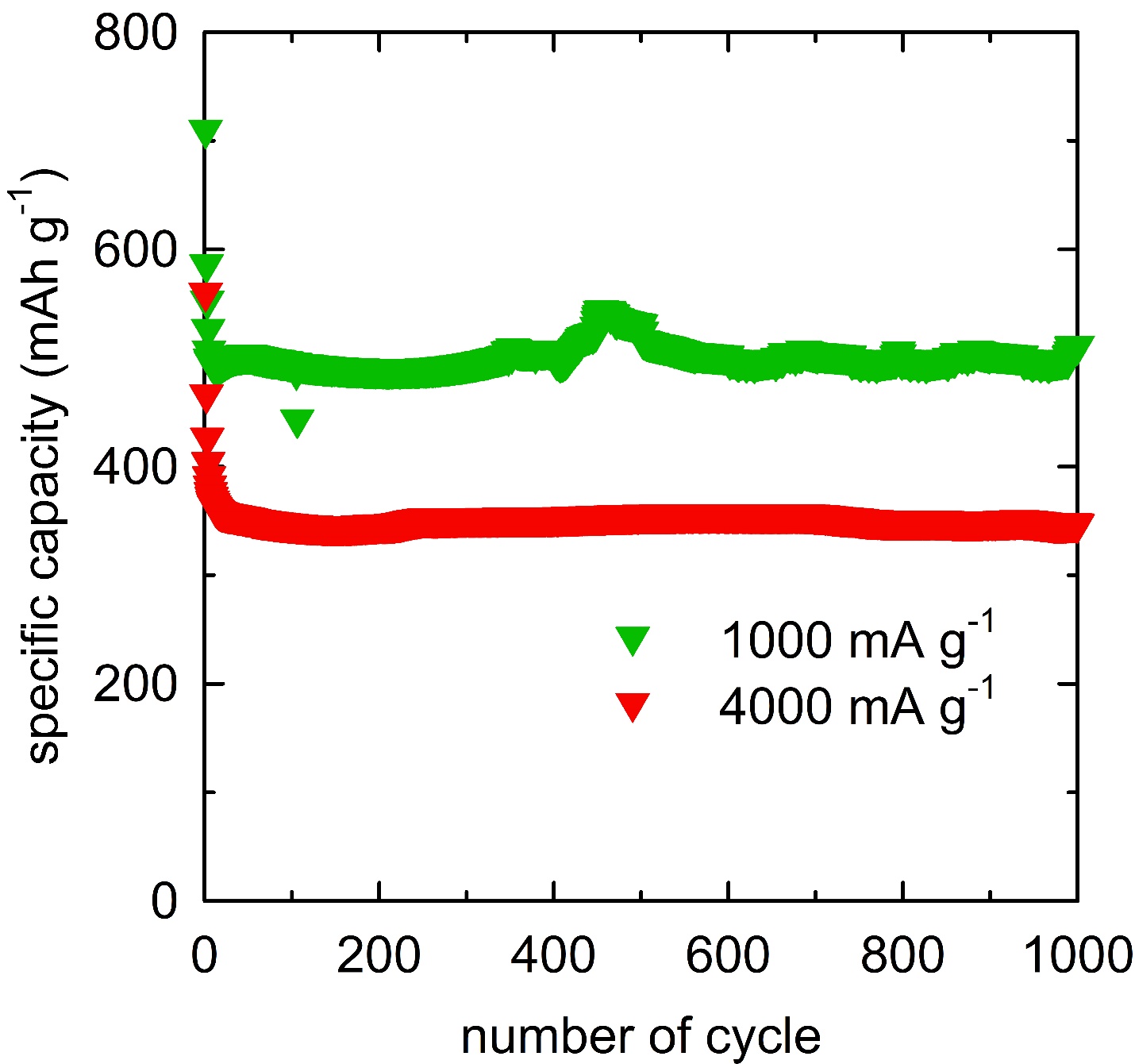


**Supplementary Figure 15.** Cycling performance of 3LCVO-ABR at current density of 1000 and 4000 mA⸳g^-1^.


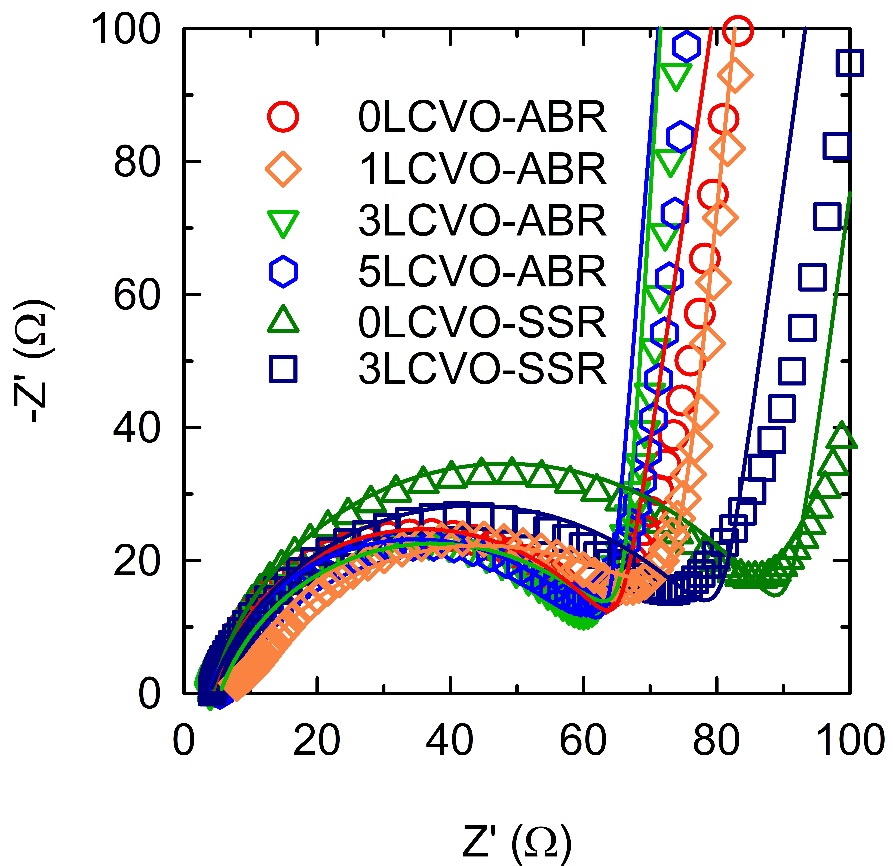


**Supplementary Figure 16.** Enlargement of Nyquist plots from EIS fitting data.


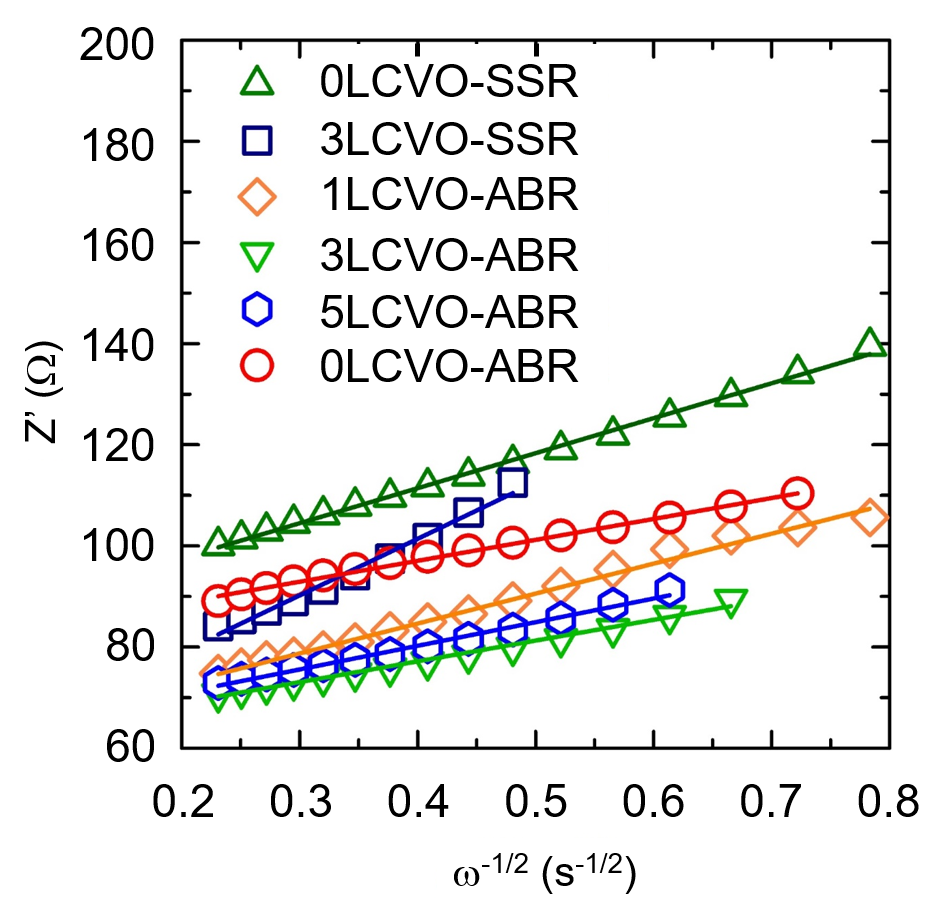


**Supplementary Figure 17.** The linear fitting of the real impedance Z’ vs. ω-1/2 relationship of all as-prepared samples.


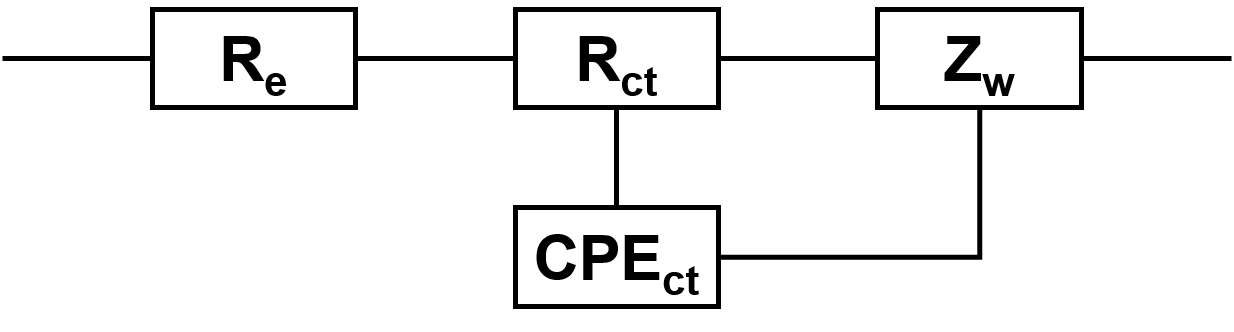


**Supplementary Figure 18.** Equivalent circuit model used for fitting EIS spectra.


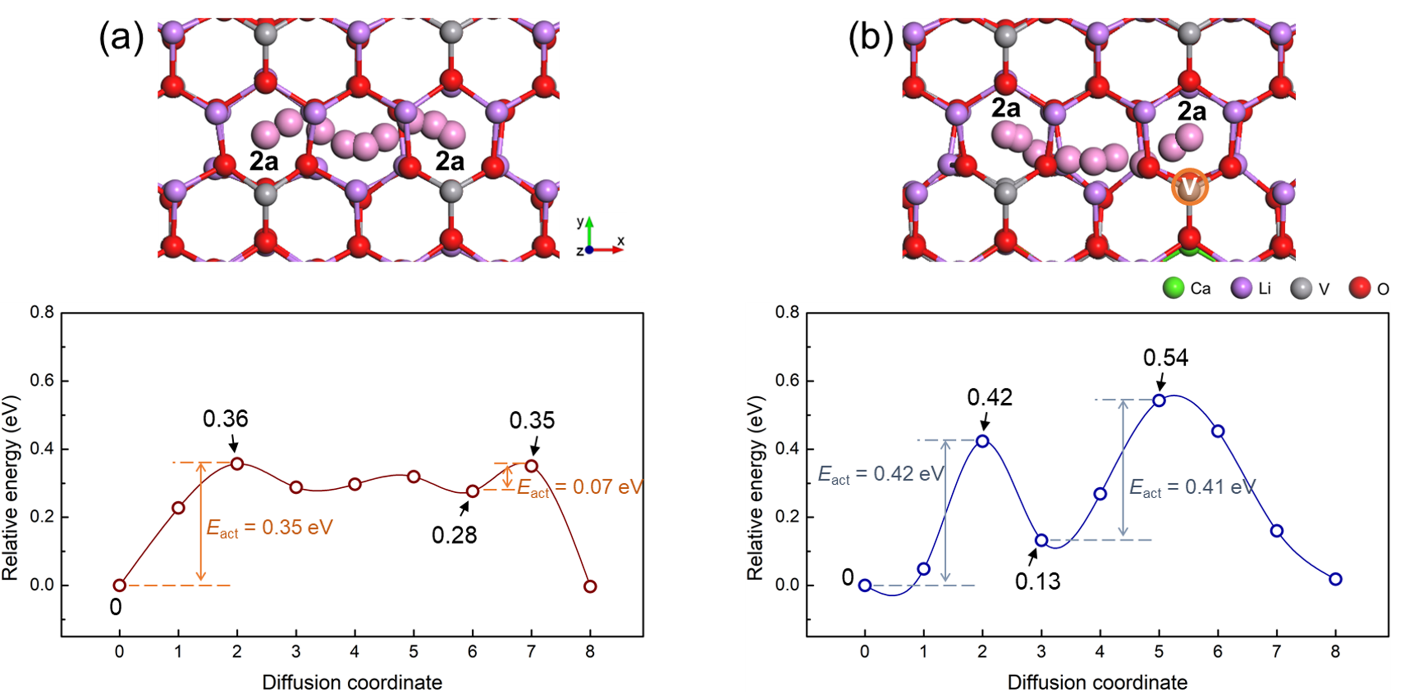


**Supplementary Figure 19.** DFT-estimated parallel diffusion pathway of Li ion from the Li^2a^ to the adjacent Li^2a^ site in (a) a Li_48_V_16_O_64_ or (b) a Li_46_Ca_2_V_16_O_63_ model. The migrating Li ion is colored in pink. The black colored numbers present the relative energy of the corresponding state. The *E*_act_ denotes the diffusion energy barrier of each step.


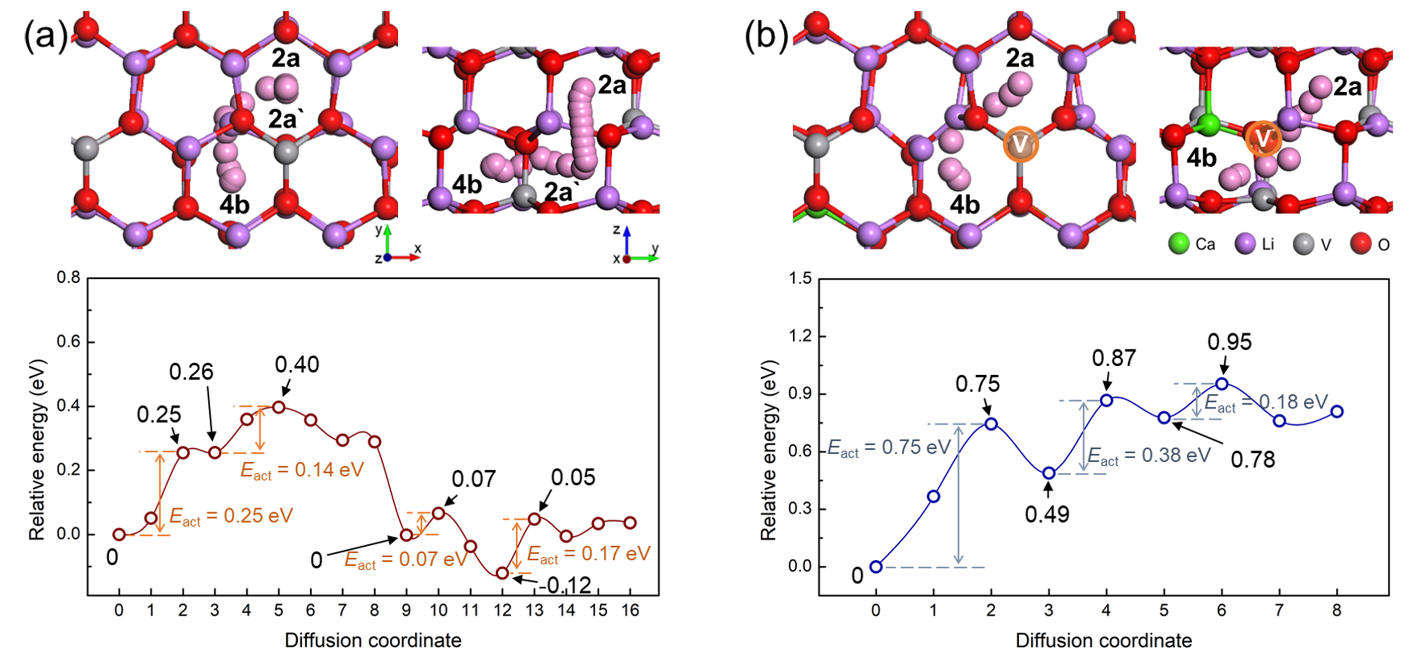


**Supplementary Figure 20.** DFT-estimated perpendicular diffusion pathway of Li ion from the Li^2a^ to the adjacent Li^4b^ site in (a) a Li_48_V_16_O_64_ or (b) a Li_46_Ca_2_V_16_O_63_ model. The migrating Li ion is colored in pink. The black colored numbers present the relative energy of the corresponding state. The *E*_act_ denotes the diffusion energy barrier of each step.


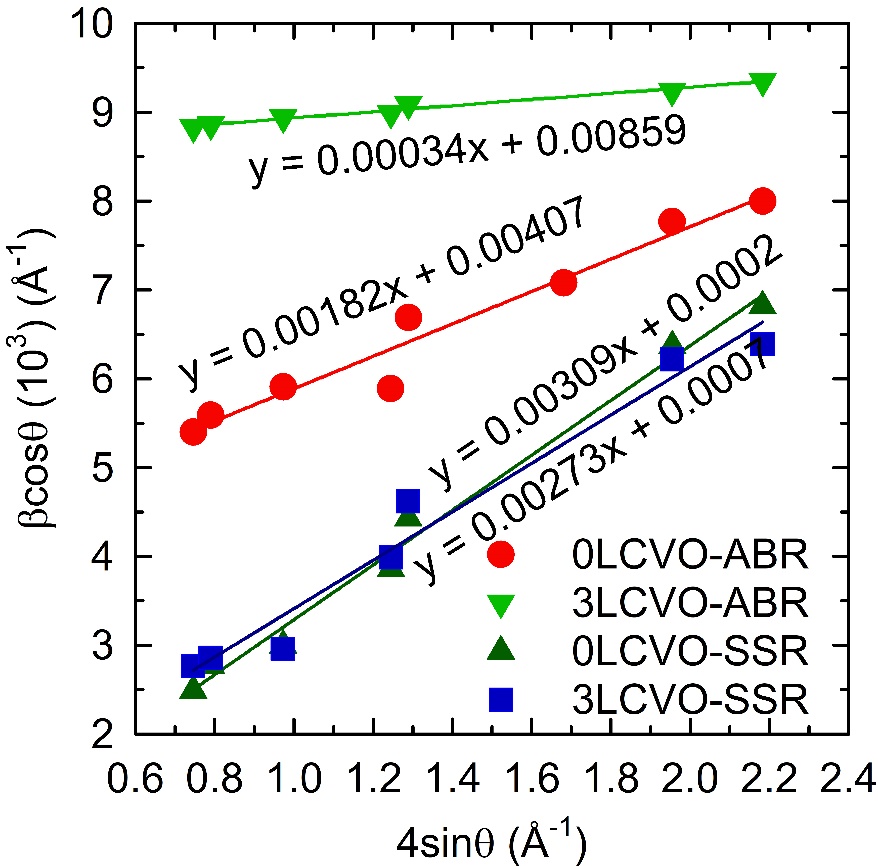


**Supplementary Figure 21.** Williamson−Hall (WH) plot of the xLCVO-ABR and -SSR samples based on the XRD pattern showing the lattice strain and crystallite size variation. WH equation: βcosθ = Kλ/D + 4εsinθ, in which β is full width at half maximum of the diffraction peak (rad); θ is Bragg angle (rad); K is shape factor (Scherrer constant approximately 1), λ is wavelength of X-ray source (1.5418 Å); D is crystallite size (Å); and ε is microstrain.


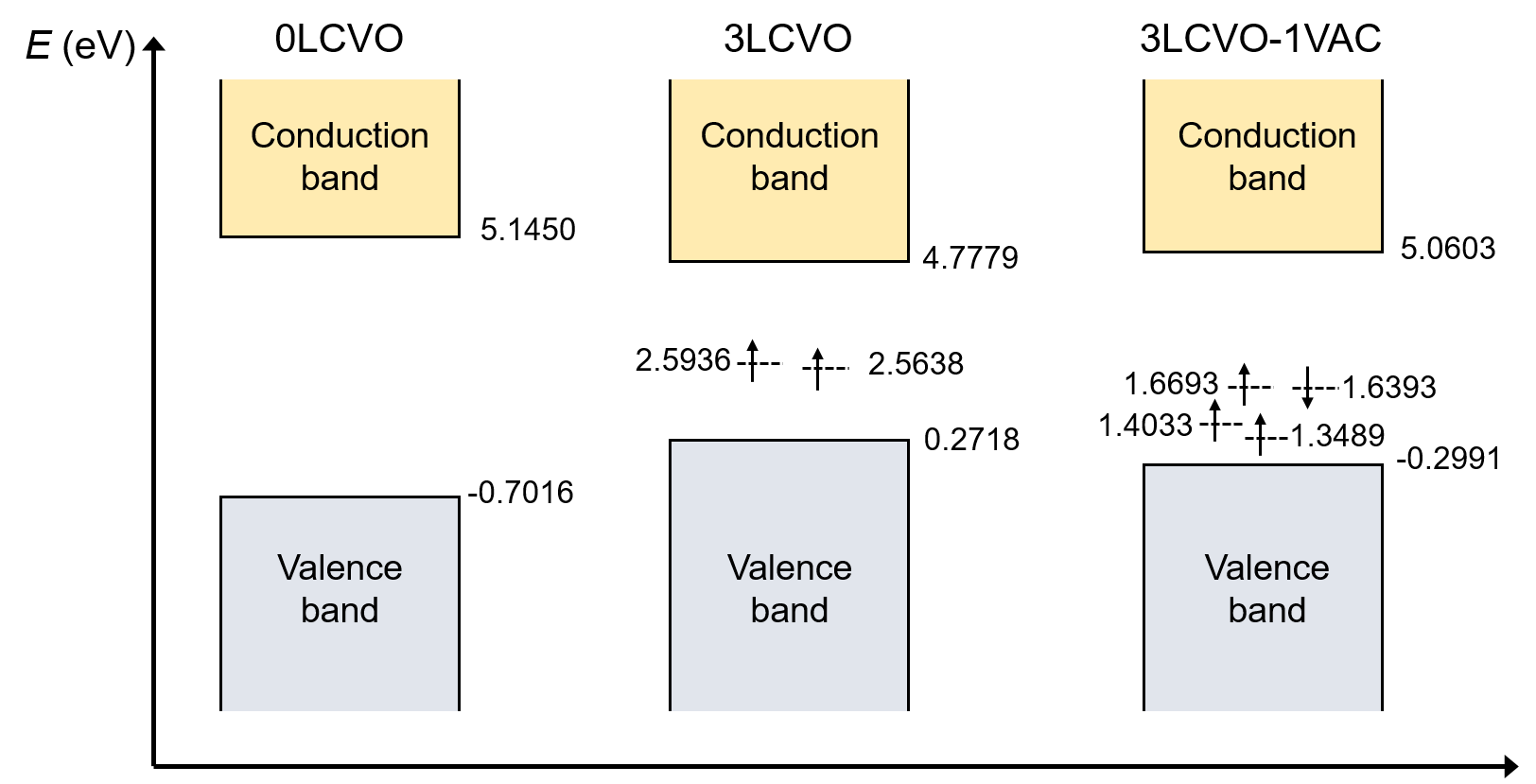


**Supplementary Figure 22.** Band diagram derived from DFT calculation for pure Li_3_VO­_4_ (0LCVO); Ca-doped Li_3_VO_4_ (3LCVO); and Ca-doped Li_3_VO_4_ with oxygen vacancy (3LCVO-1VAC).


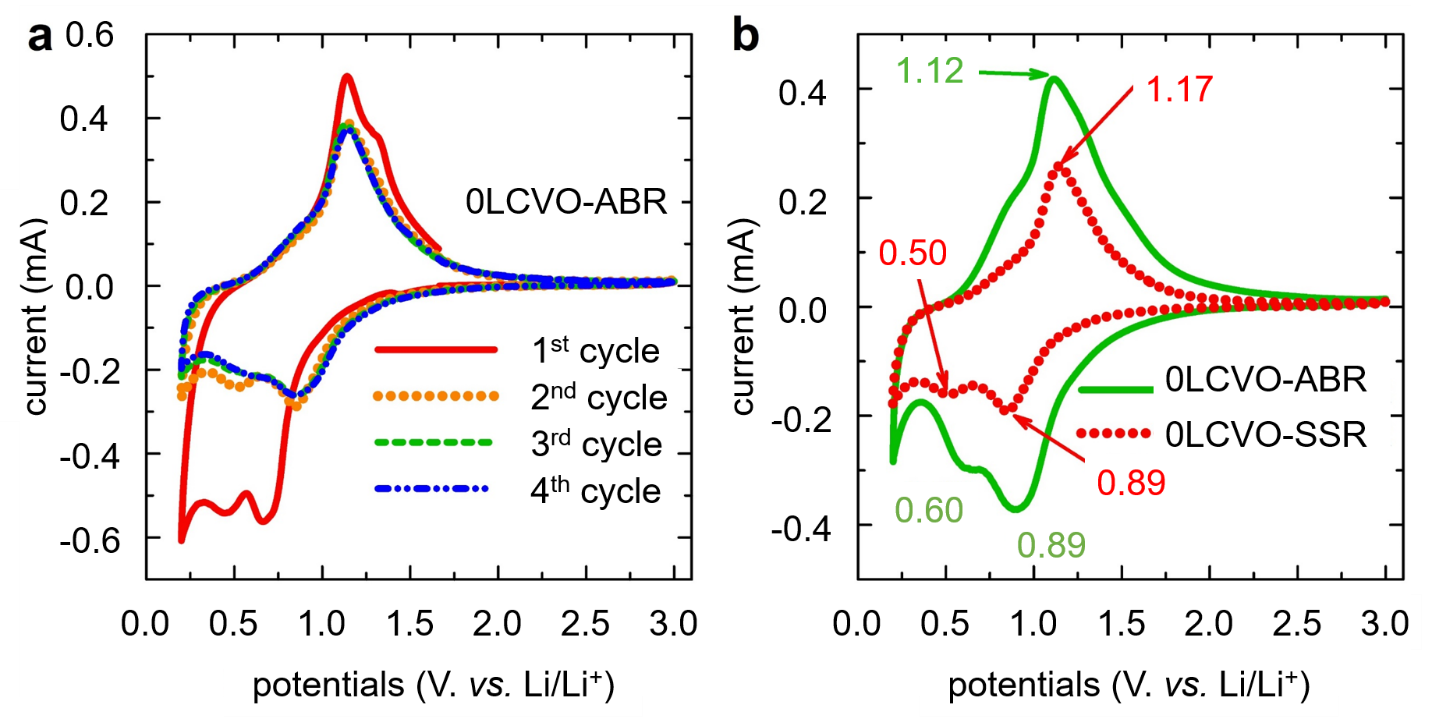


**Supplementary Figure 23.** (a) Four first CV cycles of 0LCVO-ABR and (b) comparison of the 2nd CV curves of 0LCVO-ABR and 3LCVO-ABR.

**
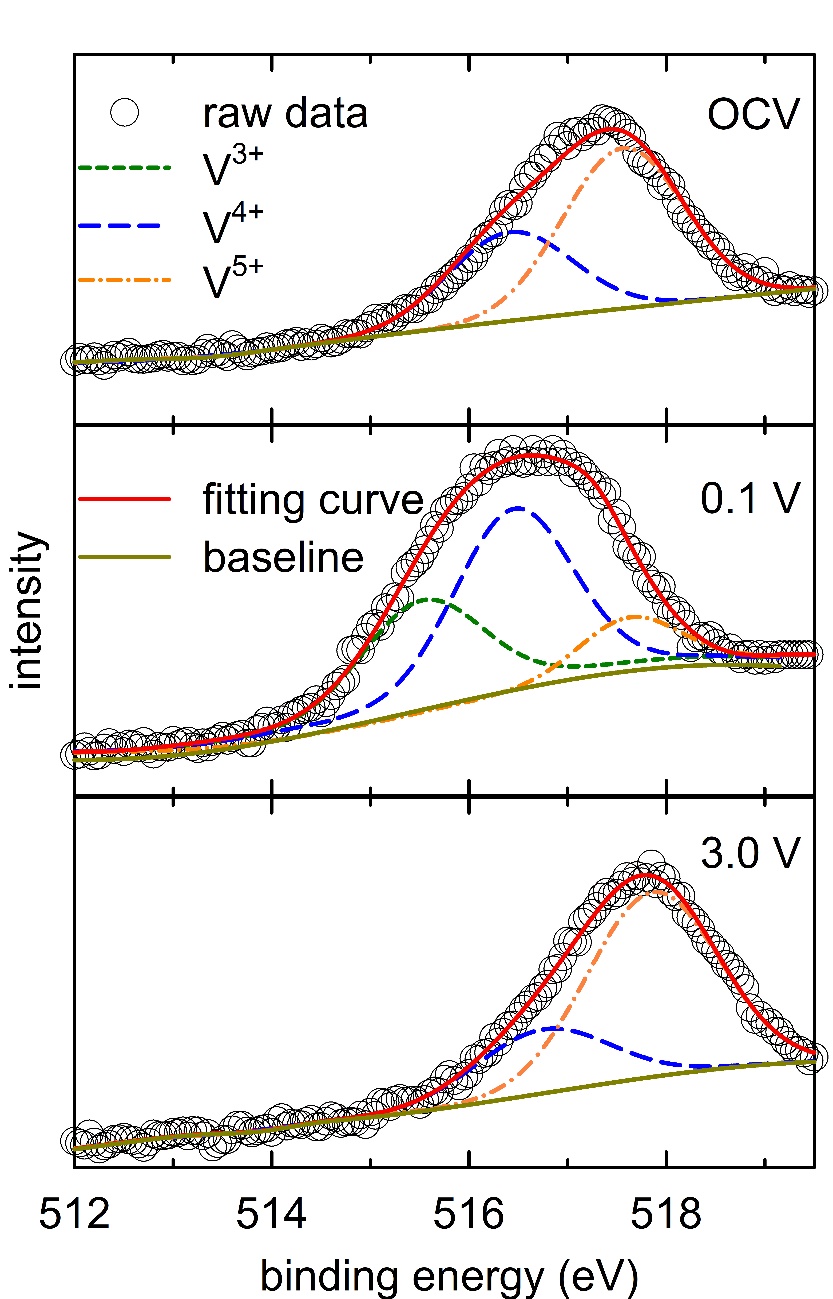
**

**Supplementary Figure 24.** *Ex situ* XPS of V2p of 3LCVO-ABR electrode at initial state, after discharged to 0.1 V, and charged to 3.0 V, *vs.* Li/Li^+^.


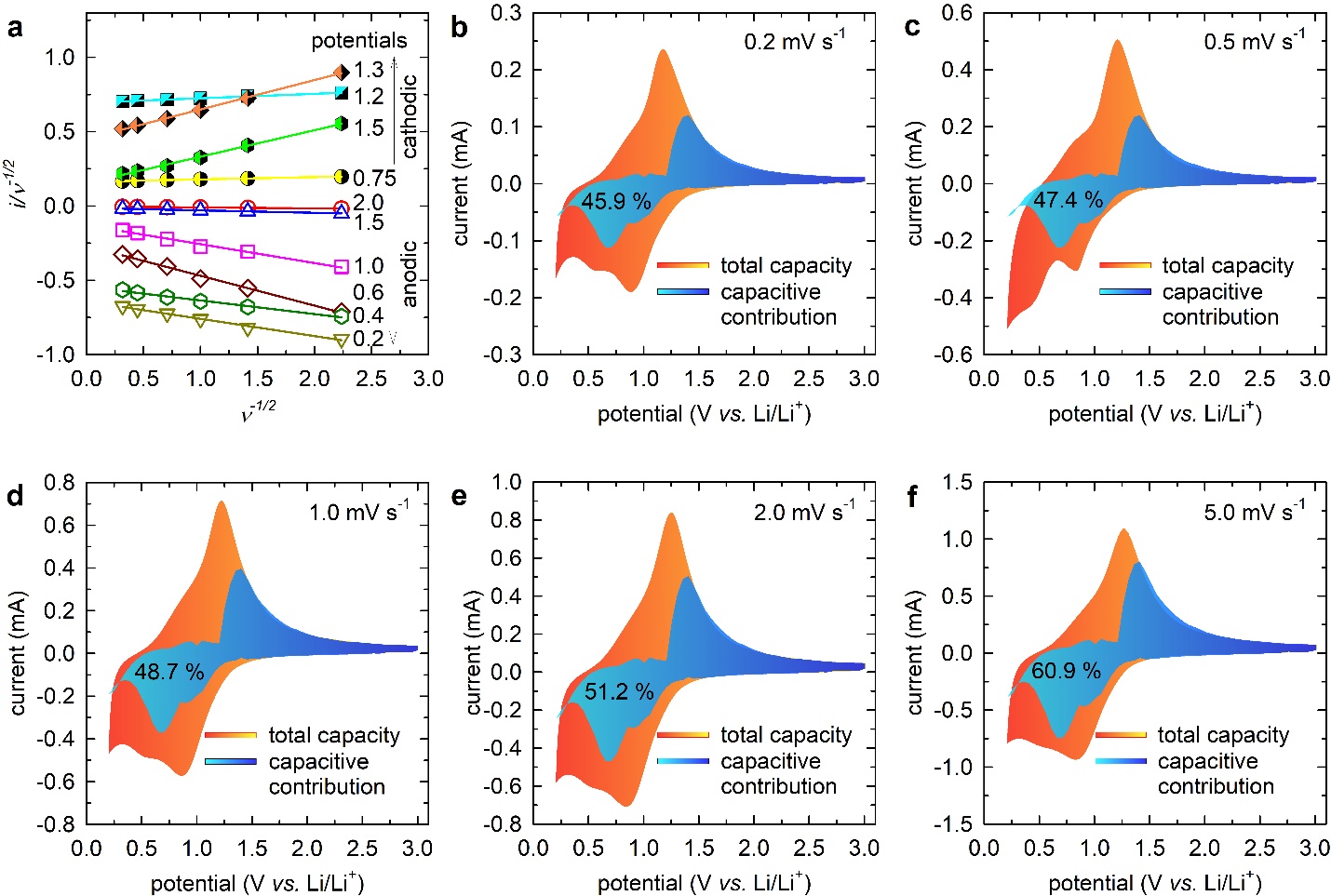


**Supplementary Figure 25.** (a) The linear plot of i/ν^1/2^ vs. ν^1/2^ for calculation of k-constants; (b-f) pictorial pseudocapacitance contribution calculation at various potential values.

**Supplementary Table 1.** Comparison on lattice parameters and unit cell volume of *x*LCVO-ABR derived from Rietveld refinement. Note: The numbers in parentheses are the estimated standard deviations of the last significant figure.

| samples | 0LCVO-ABR | 1LCVO-ABR | 2LCVO-ABR | 3LCVO-ABR | 5LCVO-ABR |
| --- | --- | --- | --- | --- | --- |
| symmetry | orthorhombic | | | | |
| space grpoup | *Pmn*2_1_ | | | | |
| *a* (*Å*) | 6.3106(3) | 6.3179(1) | 6.3183(4) | 6.3202(5) | 6.3228(3) |
| *b* (*Å*) | 5.4412(2) | 5.4460(5) | 5.4479(4) | 5.4484(4) | 5.4517(1) |
| *c* (*Å*) | 4.9483(2) | 4.9492(2) | 4.9509(3) | 4.9520(2) | 4.9540(3) |
| V (*Å^3^*) | 169.916(2) | 170.250(1) | 170.352(2) | 170.462(3) | 170.515(6) |
| *χ^2^* | 1.724 | 1.707 | 1.295 | 1.639 | 1.211 |
| *R_wp_ (%)* | 2.50 | 4.04 | 3.58 | 2.58 | 3.40 |
| *R_p_ (%)* | 1.95 | 3.26 | 2.91 | 2.00 | 3.37 |

**Supplementary Table 2.** Structural parameters of 1LCVO-ABR as obtained from the Rietveld
refinement of X-ray. Note: The numbers in parentheses are the estimated standard deviations of the last significant figure.

| atom | Wyckoff position | *x* | *y* | *z* | 100 $\times$ *U_iso_ (*Å^2^*)*^a^ | *g*^b^ |
| --- | --- | --- | --- | --- | --- | --- |
| O1 | 4*b* | 0.2172(6) | 0.6889(11) | 0.9172(12) | 0.074(3) | 1.0 |
| O2 | 2*a* | 0.0 | 0.1196(1) | 0.9129(13) | 0.073(2) | 1.0 |
| O3 | 2*a* | 1/2 | 0.1844(11) | 0.8475(8) | 0.158(6) | 1.0 |
| Li1 | 4*b* | 0.2507(1) | 0.3457(2) | 0.9662(4) | 0.226(1) | 0.99 |
| Li2 | 2*a* | 1/2 | 0.8472(2) | 0.9654(3) | 0.318(3) | 0.99 |
| V1 | 2*a* | 0.0 | 0.8314(5) | -0.0022(0) | 0.165(5) | 1.0 |
| Ca1 | 4*b* | 0.2507(1) | 0.3457(2) | 0.9662(4) | 0.226(2) | 0.01 |
| Ca2 | 2*a* | 1/2 | 0.8472(2) | 0.9654(3) | 0.318(4) | 0.01 |

^a^  Constraint on isotropic thermal factor: U_iso_(Li) = U_iso_(Ca)

^b^ Constraint on occupancy: g(Li) + g(Ca) = 1.0

**Supplementary Table 3.** Structural parameters of 2LCVO-ABR as obtained from the Rietveld
refinement of X-ray. Note: The numbers in parentheses are the estimated standard deviations of the last significant figure.

| atom | Wyckoff position | *x* | *y* | *z* | 100 $\times$ *U_iso_ (*Å^2^*)*^a^ | *g*^b^ |
| --- | --- | --- | --- | --- | --- | --- |
| O1 | 4*b* | 0.2217(4) | 0.6847(8) | 0.9012(7) | 0.148(2) | 1.0 |
| O2 | 2*a* | 0.0 | 0.1249(9) | 0.8961(13) | 0.043(3) | 1.0 |
| O3 | 2*a* | 1/2 | 0.1761(1) | 0.8439(6) | 0.290(1) | 1.0 |
| Li1 | 4*b* | 0.2489(8) | 0.3303(2) | 0.9580(2) | 0.346(5) | 0.98 |
| Li2 | 2*a* | 1/2 | 0.8345(2) | 0.9616(3) | 0.435(4) | 0.98 |
| V1 | 2*a* | 0.0 | 0.8299(3) | -0.0008(0) | 0.265(2) | 1.0 |
| Ca1 | 4*b* | 0.2489(8) | 0.3303(2) | 0.9580(2) | 0.346(6) | 0.02 |
| Ca2 | 2*a* | 1/2 | 0.8345(2) | 0.9616(3) | 0.435(2) | 0.02 |

^a^  Constraint on isotropic thermal factor: U_iso_(Li) = U_iso_(Ca)

^b^ Constraint on occupancy: g(Li) + g(Ca) = 1.0

**Supplementary Table 4.** Structural parameters of 5LCVO-ABR as obtained from the Rietveld
refinement of X-ray. Note: The numbers in parentheses are the estimated standard deviations of the last significant figure.

| atom | Wyckoff position | *x* | *y* | *z* | 100 $\times$ *U_iso_ (*Å^2^*)*^a^ | *g*^b^ |
| --- | --- | --- | --- | --- | --- | --- |
| O1 | 4*b* | 0.2206(5) | 0.6899(1) | 0.9450(1) | 0.102(2) | 1.0 |
| O2 | 2*a* | 0.0 | 0.1208(1) | 0.9172(13) | 0.109(8) | 1.0 |
| O3 | 2*a* | 1/2 | 0.1846(2) | 0.8550(0) | 0.290(1) | 1.0 |
| Li1 | 4*b* | 0.2558(8) | 0.3281(2) | 1.0075(3) | 0.216(4) | 0.95 |
| Li2 | 2*a* | 1/2 | 0.8419(2) | 0.9537(2) | 0.198(2) | 0.95 |
| V1 | 2*a* | 0.0 | 0.8299(3) | -0.0008(0) | 0.265(3) | 1.0 |
| Ca1 | 4*b* | 0.2558(8) | 0.3281(2) | 1.0075(3) | 0.216(2) | 0.05 |
| Ca2 | 2*a* | 1/2 | 0.8419(2) | 0.9537(2) | 0.198(7) | 0.05 |

^a^  Constraint on isotropic thermal factor: U_iso_(Li) = U_iso_(Ca)

^b^ Constraint on occupancy: g(Li) + g(Ca) = 1.0

**Supplementary Table 5.**  The elemental composition of xLCVO obtained from ICP-OES and XRF analysis.

|  | mass %Li (%) | | | mass %Ca (%) | | | mass %V (%) | | | mass %O (%) | | |
| --- | --- | --- | --- | --- | --- | --- | --- | --- | --- | --- | --- | --- |
|  | TC | ICP-OES | XRF | TC | ICP-OES | XRF | TC | ICP-OES | XRF | TC | ICP-OES | XRF |
| 0LCVO-ABR | 15.44 | 14.96 | - | 0 | 0 | 0 | 37.50 | 37.91 | 36.14 | 47.06 | - | 48.15 |
| 1LCVO-ABR | 15.31 | 14.65 | - | 0.29 | 0.18 | 0.31 | 37.43 | 36.01 | 37.12 | 46.96 | - | 47.01 |
| 3LCVO-ABR | 15.05 | 13.14 | - | 0.88 | 0.76 | 0.93 | 37.27 | 35.02 | 36.21 | 46.79 | - | 46.02 |
| 5LCVO-ABR | 14.78 | 13.65 | - | 1.46 | 1.12 | 1.36 | 37.15 | 37.06 | 36.94 | 46.61 | - | 45.21 |
| 0LCVO-SSR | 15.44 | 13.02 | - | 0 | 0 | 0 | 37.50 | 37.81 | 36.54 | 47.06 | - | 48.42 |
| 3LCVO-SSR | 15.05 | 12.42 | - | 0.29 | 0.25 | 0.30 | 37.43 | 37.21 | 37.51 | 46.96 | - | 44.12 |

**Supplementary Table 6.** DFT-calculated *E*_vac_ of LVO and LCVO.

| sites | *E*_vac_ (eV) | Ave. *E*_vac_ (eV) |
| --- | --- | --- |
| V_O1_ (LVO) | 4.06 | 3.99 |
| V_O2_ (LVO) | 3.95 |  |
| V_O3_ (LVO) | 3.97 |  |
| V_O1_ (LCVO) | 3.33 | 3.50 |
| V_O2_ (LCVO) | 3.65 |  |
| V_O3_ (LCVO) | 3.51 |  |

**Supplementary Table 7.** Crystallite size and microstrain of xLCVO-ABR and –SSR calculated by Williamson – Hall plots.

| samples | Williamson – Hall fitting equation | crystallite size, D (Å) | lattice microstrain, ε |
| --- | --- | --- | --- |
| 0LCVO-ABR | y = 0.00182x + 0.00407 | 378.82 | 0.00182 |
| 3LCVO-ABR | y = 0.00034x + 0.00859 | 179.49 | 0.00034 |
| 0LCVO-SSR | y = 0.00309x + 0.00019 | 8114.74 | 0.00309 |
| 3LCVO-SSR | y = 0.00273x + 0.00069 | 2234.49 | 0.00273 |

**Supplementary Table 8.** DFT calculation result on electron occupation of pure Li_3_VO_4_ (spin component 1; occupation: 1 = filled, 0 = empty).

| band number | band energies (eV) | occupation |
| --- | --- | --- |
| 1 | -69.1771 | 1 |
| 2 | -69.177 | 1 |
| 3 | -69.177 | 1 |
| 4 | -69.177 | 1 |
| 5 | -69.177 | 1 |
| 6 | -69.177 | 1 |
| 7 | -69.177 | 1 |
| 8 | -69.1769 | 1 |
| 9 | -69.1769 | 1 |
| 10 | -69.1769 | 1 |
| 11 | -69.1769 | 1 |
| 12 | -69.1769 | 1 |
| 13 | -69.1769 | 1 |
| 14 | -69.1769 | 1 |
| 15 | -69.1769 | 1 |
| 16 | -69.1769 | 1 |
| 17 | -45.3798 | 1 |
| 18 | -45.3796 | 1 |
| 19 | -45.3795 | 1 |
| 20 | -45.3795 | 1 |
| 21 | -45.3794 | 1 |
| 22 | -45.3794 | 1 |
| 23 | -45.3794 | 1 |
| 24 | -45.3793 | 1 |
| 25 | -45.3793 | 1 |
| 26 | -45.3792 | 1 |
| 27 | -45.3792 | 1 |
| 28 | -45.3791 | 1 |
| 29 | -45.3791 | 1 |
| 30 | -45.3791 | 1 |
| 31 | -45.379 | 1 |
| 32 | -45.3789 | 1 |
| 33 | -45.2271 | 1 |
| 34 | -45.2266 | 1 |
| 35 | -45.2241 | 1 |
| 36 | -45.2241 | 1 |
| 37 | -45.2239 | 1 |
| 38 | -45.2239 | 1 |
| 39 | -45.2237 | 1 |
| 40 | -45.2236 | 1 |
| 41 | -45.2234 | 1 |
| 42 | -45.2232 | 1 |
| 43 | -45.2212 | 1 |
| 44 | -45.221 | 1 |
| 45 | -45.2208 | 1 |
| 46 | -45.2206 | 1 |
| 47 | -45.2205 | 1 |
| 48 | -45.2204 | 1 |
| 49 | -45.2204 | 1 |
| 50 | -45.2203 | 1 |
| 51 | -45.2201 | 1 |
| 52 | -45.2201 | 1 |
| 53 | -45.2195 | 1 |
| 54 | -45.2193 | 1 |
| 55 | -45.2176 | 1 |
| 56 | -45.2175 | 1 |
| 57 | -45.2172 | 1 |
| 58 | -45.2172 | 1 |
| 59 | -45.2165 | 1 |
| 60 | -45.2165 | 1 |
| 61 | -45.2165 | 1 |
| 62 | -45.2165 | 1 |
| 63 | -45.2136 | 1 |
| 64 | -45.2135 | 1 |
| 65 | -41.413 | 1 |
| 66 | -41.4129 | 1 |
| 67 | -41.4128 | 1 |
| 68 | -41.4128 | 1 |
| 69 | -41.4127 | 1 |
| 70 | -41.4127 | 1 |
| 71 | -41.4127 | 1 |
| 72 | -41.4127 | 1 |
| 73 | -41.4127 | 1 |
| 74 | -41.4127 | 1 |
| 75 | -41.4126 | 1 |
| 76 | -41.4126 | 1 |
| 77 | -41.4126 | 1 |
| 78 | -41.4125 | 1 |
| 79 | -41.4123 | 1 |
| 80 | -41.4122 | 1 |
| 81 | -41.3975 | 1 |
| 82 | -41.3974 | 1 |
| 83 | -41.3974 | 1 |
| 84 | -41.3973 | 1 |
| 85 | -41.3973 | 1 |
| 86 | -41.3973 | 1 |
| 87 | -41.3973 | 1 |
| 88 | -41.3973 | 1 |
| 89 | -41.3973 | 1 |
| 90 | -41.3972 | 1 |
| 91 | -41.3972 | 1 |
| 92 | -41.3972 | 1 |
| 93 | -41.3972 | 1 |
| 94 | -41.3971 | 1 |
| 95 | -41.397 | 1 |
| 96 | -41.397 | 1 |
| 97 | -41.3929 | 1 |
| 98 | -41.3929 | 1 |
| 99 | -41.3927 | 1 |
| 100 | -41.3926 | 1 |
| 101 | -41.3926 | 1 |
| 102 | -41.3925 | 1 |
| 103 | -41.3925 | 1 |
| 104 | -41.3925 | 1 |
| 105 | -41.3924 | 1 |
| 106 | -41.3924 | 1 |
| 107 | -41.3923 | 1 |
| 108 | -41.3923 | 1 |
| 109 | -41.3923 | 1 |
| 110 | -41.3922 | 1 |
| 111 | -41.392 | 1 |
| 112 | -41.3919 | 1 |
| 113 | -18.3927 | 1 |
| 114 | -18.2783 | 1 |
| 115 | -18.2447 | 1 |
| 116 | -18.2447 | 1 |
| 117 | -18.1933 | 1 |
| 118 | -18.1932 | 1 |
| 119 | -18.1419 | 1 |
| 120 | -18.1417 | 1 |
| 121 | -18.1345 | 1 |
| 122 | -18.1183 | 1 |
| 123 | -18.1183 | 1 |
| 124 | -18.0942 | 1 |
| 125 | -18.0941 | 1 |
| 126 | -18.0805 | 1 |
| 127 | -18.0804 | 1 |
| 128 | -18.0541 | 1 |
| 129 | -17.7865 | 1 |
| 130 | -17.7861 | 1 |
| 131 | -17.7627 | 1 |
| 132 | -17.7626 | 1 |
| 133 | -17.7608 | 1 |
| 134 | -17.7605 | 1 |
| 135 | -17.7544 | 1 |
| 136 | -17.753 | 1 |
| 137 | -17.7526 | 1 |
| 138 | -17.7402 | 1 |
| 139 | -17.7397 | 1 |
| 140 | -17.7213 | 1 |
| 141 | -17.6984 | 1 |
| 142 | -17.6969 | 1 |
| 143 | -17.693 | 1 |
| 144 | -17.6827 | 1 |
| 145 | -17.6824 | 1 |
| 146 | -17.6822 | 1 |
| 147 | -17.6762 | 1 |
| 148 | -17.676 | 1 |
| 149 | -17.6642 | 1 |
| 150 | -17.6638 | 1 |
| 151 | -17.6633 | 1 |
| 152 | -17.663 | 1 |
| 153 | -17.6567 | 1 |
| 154 | -17.6566 | 1 |
| 155 | -17.6503 | 1 |
| 156 | -17.6502 | 1 |
| 157 | -17.644 | 1 |
| 158 | -17.6439 | 1 |
| 159 | -17.6439 | 1 |
| 160 | -17.6437 | 1 |
| 161 | -17.6416 | 1 |
| 162 | -17.638 | 1 |
| 163 | -17.6371 | 1 |
| 164 | -17.6369 | 1 |
| 165 | -17.6339 | 1 |
| 166 | -17.6338 | 1 |
| 167 | -17.6272 | 1 |
| 168 | -17.6271 | 1 |
| 169 | -17.6017 | 1 |
| 170 | -17.6016 | 1 |
| 171 | -17.5838 | 1 |
| 172 | -17.5815 | 1 |
| 173 | -17.5798 | 1 |
| 174 | -17.5743 | 1 |
| 175 | -17.5611 | 1 |
| 176 | -17.5611 | 1 |
| 177 | -4.0657 | 1 |
| 178 | -4.0654 | 1 |
| 179 | -4.0195 | 1 |
| 180 | -4.0192 | 1 |
| 181 | -4.0168 | 1 |
| 182 | -4.0168 | 1 |
| 183 | -3.9976 | 1 |
| 184 | -3.9973 | 1 |
| 185 | -3.9382 | 1 |
| 186 | -3.9379 | 1 |
| 187 | -3.8848 | 1 |
| 188 | -3.8845 | 1 |
| 189 | -3.874 | 1 |
| 190 | -3.851 | 1 |
| 191 | -3.8337 | 1 |
| 192 | -3.8335 | 1 |
| 193 | -3.8242 | 1 |
| 194 | -3.8239 | 1 |
| 195 | -3.8238 | 1 |
| 196 | -3.7764 | 1 |
| 197 | -3.7578 | 1 |
| 198 | -3.7575 | 1 |
| 199 | -3.7523 | 1 |
| 200 | -3.735 | 1 |
| 201 | -3.6917 | 1 |
| 202 | -3.6915 | 1 |
| 203 | -3.6558 | 1 |
| 204 | -3.6556 | 1 |
| 205 | -3.6417 | 1 |
| 206 | -3.6416 | 1 |
| 207 | -3.6288 | 1 |
| 208 | -3.6287 | 1 |
| 209 | -3.5762 | 1 |
| 210 | -3.5762 | 1 |
| 211 | -3.5393 | 1 |
| 212 | -3.4976 | 1 |
| 213 | -3.4976 | 1 |
| 214 | -3.4875 | 1 |
| 215 | -3.4813 | 1 |
| 216 | -3.4812 | 1 |
| 217 | -3.4794 | 1 |
| 218 | -3.4793 | 1 |
| 219 | -3.4605 | 1 |
| 220 | -3.4521 | 1 |
| 221 | -3.4371 | 1 |
| 222 | -3.3654 | 1 |
| 223 | -3.3653 | 1 |
| 224 | -3.2203 | 1 |
| 225 | -3.2202 | 1 |
| 226 | -3.2106 | 1 |
| 227 | -3.2105 | 1 |
| 228 | -3.1938 | 1 |
| 229 | -3.1937 | 1 |
| 230 | -3.1892 | 1 |
| 231 | -3.1889 | 1 |
| 232 | -3.1749 | 1 |
| 233 | -3.1747 | 1 |
| 234 | -3.1675 | 1 |
| 235 | -3.1674 | 1 |
| 236 | -3.1625 | 1 |
| 237 | -3.1528 | 1 |
| 238 | -3.1527 | 1 |
| 239 | -3.0888 | 1 |
| 240 | -3.0747 | 1 |
| 241 | -3.0747 | 1 |
| 242 | -3.0544 | 1 |
| 243 | -3.0459 | 1 |
| 244 | -3.022 | 1 |
| 245 | -2.9643 | 1 |
| 246 | -2.964 | 1 |
| 247 | -2.9526 | 1 |
| 248 | -2.9525 | 1 |
| 249 | -2.9494 | 1 |
| 250 | -2.9449 | 1 |
| 251 | -2.944 | 1 |
| 252 | -2.9254 | 1 |
| 253 | -2.9103 | 1 |
| 254 | -2.9101 | 1 |
| 255 | -2.9053 | 1 |
| 256 | -2.9052 | 1 |
| 257 | -2.8932 | 1 |
| 258 | -2.8929 | 1 |
| 259 | -2.8862 | 1 |
| 260 | -2.7948 | 1 |
| 261 | -2.7948 | 1 |
| 262 | -2.793 | 1 |
| 263 | -2.7928 | 1 |
| 264 | -2.7843 | 1 |
| 265 | -2.7841 | 1 |
| 266 | -2.7771 | 1 |
| 267 | -2.734 | 1 |
| 268 | -2.7339 | 1 |
| 269 | -2.6412 | 1 |
| 270 | -2.641 | 1 |
| 271 | -2.5968 | 1 |
| 272 | -2.5232 | 1 |
| 273 | -2.487 | 1 |
| 274 | -2.4868 | 1 |
| 275 | -2.4552 | 1 |
| 276 | -2.3667 | 1 |
| 277 | -2.3378 | 1 |
| 278 | -2.3351 | 1 |
| 279 | -2.3278 | 1 |
| 280 | -2.2757 | 1 |
| 281 | -2.2757 | 1 |
| 282 | -2.2576 | 1 |
| 283 | -2.2439 | 1 |
| 284 | -2.2439 | 1 |
| 285 | -2.2218 | 1 |
| 286 | -2.2217 | 1 |
| 287 | -2.1578 | 1 |
| 288 | -2.1578 | 1 |
| 289 | -2.0981 | 1 |
| 290 | -2.0981 | 1 |
| 291 | -2.084 | 1 |
| 292 | -2.0839 | 1 |
| 293 | -2.0304 | 1 |
| 294 | -2.0303 | 1 |
| 295 | -2.029 | 1 |
| 296 | -2.023 | 1 |
| 297 | -2.023 | 1 |
| 298 | -2.0036 | 1 |
| 299 | -1.9641 | 1 |
| 300 | -1.893 | 1 |
| 301 | -1.893 | 1 |
| 302 | -1.8395 | 1 |
| 303 | -1.8395 | 1 |
| 304 | -1.8142 | 1 |
| 305 | -1.8142 | 1 |
| 306 | -1.7357 | 1 |
| 307 | -1.7356 | 1 |
| 308 | -1.7243 | 1 |
| 309 | -1.7243 | 1 |
| 310 | -1.6831 | 1 |
| 311 | -1.6376 | 1 |
| 312 | -1.58 | 1 |
| 313 | -1.5799 | 1 |
| 314 | -1.5526 | 1 |
| 315 | -1.5525 | 1 |
| 316 | -1.5117 | 1 |
| 317 | -1.5117 | 1 |
| 318 | -1.5088 | 1 |
| 319 | -1.5084 | 1 |
| 320 | -1.5083 | 1 |
| 321 | -1.4473 | 1 |
| 322 | -1.4473 | 1 |
| 323 | -1.4307 | 1 |
| 324 | -1.3537 | 1 |
| 325 | -1.3474 | 1 |
| 326 | -1.2803 | 1 |
| 327 | -1.2525 | 1 |
| 328 | -1.2525 | 1 |
| 329 | -1.2171 | 1 |
| 330 | -1.1925 | 1 |
| 331 | -1.1922 | 1 |
| 332 | -1.1571 | 1 |
| 333 | -1.1469 | 1 |
| 334 | -1.1232 | 1 |
| 335 | -1.1232 | 1 |
| 336 | -1.1184 | 1 |
| 337 | -1.1182 | 1 |
| 338 | -1.1165 | 1 |
| 339 | -1.1162 | 1 |
| 340 | -1.1131 | 1 |
| 341 | -1.0809 | 1 |
| 342 | -1.0807 | 1 |
| 343 | -1.0461 | 1 |
| 344 | -1.0459 | 1 |
| 345 | -1.0062 | 1 |
| 346 | -1.0062 | 1 |
| 347 | -0.9993 | 1 |
| 348 | -0.9889 | 1 |
| 349 | -0.9888 | 1 |
| 350 | -0.9767 | 1 |
| 351 | -0.9321 | 1 |
| 352 | -0.9321 | 1 |
| 353 | -0.9293 | 1 |
| 354 | -0.9291 | 1 |
| 355 | -0.9235 | 1 |
| 356 | -0.9233 | 1 |
| 357 | -0.9164 | 1 |
| 358 | -0.9163 | 1 |
| 359 | -0.8621 | 1 |
| 360 | -0.862 | 1 |
| 361 | -0.8457 | 1 |
| 362 | -0.8457 | 1 |
| 363 | -0.8404 | 1 |
| 364 | -0.7788 | 1 |
| 365 | -0.7787 | 1 |
| 366 | -0.7682 | 1 |
| 367 | -0.7681 | 1 |
| **368** | **-0.7007** | **1** |
| **369** | **5.1444** | **0** |
| 370 | 5.1444 | 0 |
| 371 | 5.1599 | 0 |
| 372 | 5.1599 | 0 |
| 373 | 5.1739 | 0 |
| 374 | 5.1739 | 0 |
| 375 | 5.196 | 0 |
| 376 | 5.196 | 0 |
| 377 | 5.2132 | 0 |
| 378 | 5.2132 | 0 |
| 379 | 5.2134 | 0 |
| 380 | 5.2163 | 0 |
| 381 | 5.2164 | 0 |
| 382 | 5.2349 | 0 |
| 383 | 5.2565 | 0 |
| 384 | 5.263 | 0 |
| 385 | 5.263 | 0 |
| 386 | 5.2791 | 0 |
| 387 | 5.2791 | 0 |
| 388 | 5.2798 | 0 |
| 389 | 5.2798 | 0 |
| 390 | 5.2817 | 0 |
| 391 | 5.2817 | 0 |
| 392 | 5.2987 | 0 |
| 393 | 5.304 | 0 |
| 394 | 5.3127 | 0 |
| 395 | 5.3127 | 0 |
| 396 | 5.3178 | 0 |
| 397 | 5.3204 | 0 |
| 398 | 5.3598 | 0 |
| 399 | 5.3598 | 0 |
| 400 | 5.371 | 0 |
| 401 | 5.5386 | 0 |
| 402 | 5.6045 | 0 |
| 403 | 5.7847 | 0 |
| 404 | 5.7851 | 0 |
| 405 | 5.7982 | 0 |
| 406 | 5.7982 | 0 |
| 407 | 5.8052 | 0 |
| 408 | 5.8052 | 0 |
| 409 | 5.8326 | 0 |
| 410 | 5.8329 | 0 |
| 411 | 5.895 | 0 |
| 412 | 5.9115 | 0 |
| 413 | 5.9116 | 0 |
| 414 | 5.9298 | 0 |
| 415 | 5.9375 | 0 |
| 416 | 5.9375 | 0 |
| 417 | 6.0051 | 0 |
| 418 | 6.0256 | 0 |
| 419 | 6.0257 | 0 |
| 420 | 6.0334 | 0 |
| 421 | 6.0334 | 0 |
| 422 | 6.039 | 0 |
| 423 | 6.1109 | 0 |
| 424 | 6.1109 | 0 |
| 425 | 6.1157 | 0 |
| 426 | 6.1157 | 0 |
| 427 | 6.1267 | 0 |
| 428 | 6.1268 | 0 |
| 429 | 6.1378 | 0 |
| 430 | 6.1568 | 0 |
| 431 | 6.1568 | 0 |
| 432 | 6.1772 | 0 |
| 433 | 6.1927 | 0 |
| 434 | 6.1928 | 0 |
| 435 | 6.2047 | 0 |
| 436 | 6.2072 | 0 |
| 437 | 6.2171 | 0 |
| 438 | 6.2172 | 0 |
| 439 | 6.3117 | 0 |
| 440 | 6.3409 | 0 |
| 441 | 6.384 | 0 |
| 442 | 6.3841 | 0 |
| 443 | 6.4901 | 0 |
| 444 | 6.4902 | 0 |
| 445 | 6.5058 | 0 |
| 446 | 6.5059 | 0 |
| 447 | 6.5262 | 0 |
| 448 | 6.5262 | 0 |

**Supplementary Table 9.** DFT calculation result on electron occupation of pure Li_3_VO_4_ (spin component 2; occupation: 1 = filled, 0 = empty).

| band number | band energies (eV) | occupation |
| --- | --- | --- |
| 1 | -69.1771 | 1 |
| 2 | -69.177 | 1 |
| 3 | -69.177 | 1 |
| 4 | -69.177 | 1 |
| 5 | -69.177 | 1 |
| 6 | -69.177 | 1 |
| 7 | -69.177 | 1 |
| 8 | -69.1769 | 1 |
| 9 | -69.1769 | 1 |
| 10 | -69.1769 | 1 |
| 11 | -69.1769 | 1 |
| 12 | -69.1769 | 1 |
| 13 | -69.1769 | 1 |
| 14 | -69.1769 | 1 |
| 15 | -69.1769 | 1 |
| 16 | -69.1769 | 1 |
| 17 | -45.3798 | 1 |
| 18 | -45.3796 | 1 |
| 19 | -45.3795 | 1 |
| 20 | -45.3795 | 1 |
| 21 | -45.3794 | 1 |
| 22 | -45.3794 | 1 |
| 23 | -45.3794 | 1 |
| 24 | -45.3793 | 1 |
| 25 | -45.3793 | 1 |
| 26 | -45.3792 | 1 |
| 27 | -45.3792 | 1 |
| 28 | -45.3791 | 1 |
| 29 | -45.3791 | 1 |
| 30 | -45.3791 | 1 |
| 31 | -45.379 | 1 |
| 32 | -45.3789 | 1 |
| 33 | -45.2271 | 1 |
| 34 | -45.2266 | 1 |
| 35 | -45.2241 | 1 |
| 36 | -45.2241 | 1 |
| 37 | -45.2239 | 1 |
| 38 | -45.2239 | 1 |
| 39 | -45.2237 | 1 |
| 40 | -45.2236 | 1 |
| 41 | -45.2234 | 1 |
| 42 | -45.2232 | 1 |
| 43 | -45.2212 | 1 |
| 44 | -45.221 | 1 |
| 45 | -45.2208 | 1 |
| 46 | -45.2206 | 1 |
| 47 | -45.2205 | 1 |
| 48 | -45.2204 | 1 |
| 49 | -45.2204 | 1 |
| 50 | -45.2203 | 1 |
| 51 | -45.2201 | 1 |
| 52 | -45.2201 | 1 |
| 53 | -45.2195 | 1 |
| 54 | -45.2193 | 1 |
| 55 | -45.2176 | 1 |
| 56 | -45.2175 | 1 |
| 57 | -45.2172 | 1 |
| 58 | -45.2172 | 1 |
| 59 | -45.2165 | 1 |
| 60 | -45.2165 | 1 |
| 61 | -45.2165 | 1 |
| 62 | -45.2165 | 1 |
| 63 | -45.2136 | 1 |
| 64 | -45.2135 | 1 |
| 65 | -41.413 | 1 |
| 66 | -41.4129 | 1 |
| 67 | -41.4128 | 1 |
| 68 | -41.4128 | 1 |
| 69 | -41.4127 | 1 |
| 70 | -41.4127 | 1 |
| 71 | -41.4127 | 1 |
| 72 | -41.4127 | 1 |
| 73 | -41.4127 | 1 |
| 74 | -41.4127 | 1 |
| 75 | -41.4126 | 1 |
| 76 | -41.4126 | 1 |
| 77 | -41.4126 | 1 |
| 78 | -41.4125 | 1 |
| 79 | -41.4123 | 1 |
| 80 | -41.4122 | 1 |
| 81 | -41.3975 | 1 |
| 82 | -41.3974 | 1 |
| 83 | -41.3974 | 1 |
| 84 | -41.3973 | 1 |
| 85 | -41.3973 | 1 |
| 86 | -41.3973 | 1 |
| 87 | -41.3973 | 1 |
| 88 | -41.3973 | 1 |
| 89 | -41.3973 | 1 |
| 90 | -41.3972 | 1 |
| 91 | -41.3972 | 1 |
| 92 | -41.3972 | 1 |
| 93 | -41.3972 | 1 |
| 94 | -41.3971 | 1 |
| 95 | -41.397 | 1 |
| 96 | -41.397 | 1 |
| 97 | -41.3929 | 1 |
| 98 | -41.3929 | 1 |
| 99 | -41.3927 | 1 |
| 100 | -41.3926 | 1 |
| 101 | -41.3926 | 1 |
| 102 | -41.3925 | 1 |
| 103 | -41.3925 | 1 |
| 104 | -41.3925 | 1 |
| 105 | -41.3924 | 1 |
| 106 | -41.3924 | 1 |
| 107 | -41.3923 | 1 |
| 108 | -41.3923 | 1 |
| 109 | -41.3923 | 1 |
| 110 | -41.3922 | 1 |
| 111 | -41.392 | 1 |
| 112 | -41.3919 | 1 |
| 113 | -18.3927 | 1 |
| 114 | -18.2783 | 1 |
| 115 | -18.2447 | 1 |
| 116 | -18.2447 | 1 |
| 117 | -18.1933 | 1 |
| 118 | -18.1932 | 1 |
| 119 | -18.1419 | 1 |
| 120 | -18.1417 | 1 |
| 121 | -18.1345 | 1 |
| 122 | -18.1183 | 1 |
| 123 | -18.1183 | 1 |
| 124 | -18.0942 | 1 |
| 125 | -18.0941 | 1 |
| 126 | -18.0805 | 1 |
| 127 | -18.0804 | 1 |
| 128 | -18.0541 | 1 |
| 129 | -17.7865 | 1 |
| 130 | -17.7861 | 1 |
| 131 | -17.7627 | 1 |
| 132 | -17.7626 | 1 |
| 133 | -17.7608 | 1 |
| 134 | -17.7605 | 1 |
| 135 | -17.7544 | 1 |
| 136 | -17.753 | 1 |
| 137 | -17.7526 | 1 |
| 138 | -17.7402 | 1 |
| 139 | -17.7397 | 1 |
| 140 | -17.7213 | 1 |
| 141 | -17.6984 | 1 |
| 142 | -17.6969 | 1 |
| 143 | -17.693 | 1 |
| 144 | -17.6827 | 1 |
| 145 | -17.6824 | 1 |
| 146 | -17.6822 | 1 |
| 147 | -17.6762 | 1 |
| 148 | -17.676 | 1 |
| 149 | -17.6642 | 1 |
| 150 | -17.6638 | 1 |
| 151 | -17.6633 | 1 |
| 152 | -17.663 | 1 |
| 153 | -17.6567 | 1 |
| 154 | -17.6566 | 1 |
| 155 | -17.6503 | 1 |
| 156 | -17.6502 | 1 |
| 157 | -17.644 | 1 |
| 158 | -17.6439 | 1 |
| 159 | -17.6439 | 1 |
| 160 | -17.6437 | 1 |
| 161 | -17.6416 | 1 |
| 162 | -17.638 | 1 |
| 163 | -17.6371 | 1 |
| 164 | -17.6369 | 1 |
| 165 | -17.6339 | 1 |
| 166 | -17.6338 | 1 |
| 167 | -17.6272 | 1 |
| 168 | -17.6271 | 1 |
| 169 | -17.6017 | 1 |
| 170 | -17.6016 | 1 |
| 171 | -17.5838 | 1 |
| 172 | -17.5815 | 1 |
| 173 | -17.5798 | 1 |
| 174 | -17.5743 | 1 |
| 175 | -17.5611 | 1 |
| 176 | -17.5611 | 1 |
| 177 | -4.0657 | 1 |
| 178 | -4.0654 | 1 |
| 179 | -4.0195 | 1 |
| 180 | -4.0192 | 1 |
| 181 | -4.0168 | 1 |
| 182 | -4.0168 | 1 |
| 183 | -3.9976 | 1 |
| 184 | -3.9973 | 1 |
| 185 | -3.9382 | 1 |
| 186 | -3.9379 | 1 |
| 187 | -3.8848 | 1 |
| 188 | -3.8845 | 1 |
| 189 | -3.874 | 1 |
| 190 | -3.851 | 1 |
| 191 | -3.8337 | 1 |
| 192 | -3.8335 | 1 |
| 193 | -3.8242 | 1 |
| 194 | -3.8239 | 1 |
| 195 | -3.8238 | 1 |
| 196 | -3.7764 | 1 |
| 197 | -3.7578 | 1 |
| 198 | -3.7575 | 1 |
| 199 | -3.7523 | 1 |
| 200 | -3.735 | 1 |
| 201 | -3.6917 | 1 |
| 202 | -3.6915 | 1 |
| 203 | -3.6558 | 1 |
| 204 | -3.6556 | 1 |
| 205 | -3.6417 | 1 |
| 206 | -3.6416 | 1 |
| 207 | -3.6288 | 1 |
| 208 | -3.6287 | 1 |
| 209 | -3.5762 | 1 |
| 210 | -3.5762 | 1 |
| 211 | -3.5393 | 1 |
| 212 | -3.4976 | 1 |
| 213 | -3.4976 | 1 |
| 214 | -3.4875 | 1 |
| 215 | -3.4813 | 1 |
| 216 | -3.4812 | 1 |
| 217 | -3.4794 | 1 |
| 218 | -3.4793 | 1 |
| 219 | -3.4605 | 1 |
| 220 | -3.4521 | 1 |
| 221 | -3.4371 | 1 |
| 222 | -3.3654 | 1 |
| 223 | -3.3653 | 1 |
| 224 | -3.2203 | 1 |
| 225 | -3.2202 | 1 |
| 226 | -3.2106 | 1 |
| 227 | -3.2105 | 1 |
| 228 | -3.1938 | 1 |
| 229 | -3.1937 | 1 |
| 230 | -3.1892 | 1 |
| 231 | -3.1889 | 1 |
| 232 | -3.1749 | 1 |
| 233 | -3.1747 | 1 |
| 234 | -3.1675 | 1 |
| 235 | -3.1674 | 1 |
| 236 | -3.1625 | 1 |
| 237 | -3.1528 | 1 |
| 238 | -3.1527 | 1 |
| 239 | -3.0888 | 1 |
| 240 | -3.0747 | 1 |
| 241 | -3.0747 | 1 |
| 242 | -3.0544 | 1 |
| 243 | -3.0459 | 1 |
| 244 | -3.022 | 1 |
| 245 | -2.9643 | 1 |
| 246 | -2.964 | 1 |
| 247 | -2.9526 | 1 |
| 248 | -2.9525 | 1 |
| 249 | -2.9494 | 1 |
| 250 | -2.9449 | 1 |
| 251 | -2.944 | 1 |
| 252 | -2.9254 | 1 |
| 253 | -2.9103 | 1 |
| 254 | -2.9101 | 1 |
| 255 | -2.9053 | 1 |
| 256 | -2.9052 | 1 |
| 257 | -2.8932 | 1 |
| 258 | -2.8929 | 1 |
| 259 | -2.8862 | 1 |
| 260 | -2.7948 | 1 |
| 261 | -2.7948 | 1 |
| 262 | -2.793 | 1 |
| 263 | -2.7928 | 1 |
| 264 | -2.7843 | 1 |
| 265 | -2.7841 | 1 |
| 266 | -2.7771 | 1 |
| 267 | -2.734 | 1 |
| 268 | -2.7339 | 1 |
| 269 | -2.6412 | 1 |
| 270 | -2.641 | 1 |
| 271 | -2.5968 | 1 |
| 272 | -2.5232 | 1 |
| 273 | -2.487 | 1 |
| 274 | -2.4868 | 1 |
| 275 | -2.4552 | 1 |
| 276 | -2.3667 | 1 |
| 277 | -2.3378 | 1 |
| 278 | -2.3351 | 1 |
| 279 | -2.3278 | 1 |
| 280 | -2.2757 | 1 |
| 281 | -2.2757 | 1 |
| 282 | -2.2576 | 1 |
| 283 | -2.2439 | 1 |
| 284 | -2.2439 | 1 |
| 285 | -2.2218 | 1 |
| 286 | -2.2217 | 1 |
| 287 | -2.1578 | 1 |
| 288 | -2.1578 | 1 |
| 289 | -2.0981 | 1 |
| 290 | -2.0981 | 1 |
| 291 | -2.084 | 1 |
| 292 | -2.0839 | 1 |
| 293 | -2.0304 | 1 |
| 294 | -2.0303 | 1 |
| 295 | -2.029 | 1 |
| 296 | -2.023 | 1 |
| 297 | -2.023 | 1 |
| 298 | -2.0036 | 1 |
| 299 | -1.9641 | 1 |
| 300 | -1.893 | 1 |
| 301 | -1.893 | 1 |
| 302 | -1.8395 | 1 |
| 303 | -1.8395 | 1 |
| 304 | -1.8142 | 1 |
| 305 | -1.8142 | 1 |
| 306 | -1.7357 | 1 |
| 307 | -1.7356 | 1 |
| 308 | -1.7243 | 1 |
| 309 | -1.7243 | 1 |
| 310 | -1.6831 | 1 |
| 311 | -1.6376 | 1 |
| 312 | -1.58 | 1 |
| 313 | -1.5799 | 1 |
| 314 | -1.5526 | 1 |
| 315 | -1.5525 | 1 |
| 316 | -1.5117 | 1 |
| 317 | -1.5117 | 1 |
| 318 | -1.5088 | 1 |
| 319 | -1.5084 | 1 |
| 320 | -1.5083 | 1 |
| 321 | -1.4473 | 1 |
| 322 | -1.4473 | 1 |
| 323 | -1.4307 | 1 |
| 324 | -1.3537 | 1 |
| 325 | -1.3474 | 1 |
| 326 | -1.2803 | 1 |
| 327 | -1.2525 | 1 |
| 328 | -1.2525 | 1 |
| 329 | -1.2171 | 1 |
| 330 | -1.1925 | 1 |
| 331 | -1.1922 | 1 |
| 332 | -1.1571 | 1 |
| 333 | -1.1469 | 1 |
| 334 | -1.1232 | 1 |
| 335 | -1.1232 | 1 |
| 336 | -1.1184 | 1 |
| 337 | -1.1182 | 1 |
| 338 | -1.1165 | 1 |
| 339 | -1.1162 | 1 |
| 340 | -1.1131 | 1 |
| 341 | -1.0809 | 1 |
| 342 | -1.0807 | 1 |
| 343 | -1.0461 | 1 |
| 344 | -1.0459 | 1 |
| 345 | -1.0062 | 1 |
| 346 | -1.0062 | 1 |
| 347 | -0.9993 | 1 |
| 348 | -0.9889 | 1 |
| 349 | -0.9888 | 1 |
| 350 | -0.9767 | 1 |
| 351 | -0.9321 | 1 |
| 352 | -0.9321 | 1 |
| 353 | -0.9293 | 1 |
| 354 | -0.9291 | 1 |
| 355 | -0.9235 | 1 |
| 356 | -0.9233 | 1 |
| 357 | -0.9164 | 1 |
| 358 | -0.9163 | 1 |
| 359 | -0.8621 | 1 |
| 360 | -0.862 | 1 |
| 361 | -0.8457 | 1 |
| 362 | -0.8457 | 1 |
| 363 | -0.8404 | 1 |
| 364 | -0.7788 | 1 |
| 365 | -0.7787 | 1 |
| 366 | -0.7682 | 1 |
| 367 | -0.7681 | 1 |
| **368** | **-0.7007** | **1** |
| **369** | **5.1444** | **0** |
| 370 | 5.1444 | 0 |
| 371 | 5.1599 | 0 |
| 372 | 5.1599 | 0 |
| 373 | 5.1739 | 0 |
| 374 | 5.1739 | 0 |
| 375 | 5.196 | 0 |
| 376 | 5.196 | 0 |
| 377 | 5.2132 | 0 |
| 378 | 5.2132 | 0 |
| 379 | 5.2134 | 0 |
| 380 | 5.2163 | 0 |
| 381 | 5.2164 | 0 |
| 382 | 5.2349 | 0 |
| 383 | 5.2565 | 0 |
| 384 | 5.263 | 0 |
| 385 | 5.263 | 0 |
| 386 | 5.2791 | 0 |
| 387 | 5.2791 | 0 |
| 388 | 5.2798 | 0 |
| 389 | 5.2798 | 0 |
| 390 | 5.2817 | 0 |
| 391 | 5.2817 | 0 |
| 392 | 5.2987 | 0 |
| 393 | 5.304 | 0 |
| 394 | 5.3127 | 0 |
| 395 | 5.3127 | 0 |
| 396 | 5.3178 | 0 |
| 397 | 5.3204 | 0 |
| 398 | 5.3598 | 0 |
| 399 | 5.3598 | 0 |
| 400 | 5.371 | 0 |
| 401 | 5.5386 | 0 |
| 402 | 5.6045 | 0 |
| 403 | 5.7847 | 0 |
| 404 | 5.7851 | 0 |
| 405 | 5.7982 | 0 |
| 406 | 5.7982 | 0 |
| 407 | 5.8052 | 0 |
| 408 | 5.8052 | 0 |
| 409 | 5.8326 | 0 |
| 410 | 5.8329 | 0 |
| 411 | 5.895 | 0 |
| 412 | 5.9115 | 0 |
| 413 | 5.9116 | 0 |
| 414 | 5.9298 | 0 |
| 415 | 5.9375 | 0 |
| 416 | 5.9375 | 0 |
| 417 | 6.0051 | 0 |
| 418 | 6.0256 | 0 |
| 419 | 6.0257 | 0 |
| 420 | 6.0334 | 0 |
| 421 | 6.0334 | 0 |
| 422 | 6.039 | 0 |
| 423 | 6.1109 | 0 |
| 424 | 6.1109 | 0 |
| 425 | 6.1157 | 0 |
| 426 | 6.1157 | 0 |
| 427 | 6.1267 | 0 |
| 428 | 6.1268 | 0 |
| 429 | 6.1378 | 0 |
| 430 | 6.1568 | 0 |
| 431 | 6.1568 | 0 |
| 432 | 6.1772 | 0 |
| 433 | 6.1927 | 0 |
| 434 | 6.1928 | 0 |
| 435 | 6.2047 | 0 |
| 436 | 6.2072 | 0 |
| 437 | 6.2171 | 0 |
| 438 | 6.2172 | 0 |
| 439 | 6.3117 | 0 |
| 440 | 6.3409 | 0 |
| 441 | 6.384 | 0 |
| 442 | 6.3841 | 0 |
| 443 | 6.4901 | 0 |
| 444 | 6.4902 | 0 |
| 445 | 6.5058 | 0 |
| 446 | 6.5059 | 0 |
| 447 | 6.5262 | 0 |
| 448 | 6.5262 | 0 |

**Supplementary Table 10.** DFT calculation result on electron occupation of Ca-doped Li_3_VO_4_ (spin component 1; occupation: 1 = filled, 0 = empty).

| band number | band energies (eV) | occupation |
| --- | --- | --- |
| 1 | -69.5185 | 1 |
| 2 | -69.4049 | 1 |
| 3 | -69.3344 | 1 |
| 4 | -69.3193 | 1 |
| 5 | -69.0404 | 1 |
| 6 | -69.0401 | 1 |
| 7 | -69.0082 | 1 |
| 8 | -69.0063 | 1 |
| 9 | -68.9285 | 1 |
| 10 | -68.9278 | 1 |
| 11 | -68.9093 | 1 |
| 12 | -68.8956 | 1 |
| 13 | -68.8951 | 1 |
| 14 | -68.8807 | 1 |
| 15 | -66.946 | 1 |
| 16 | -66.9298 | 1 |
| 17 | -45.7292 | 1 |
| 18 | -45.7155 | 1 |
| 19 | -45.3454 | 1 |
| 20 | -45.3336 | 1 |
| 21 | -45.289 | 1 |
| 22 | -45.2882 | 1 |
| 23 | -45.2857 | 1 |
| 24 | -45.2821 | 1 |
| 25 | -45.2672 | 1 |
| 26 | -45.2638 | 1 |
| 27 | -45.2635 | 1 |
| 28 | -45.262 | 1 |
| 29 | -45.2469 | 1 |
| 30 | -45.244 | 1 |
| 31 | -45.2344 | 1 |
| 32 | -45.2312 | 1 |
| 33 | -45.2228 | 1 |
| 34 | -45.2201 | 1 |
| 35 | -45.2081 | 1 |
| 36 | -45.2072 | 1 |
| 37 | -45.2049 | 1 |
| 38 | -45.2029 | 1 |
| 39 | -45.1632 | 1 |
| 40 | -45.1569 | 1 |
| 41 | -45.1173 | 1 |
| 42 | -45.1135 | 1 |
| 43 | -45.0915 | 1 |
| 44 | -45.0914 | 1 |
| 45 | -45.0885 | 1 |
| 46 | -45.0885 | 1 |
| 47 | -45.0855 | 1 |
| 48 | -45.0822 | 1 |
| 49 | -45.0605 | 1 |
| 50 | -45.0446 | 1 |
| 51 | -44.9472 | 1 |
| 52 | -44.9438 | 1 |
| 53 | -44.9403 | 1 |
| 54 | -44.9366 | 1 |
| 55 | -44.9232 | 1 |
| 56 | -44.9157 | 1 |
| 57 | -44.911 | 1 |
| 58 | -44.9077 | 1 |
| 59 | -44.6432 | 1 |
| 60 | -44.637 | 1 |
| 61 | -44.6254 | 1 |
| 62 | -44.6205 | 1 |
| 63 | -41.7605 | 1 |
| 64 | -41.7252 | 1 |
| 65 | -41.7122 | 1 |
| 66 | -41.651 | 1 |
| 67 | -41.6199 | 1 |
| 68 | -41.6062 | 1 |
| 69 | -41.5883 | 1 |
| 70 | -41.5731 | 1 |
| 71 | -41.5495 | 1 |
| 72 | -41.546 | 1 |
| 73 | -41.5338 | 1 |
| 74 | -41.5337 | 1 |
| 75 | -41.2922 | 1 |
| 76 | -41.292 | 1 |
| 77 | -41.2779 | 1 |
| 78 | -41.2775 | 1 |
| 79 | -41.2769 | 1 |
| 80 | -41.2766 | 1 |
| 81 | -41.2658 | 1 |
| 82 | -41.2636 | 1 |
| 83 | -41.2494 | 1 |
| 84 | -41.2475 | 1 |
| 85 | -41.2428 | 1 |
| 86 | -41.241 | 1 |
| 87 | -41.1845 | 1 |
| 88 | -41.1838 | 1 |
| 89 | -41.1793 | 1 |
| 90 | -41.1788 | 1 |
| 91 | -41.1749 | 1 |
| 92 | -41.1742 | 1 |
| 93 | -41.1709 | 1 |
| 94 | -41.1491 | 1 |
| 95 | -41.1441 | 1 |
| 96 | -41.1424 | 1 |
| 97 | -41.1406 | 1 |
| 98 | -41.1352 | 1 |
| 99 | -41.1349 | 1 |
| 100 | -41.1326 | 1 |
| 101 | -41.1281 | 1 |
| 102 | -41.1269 | 1 |
| 103 | -41.1218 | 1 |
| 104 | -41.1053 | 1 |
| 105 | -39.706 | 1 |
| 106 | -39.6958 | 1 |
| 107 | -39.3599 | 1 |
| 108 | -39.3464 | 1 |
| 109 | -39.3247 | 1 |
| 110 | -39.2816 | 1 |
| 111 | -39.245 | 1 |
| 112 | -39.2368 | 1 |
| 113 | -19.9389 | 1 |
| 114 | -19.9168 | 1 |
| 115 | -19.7851 | 1 |
| 116 | -19.7826 | 1 |
| 117 | -19.7755 | 1 |
| 118 | -19.7388 | 1 |
| 119 | -18.4313 | 1 |
| 120 | -18.373 | 1 |
| 121 | -18.2289 | 1 |
| 122 | -18.2145 | 1 |
| 123 | -18.1868 | 1 |
| 124 | -18.1819 | 1 |
| 125 | -18.1176 | 1 |
| 126 | -18.0987 | 1 |
| 127 | -18.0677 | 1 |
| 128 | -18.0489 | 1 |
| 129 | -18.0476 | 1 |
| 130 | -17.9707 | 1 |
| 131 | -17.9476 | 1 |
| 132 | -17.9303 | 1 |
| 133 | -17.9267 | 1 |
| 134 | -17.9049 | 1 |
| 135 | -17.8953 | 1 |
| 136 | -17.8841 | 1 |
| 137 | -17.8393 | 1 |
| 138 | -17.8245 | 1 |
| 139 | -17.7893 | 1 |
| 140 | -17.7839 | 1 |
| 141 | -17.7642 | 1 |
| 142 | -17.7502 | 1 |
| 143 | -17.6924 | 1 |
| 144 | -17.677 | 1 |
| 145 | -17.6606 | 1 |
| 146 | -17.657 | 1 |
| 147 | -17.6372 | 1 |
| 148 | -17.6279 | 1 |
| 149 | -17.6242 | 1 |
| 150 | -17.6189 | 1 |
| 151 | -17.6166 | 1 |
| 152 | -17.6073 | 1 |
| 153 | -17.5959 | 1 |
| 154 | -17.5947 | 1 |
| 155 | -17.5917 | 1 |
| 156 | -17.5806 | 1 |
| 157 | -17.5783 | 1 |
| 158 | -17.5764 | 1 |
| 159 | -17.5624 | 1 |
| 160 | -17.5601 | 1 |
| 161 | -17.5556 | 1 |
| 162 | -17.5488 | 1 |
| 163 | -17.5397 | 1 |
| 164 | -17.5306 | 1 |
| 165 | -17.4952 | 1 |
| 166 | -17.4944 | 1 |
| 167 | -17.4706 | 1 |
| 168 | -17.4555 | 1 |
| 169 | -17.4429 | 1 |
| 170 | -17.4365 | 1 |
| 171 | -17.4358 | 1 |
| 172 | -17.4292 | 1 |
| 173 | -17.4211 | 1 |
| 174 | -17.4037 | 1 |
| 175 | -16.805 | 1 |
| 176 | -16.7041 | 1 |
| 177 | -16.3927 | 1 |
| 178 | -16.3653 | 1 |
| 179 | -16.3253 | 1 |
| 180 | -16.2921 | 1 |
| 181 | -16.1348 | 1 |
| 182 | -16.0286 | 1 |
| 183 | -4.1911 | 1 |
| 184 | -4.1594 | 1 |
| 185 | -4.0496 | 1 |
| 186 | -4.0294 | 1 |
| 187 | -4.024 | 1 |
| 188 | -3.9898 | 1 |
| 189 | -3.9434 | 1 |
| 190 | -3.9333 | 1 |
| 191 | -3.9222 | 1 |
| 192 | -3.9104 | 1 |
| 193 | -3.8711 | 1 |
| 194 | -3.8537 | 1 |
| 195 | -3.8482 | 1 |
| 196 | -3.8122 | 1 |
| 197 | -3.8015 | 1 |
| 198 | -3.7983 | 1 |
| 199 | -3.791 | 1 |
| 200 | -3.7659 | 1 |
| 201 | -3.7449 | 1 |
| 202 | -3.7426 | 1 |
| 203 | -3.7094 | 1 |
| 204 | -3.6799 | 1 |
| 205 | -3.6751 | 1 |
| 206 | -3.6557 | 1 |
| 207 | -3.6431 | 1 |
| 208 | -3.6415 | 1 |
| 209 | -3.6342 | 1 |
| 210 | -3.6223 | 1 |
| 211 | -3.5799 | 1 |
| 212 | -3.5719 | 1 |
| 213 | -3.5363 | 1 |
| 214 | -3.4934 | 1 |
| 215 | -3.4918 | 1 |
| 216 | -3.4882 | 1 |
| 217 | -3.4595 | 1 |
| 218 | -3.4478 | 1 |
| 219 | -3.439 | 1 |
| 220 | -3.4382 | 1 |
| 221 | -3.4208 | 1 |
| 222 | -3.4001 | 1 |
| 223 | -3.3923 | 1 |
| 224 | -3.3827 | 1 |
| 225 | -3.3695 | 1 |
| 226 | -3.3344 | 1 |
| 227 | -3.312 | 1 |
| 228 | -3.2904 | 1 |
| 229 | -3.2666 | 1 |
| 230 | -3.2399 | 1 |
| 231 | -3.2085 | 1 |
| 232 | -3.1915 | 1 |
| 233 | -3.1854 | 1 |
| 234 | -3.1802 | 1 |
| 235 | -3.1631 | 1 |
| 236 | -3.1589 | 1 |
| 237 | -3.1434 | 1 |
| 238 | -3.1345 | 1 |
| 239 | -3.1148 | 1 |
| 240 | -3.078 | 1 |
| 241 | -3.0709 | 1 |
| 242 | -3.0315 | 1 |
| 243 | -3.0204 | 1 |
| 244 | -3.0183 | 1 |
| 245 | -2.9834 | 1 |
| 246 | -2.978 | 1 |
| 247 | -2.9693 | 1 |
| 248 | -2.9681 | 1 |
| 249 | -2.954 | 1 |
| 250 | -2.9327 | 1 |
| 251 | -2.9216 | 1 |
| 252 | -2.9197 | 1 |
| 253 | -2.8961 | 1 |
| 254 | -2.8716 | 1 |
| 255 | -2.8394 | 1 |
| 256 | -2.8357 | 1 |
| 257 | -2.8217 | 1 |
| 258 | -2.8149 | 1 |
| 259 | -2.7978 | 1 |
| 260 | -2.7558 | 1 |
| 261 | -2.7514 | 1 |
| 262 | -2.739 | 1 |
| 263 | -2.7188 | 1 |
| 264 | -2.6945 | 1 |
| 265 | -2.6927 | 1 |
| 266 | -2.6361 | 1 |
| 267 | -2.6105 | 1 |
| 268 | -2.5751 | 1 |
| 269 | -2.5054 | 1 |
| 270 | -2.504 | 1 |
| 271 | -2.4904 | 1 |
| 272 | -2.4724 | 1 |
| 273 | -2.447 | 1 |
| 274 | -2.4023 | 1 |
| 275 | -2.361 | 1 |
| 276 | -2.3204 | 1 |
| 277 | -2.3136 | 1 |
| 278 | -2.2925 | 1 |
| 279 | -2.2616 | 1 |
| 280 | -2.242 | 1 |
| 281 | -2.2154 | 1 |
| 282 | -2.2091 | 1 |
| 283 | -2.1865 | 1 |
| 284 | -2.176 | 1 |
| 285 | -2.1461 | 1 |
| 286 | -2.1299 | 1 |
| 287 | -2.0804 | 1 |
| 288 | -2.0586 | 1 |
| 289 | -2.0387 | 1 |
| 290 | -2.0253 | 1 |
| 291 | -2.0019 | 1 |
| 292 | -2.0009 | 1 |
| 293 | -1.973 | 1 |
| 294 | -1.9704 | 1 |
| 295 | -1.9144 | 1 |
| 296 | -1.91 | 1 |
| 297 | -1.9 | 1 |
| 298 | -1.8949 | 1 |
| 299 | -1.8745 | 1 |
| 300 | -1.8212 | 1 |
| 301 | -1.8135 | 1 |
| 302 | -1.7941 | 1 |
| 303 | -1.765 | 1 |
| 304 | -1.728 | 1 |
| 305 | -1.7058 | 1 |
| 306 | -1.7017 | 1 |
| 307 | -1.686 | 1 |
| 308 | -1.6491 | 1 |
| 309 | -1.6319 | 1 |
| 310 | -1.6161 | 1 |
| 311 | -1.6135 | 1 |
| 312 | -1.5834 | 1 |
| 313 | -1.5646 | 1 |
| 314 | -1.5307 | 1 |
| 315 | -1.5221 | 1 |
| 316 | -1.5111 | 1 |
| 317 | -1.4893 | 1 |
| 318 | -1.464 | 1 |
| 319 | -1.4394 | 1 |
| 320 | -1.4346 | 1 |
| 321 | -1.4137 | 1 |
| 322 | -1.3865 | 1 |
| 323 | -1.3496 | 1 |
| 324 | -1.3279 | 1 |
| 325 | -1.3258 | 1 |
| 326 | -1.275 | 1 |
| 327 | -1.2731 | 1 |
| 328 | -1.2568 | 1 |
| 329 | -1.2332 | 1 |
| 330 | -1.229 | 1 |
| 331 | -1.2122 | 1 |
| 332 | -1.1826 | 1 |
| 333 | -1.1771 | 1 |
| 334 | -1.1546 | 1 |
| 335 | -1.1395 | 1 |
| 336 | -1.1319 | 1 |
| 337 | -1.1176 | 1 |
| 338 | -1.0832 | 1 |
| 339 | -1.0452 | 1 |
| 340 | -1.0306 | 1 |
| 341 | -0.999 | 1 |
| 342 | -0.9978 | 1 |
| 343 | -0.9908 | 1 |
| 344 | -0.9668 | 1 |
| 345 | -0.9643 | 1 |
| 346 | -0.9558 | 1 |
| 347 | -0.9436 | 1 |
| 348 | -0.9353 | 1 |
| 349 | -0.9336 | 1 |
| 350 | -0.9122 | 1 |
| 351 | -0.9033 | 1 |
| 352 | -0.8767 | 1 |
| 353 | -0.8675 | 1 |
| 354 | -0.8667 | 1 |
| 355 | -0.8619 | 1 |
| 356 | -0.8532 | 1 |
| 357 | -0.7945 | 1 |
| 358 | -0.7935 | 1 |
| 359 | -0.7757 | 1 |
| 360 | -0.7511 | 1 |
| 361 | -0.7454 | 1 |
| 362 | -0.7359 | 1 |
| 363 | -0.6941 | 1 |
| 364 | -0.6902 | 1 |
| 365 | -0.6833 | 1 |
| 366 | -0.6559 | 1 |
| 367 | -0.6482 | 1 |
| 368 | -0.5812 | 1 |
| 369 | -0.0846 | 1 |
| 370 | -0.0194 | 1 |
| 371 | -0.0135 | 1 |
| 372 | 0.0097 | 1 |
| 373 | 0.0335 | 1 |
| **374** | **0.1306** | **1** |
| **375** | **2.5638** | **1** |
| **376** | **2.5936** | **1** |
| **377** | **4.7779** | **0** |
| 378 | 4.8935 | 0 |
| 379 | 4.9183 | 0 |
| 380 | 5.0158 | 0 |
| 381 | 5.0584 | 0 |
| 382 | 5.0706 | 0 |
| 383 | 5.0909 | 0 |
| 384 | 5.1059 | 0 |
| 385 | 5.3475 | 0 |
| 386 | 5.3726 | 0 |
| 387 | 5.3814 | 0 |
| 388 | 5.3919 | 0 |
| 389 | 5.4049 | 0 |
| 390 | 5.4301 | 0 |
| 391 | 5.4463 | 0 |
| 392 | 5.4477 | 0 |
| 393 | 5.4606 | 0 |
| 394 | 5.479 | 0 |
| 395 | 5.492 | 0 |
| 396 | 5.4938 | 0 |
| 397 | 5.4968 | 0 |
| 398 | 5.5016 | 0 |
| 399 | 5.525 | 0 |
| 400 | 5.5324 | 0 |
| 401 | 5.5655 | 0 |
| 402 | 5.5741 | 0 |
| 403 | 5.5757 | 0 |
| 404 | 5.6001 | 0 |
| 405 | 5.6098 | 0 |
| 406 | 5.635 | 0 |
| 407 | 5.6361 | 0 |
| 408 | 5.7182 | 0 |
| 409 | 5.7888 | 0 |
| 410 | 5.8068 | 0 |
| 411 | 5.8146 | 0 |
| 412 | 5.8767 | 0 |
| 413 | 5.9651 | 0 |
| 414 | 5.9688 | 0 |
| 415 | 6.0412 | 0 |
| 416 | 6.0621 | 0 |
| 417 | 6.0944 | 0 |
| 418 | 6.1033 | 0 |
| 419 | 6.1111 | 0 |
| 420 | 6.1394 | 0 |
| 421 | 6.142 | 0 |
| 422 | 6.1972 | 0 |
| 423 | 6.209 | 0 |
| 424 | 6.2173 | 0 |
| 425 | 6.2374 | 0 |
| 426 | 6.2652 | 0 |
| 427 | 6.2813 | 0 |
| 428 | 6.2894 | 0 |
| 429 | 6.3161 | 0 |
| 430 | 6.334 | 0 |
| 431 | 6.3558 | 0 |
| 432 | 6.365 | 0 |
| 433 | 6.3847 | 0 |
| 434 | 6.3931 | 0 |
| 435 | 6.4133 | 0 |
| 436 | 6.4217 | 0 |
| 437 | 6.4288 | 0 |
| 438 | 6.4406 | 0 |
| 439 | 6.4746 | 0 |
| 440 | 6.4949 | 0 |
| 441 | 6.5273 | 0 |
| 442 | 6.631 | 0 |
| 443 | 6.6799 | 0 |
| 444 | 6.7367 | 0 |
| 445 | 6.7504 | 0 |
| 446 | 6.8134 | 0 |
| 447 | 6.8611 | 0 |
| 448 | 6.904 | 0 |
| 449 | 7.1295 | 0 |
| 450 | 7.3043 | 0 |
| 451 | 7.3802 | 0 |
| 452 | 7.6331 | 0 |
| 453 | 7.7162 | 0 |
| 454 | 8.1065 | 0 |
| 455 | 8.1387 | 0 |
| 456 | 8.5134 | 0 |
| 457 | 8.6285 | 0 |
| 458 | 8.6366 | 0 |
| 459 | 9.204 | 0 |
| 460 | 9.3358 | 0 |
| 461 | 9.3596 | 0 |
| 462 | 9.4191 | 0 |
| 463 | 9.5451 | 0 |
| 464 | 9.6056 | 0 |
| 465 | 9.6853 | 0 |
| 466 | 9.7736 | 0 |
| 467 | 9.7882 | 0 |
| 468 | 9.8371 | 0 |
| 469 | 9.9413 | 0 |
| 470 | 10.0657 | 0 |
| 471 | 10.1256 | 0 |
| 472 | 10.2361 | 0 |
| 473 | 10.4619 | 0 |
| 474 | 10.5232 | 0 |
| 475 | 10.5715 | 0 |
| 476 | 10.7097 | 0 |
| 477 | 10.8412 | 0 |
| 478 | 11.0023 | 0 |
| 479 | 11.0166 | 0 |
| 480 | 11.2202 | 0 |

**Supplementary Table 11.** DFT calculation result on electron occupation of Ca-doped Li_3_VO_4_ (spin component 2; occupation: 1 = filled, 0 = empty).

| band number | band energies (eV) | occupation |
| --- | --- | --- |
| 1 | -69.5173 | 1 |
| 2 | -69.4047 | 1 |
| 3 | -69.3298 | 1 |
| 4 | -69.3164 | 1 |
| 5 | -69.0401 | 1 |
| 6 | -69.04 | 1 |
| 7 | -69.007 | 1 |
| 8 | -69.0052 | 1 |
| 9 | -68.9259 | 1 |
| 10 | -68.9249 | 1 |
| 11 | -68.9088 | 1 |
| 12 | -68.8945 | 1 |
| 13 | -68.8939 | 1 |
| 14 | -68.8798 | 1 |
| 15 | -65.7272 | 1 |
| 16 | -65.7111 | 1 |
| 17 | -45.7279 | 1 |
| 18 | -45.7148 | 1 |
| 19 | -45.345 | 1 |
| 20 | -45.3335 | 1 |
| 21 | -45.2876 | 1 |
| 22 | -45.2871 | 1 |
| 23 | -45.2851 | 1 |
| 24 | -45.2819 | 1 |
| 25 | -45.2671 | 1 |
| 26 | -45.263 | 1 |
| 27 | -45.2628 | 1 |
| 28 | -45.2609 | 1 |
| 29 | -45.2459 | 1 |
| 30 | -45.2431 | 1 |
| 31 | -45.2336 | 1 |
| 32 | -45.2303 | 1 |
| 33 | -45.2224 | 1 |
| 34 | -45.2196 | 1 |
| 35 | -45.2077 | 1 |
| 36 | -45.2065 | 1 |
| 37 | -45.2043 | 1 |
| 38 | -45.2024 | 1 |
| 39 | -45.1601 | 1 |
| 40 | -45.1542 | 1 |
| 41 | -45.1159 | 1 |
| 42 | -45.1119 | 1 |
| 43 | -45.0914 | 1 |
| 44 | -45.0906 | 1 |
| 45 | -45.0877 | 1 |
| 46 | -45.0873 | 1 |
| 47 | -45.0845 | 1 |
| 48 | -45.081 | 1 |
| 49 | -45.0594 | 1 |
| 50 | -45.043 | 1 |
| 51 | -44.9451 | 1 |
| 52 | -44.9432 | 1 |
| 53 | -44.9387 | 1 |
| 54 | -44.9359 | 1 |
| 55 | -44.9234 | 1 |
| 56 | -44.9159 | 1 |
| 57 | -44.9097 | 1 |
| 58 | -44.9065 | 1 |
| 59 | -44.6439 | 1 |
| 60 | -44.6356 | 1 |
| 61 | -44.6233 | 1 |
| 62 | -44.6178 | 1 |
| 63 | -41.759 | 1 |
| 64 | -41.7238 | 1 |
| 65 | -41.711 | 1 |
| 66 | -41.6508 | 1 |
| 67 | -41.6196 | 1 |
| 68 | -41.6062 | 1 |
| 69 | -41.5839 | 1 |
| 70 | -41.5706 | 1 |
| 71 | -41.5445 | 1 |
| 72 | -41.5405 | 1 |
| 73 | -41.5306 | 1 |
| 74 | -41.5302 | 1 |
| 75 | -41.292 | 1 |
| 76 | -41.2919 | 1 |
| 77 | -41.2776 | 1 |
| 78 | -41.2774 | 1 |
| 79 | -41.2767 | 1 |
| 80 | -41.2765 | 1 |
| 81 | -41.2643 | 1 |
| 82 | -41.2623 | 1 |
| 83 | -41.2478 | 1 |
| 84 | -41.2462 | 1 |
| 85 | -41.242 | 1 |
| 86 | -41.2402 | 1 |
| 87 | -41.1826 | 1 |
| 88 | -41.1818 | 1 |
| 89 | -41.1754 | 1 |
| 90 | -41.1744 | 1 |
| 91 | -41.1724 | 1 |
| 92 | -41.1715 | 1 |
| 93 | -41.1703 | 1 |
| 94 | -41.1487 | 1 |
| 95 | -41.1433 | 1 |
| 96 | -41.1417 | 1 |
| 97 | -41.1397 | 1 |
| 98 | -41.1335 | 1 |
| 99 | -41.1333 | 1 |
| 100 | -41.132 | 1 |
| 101 | -41.1265 | 1 |
| 102 | -41.1254 | 1 |
| 103 | -41.1212 | 1 |
| 104 | -41.104 | 1 |
| 105 | -39.6956 | 1 |
| 106 | -39.6868 | 1 |
| 107 | -38.8852 | 1 |
| 108 | -38.7951 | 1 |
| 109 | -38.0191 | 1 |
| 110 | -37.703 | 1 |
| 111 | -37.3823 | 1 |
| 112 | -37.2033 | 1 |
| 113 | -19.9326 | 1 |
| 114 | -19.9124 | 1 |
| 115 | -19.7758 | 1 |
| 116 | -19.7694 | 1 |
| 117 | -19.7661 | 1 |
| 118 | -19.7229 | 1 |
| 119 | -18.4313 | 1 |
| 120 | -18.3729 | 1 |
| 121 | -18.2292 | 1 |
| 122 | -18.215 | 1 |
| 123 | -18.1871 | 1 |
| 124 | -18.1822 | 1 |
| 125 | -18.1181 | 1 |
| 126 | -18.099 | 1 |
| 127 | -18.0681 | 1 |
| 128 | -18.0491 | 1 |
| 129 | -18.0481 | 1 |
| 130 | -17.971 | 1 |
| 131 | -17.9477 | 1 |
| 132 | -17.9308 | 1 |
| 133 | -17.9271 | 1 |
| 134 | -17.905 | 1 |
| 135 | -17.8962 | 1 |
| 136 | -17.884 | 1 |
| 137 | -17.8396 | 1 |
| 138 | -17.8244 | 1 |
| 139 | -17.7873 | 1 |
| 140 | -17.7844 | 1 |
| 141 | -17.7647 | 1 |
| 142 | -17.7478 | 1 |
| 143 | -17.6925 | 1 |
| 144 | -17.677 | 1 |
| 145 | -17.661 | 1 |
| 146 | -17.6573 | 1 |
| 147 | -17.6374 | 1 |
| 148 | -17.6281 | 1 |
| 149 | -17.6245 | 1 |
| 150 | -17.6193 | 1 |
| 151 | -17.6169 | 1 |
| 152 | -17.6075 | 1 |
| 153 | -17.5963 | 1 |
| 154 | -17.595 | 1 |
| 155 | -17.5922 | 1 |
| 156 | -17.5809 | 1 |
| 157 | -17.5787 | 1 |
| 158 | -17.5767 | 1 |
| 159 | -17.563 | 1 |
| 160 | -17.5604 | 1 |
| 161 | -17.5562 | 1 |
| 162 | -17.5491 | 1 |
| 163 | -17.5398 | 1 |
| 164 | -17.5308 | 1 |
| 165 | -17.4953 | 1 |
| 166 | -17.4945 | 1 |
| 167 | -17.4709 | 1 |
| 168 | -17.4558 | 1 |
| 169 | -17.4431 | 1 |
| 170 | -17.4369 | 1 |
| 171 | -17.4357 | 1 |
| 172 | -17.4294 | 1 |
| 173 | -17.4214 | 1 |
| 174 | -17.404 | 1 |
| 175 | -16.8023 | 1 |
| 176 | -16.7067 | 1 |
| 177 | -16.4118 | 1 |
| 178 | -16.3765 | 1 |
| 179 | -16.2751 | 1 |
| 180 | -16.2445 | 1 |
| 181 | -16.0806 | 1 |
| 182 | -15.9833 | 1 |
| 183 | -4.1906 | 1 |
| 184 | -4.1581 | 1 |
| 185 | -4.0468 | 1 |
| 186 | -4.0293 | 1 |
| 187 | -4.023 | 1 |
| 188 | -3.9896 | 1 |
| 189 | -3.9431 | 1 |
| 190 | -3.9328 | 1 |
| 191 | -3.9195 | 1 |
| 192 | -3.9094 | 1 |
| 193 | -3.8698 | 1 |
| 194 | -3.8475 | 1 |
| 195 | -3.8473 | 1 |
| 196 | -3.8115 | 1 |
| 197 | -3.7983 | 1 |
| 198 | -3.796 | 1 |
| 199 | -3.7899 | 1 |
| 200 | -3.7652 | 1 |
| 201 | -3.7447 | 1 |
| 202 | -3.742 | 1 |
| 203 | -3.7017 | 1 |
| 204 | -3.6789 | 1 |
| 205 | -3.6733 | 1 |
| 206 | -3.6555 | 1 |
| 207 | -3.6425 | 1 |
| 208 | -3.6328 | 1 |
| 209 | -3.6263 | 1 |
| 210 | -3.6173 | 1 |
| 211 | -3.5797 | 1 |
| 212 | -3.5342 | 1 |
| 213 | -3.5311 | 1 |
| 214 | -3.4928 | 1 |
| 215 | -3.4885 | 1 |
| 216 | -3.4875 | 1 |
| 217 | -3.4466 | 1 |
| 218 | -3.4385 | 1 |
| 219 | -3.4358 | 1 |
| 220 | -3.4308 | 1 |
| 221 | -3.4138 | 1 |
| 222 | -3.3855 | 1 |
| 223 | -3.3811 | 1 |
| 224 | -3.3695 | 1 |
| 225 | -3.3291 | 1 |
| 226 | -3.3043 | 1 |
| 227 | -3.2907 | 1 |
| 228 | -3.273 | 1 |
| 229 | -3.2625 | 1 |
| 230 | -3.228 | 1 |
| 231 | -3.1915 | 1 |
| 232 | -3.1891 | 1 |
| 233 | -3.1814 | 1 |
| 234 | -3.1699 | 1 |
| 235 | -3.1612 | 1 |
| 236 | -3.1495 | 1 |
| 237 | -3.1419 | 1 |
| 238 | -3.1323 | 1 |
| 239 | -3.0996 | 1 |
| 240 | -3.054 | 1 |
| 241 | -3.0493 | 1 |
| 242 | -3.0182 | 1 |
| 243 | -3.015 | 1 |
| 244 | -2.9832 | 1 |
| 245 | -2.9744 | 1 |
| 246 | -2.9691 | 1 |
| 247 | -2.9665 | 1 |
| 248 | -2.9595 | 1 |
| 249 | -2.9498 | 1 |
| 250 | -2.9297 | 1 |
| 251 | -2.9066 | 1 |
| 252 | -2.8765 | 1 |
| 253 | -2.8608 | 1 |
| 254 | -2.8351 | 1 |
| 255 | -2.8324 | 1 |
| 256 | -2.821 | 1 |
| 257 | -2.7986 | 1 |
| 258 | -2.791 | 1 |
| 259 | -2.7842 | 1 |
| 260 | -2.7463 | 1 |
| 261 | -2.7204 | 1 |
| 262 | -2.6939 | 1 |
| 263 | -2.6892 | 1 |
| 264 | -2.6309 | 1 |
| 265 | -2.6117 | 1 |
| 266 | -2.571 | 1 |
| 267 | -2.5088 | 1 |
| 268 | -2.5032 | 1 |
| 269 | -2.4969 | 1 |
| 270 | -2.4749 | 1 |
| 271 | -2.4506 | 1 |
| 272 | -2.3987 | 1 |
| 273 | -2.3583 | 1 |
| 274 | -2.3458 | 1 |
| 275 | -2.3161 | 1 |
| 276 | -2.287 | 1 |
| 277 | -2.2667 | 1 |
| 278 | -2.2392 | 1 |
| 279 | -2.2383 | 1 |
| 280 | -2.2037 | 1 |
| 281 | -2.1844 | 1 |
| 282 | -2.1745 | 1 |
| 283 | -2.1396 | 1 |
| 284 | -2.1227 | 1 |
| 285 | -2.1096 | 1 |
| 286 | -2.0718 | 1 |
| 287 | -2.036 | 1 |
| 288 | -2.0301 | 1 |
| 289 | -2.005 | 1 |
| 290 | -1.9922 | 1 |
| 291 | -1.9779 | 1 |
| 292 | -1.9687 | 1 |
| 293 | -1.909 | 1 |
| 294 | -1.9004 | 1 |
| 295 | -1.8854 | 1 |
| 296 | -1.8789 | 1 |
| 297 | -1.8721 | 1 |
| 298 | -1.815 | 1 |
| 299 | -1.804 | 1 |
| 300 | -1.7831 | 1 |
| 301 | -1.7385 | 1 |
| 302 | -1.7309 | 1 |
| 303 | -1.7053 | 1 |
| 304 | -1.6903 | 1 |
| 305 | -1.67 | 1 |
| 306 | -1.6256 | 1 |
| 307 | -1.6097 | 1 |
| 308 | -1.6041 | 1 |
| 309 | -1.6001 | 1 |
| 310 | -1.566 | 1 |
| 311 | -1.5608 | 1 |
| 312 | -1.5212 | 1 |
| 313 | -1.5164 | 1 |
| 314 | -1.5025 | 1 |
| 315 | -1.473 | 1 |
| 316 | -1.4677 | 1 |
| 317 | -1.4454 | 1 |
| 318 | -1.4379 | 1 |
| 319 | -1.4111 | 1 |
| 320 | -1.4061 | 1 |
| 321 | -1.394 | 1 |
| 322 | -1.3685 | 1 |
| 323 | -1.3383 | 1 |
| 324 | -1.3243 | 1 |
| 325 | -1.3113 | 1 |
| 326 | -1.2685 | 1 |
| 327 | -1.2486 | 1 |
| 328 | -1.2363 | 1 |
| 329 | -1.2173 | 1 |
| 330 | -1.2037 | 1 |
| 331 | -1.1825 | 1 |
| 332 | -1.18 | 1 |
| 333 | -1.16 | 1 |
| 334 | -1.1477 | 1 |
| 335 | -1.1283 | 1 |
| 336 | -1.1249 | 1 |
| 337 | -1.098 | 1 |
| 338 | -1.0773 | 1 |
| 339 | -1.0428 | 1 |
| 340 | -1.0175 | 1 |
| 341 | -0.9991 | 1 |
| 342 | -0.9919 | 1 |
| 343 | -0.9739 | 1 |
| 344 | -0.9637 | 1 |
| 345 | -0.959 | 1 |
| 346 | -0.9532 | 1 |
| 347 | -0.9342 | 1 |
| 348 | -0.9319 | 1 |
| 349 | -0.9294 | 1 |
| 350 | -0.8931 | 1 |
| 351 | -0.8862 | 1 |
| 352 | -0.8742 | 1 |
| 353 | -0.8666 | 1 |
| 354 | -0.8556 | 1 |
| 355 | -0.8531 | 1 |
| 356 | -0.8166 | 1 |
| 357 | -0.7963 | 1 |
| 358 | -0.7928 | 1 |
| 359 | -0.7484 | 1 |
| 360 | -0.7429 | 1 |
| 361 | -0.7325 | 1 |
| 362 | -0.7101 | 1 |
| 363 | -0.6887 | 1 |
| 364 | -0.6832 | 1 |
| 365 | -0.6752 | 1 |
| 366 | -0.6574 | 1 |
| 367 | -0.6476 | 1 |
| 368 | -0.5752 | 1 |
| 369 | -0.0814 | 1 |
| 370 | -0.0479 | 1 |
| 371 | 0.0321 | 1 |
| 372 | 0.0524 | 1 |
| 373 | 0.0757 | 1 |
| **374** | **0.2718** | **1** |
| **375** | **4.7801** | **0** |
| 376 | 4.8944 | 0 |
| 377 | 4.9192 | 0 |
| 378 | 5.0176 | 0 |
| 379 | 5.0645 | 0 |
| 380 | 5.0739 | 0 |
| 381 | 5.0933 | 0 |
| 382 | 5.1157 | 0 |
| 383 | 5.3492 | 0 |
| 384 | 5.3726 | 0 |
| 385 | 5.3822 | 0 |
| 386 | 5.3918 | 0 |
| 387 | 5.406 | 0 |
| 388 | 5.4323 | 0 |
| 389 | 5.4479 | 0 |
| 390 | 5.448 | 0 |
| 391 | 5.4609 | 0 |
| 392 | 5.4804 | 0 |
| 393 | 5.4935 | 0 |
| 394 | 5.495 | 0 |
| 395 | 5.498 | 0 |
| 396 | 5.5031 | 0 |
| 397 | 5.5269 | 0 |
| 398 | 5.5336 | 0 |
| 399 | 5.5671 | 0 |
| 400 | 5.5765 | 0 |
| 401 | 5.577 | 0 |
| 402 | 5.6014 | 0 |
| 403 | 5.6118 | 0 |
| 404 | 5.6369 | 0 |
| 405 | 5.6374 | 0 |
| 406 | 5.721 | 0 |
| 407 | 5.7894 | 0 |
| 408 | 5.8063 | 0 |
| 409 | 5.817 | 0 |
| 410 | 5.8784 | 0 |
| 411 | 5.9659 | 0 |
| 412 | 5.9681 | 0 |
| 413 | 6.0415 | 0 |
| 414 | 6.0626 | 0 |
| 415 | 6.094 | 0 |
| 416 | 6.1082 | 0 |
| 417 | 6.1106 | 0 |
| 418 | 6.1401 | 0 |
| 419 | 6.1418 | 0 |
| 420 | 6.1999 | 0 |
| 421 | 6.2136 | 0 |
| 422 | 6.2202 | 0 |
| 423 | 6.2389 | 0 |
| 424 | 6.2685 | 0 |
| 425 | 6.2814 | 0 |
| 426 | 6.2902 | 0 |
| 427 | 6.3169 | 0 |
| 428 | 6.3474 | 0 |
| 429 | 6.3563 | 0 |
| 430 | 6.3663 | 0 |
| 431 | 6.3845 | 0 |
| 432 | 6.4008 | 0 |
| 433 | 6.415 | 0 |
| 434 | 6.4205 | 0 |
| 435 | 6.4352 | 0 |
| 436 | 6.4424 | 0 |
| 437 | 6.474 | 0 |
| 438 | 6.4932 | 0 |
| 439 | 6.5271 | 0 |
| 440 | 6.6323 | 0 |
| 441 | 6.6746 | 0 |
| 442 | 6.7422 | 0 |
| 443 | 6.753 | 0 |
| 444 | 6.8211 | 0 |
| 445 | 7.1307 | 0 |
| 446 | 7.529 | 0 |
| 447 | 7.5395 | 0 |
| 448 | 7.73 | 0 |
| 449 | 7.857 | 0 |
| 450 | 7.9924 | 0 |
| 451 | 8.0536 | 0 |
| 452 | 8.4226 | 0 |
| 453 | 8.4432 | 0 |
| 454 | 8.5204 | 0 |
| 455 | 8.5394 | 0 |
| 456 | 8.735 | 0 |
| 457 | 8.9133 | 0 |
| 458 | 8.988 | 0 |
| 459 | 9.2418 | 0 |
| 460 | 9.3198 | 0 |
| 461 | 9.3902 | 0 |
| 462 | 9.5125 | 0 |
| 463 | 9.6982 | 0 |
| 464 | 9.7475 | 0 |
| 465 | 9.8276 | 0 |
| 466 | 9.8566 | 0 |
| 467 | 9.9044 | 0 |
| 468 | 9.9163 | 0 |
| 469 | 10.0508 | 0 |
| 470 | 10.064 | 0 |
| 471 | 10.2606 | 0 |
| 472 | 10.3239 | 0 |
| 473 | 10.4577 | 0 |
| 474 | 10.5238 | 0 |
| 475 | 10.6333 | 0 |
| 476 | 10.7158 | 0 |
| 477 | 10.8252 | 0 |
| 478 | 10.8499 | 0 |
| 479 | 11.0325 | 0 |
| 480 | 11.1533 | 0 |

**Supplementary Table 12.** DFT calculation result on electron occupation of Ca-doped Li_3_VO_4_ with formation of oxygen vacancy (spin component 1; occupation: 1 = filled, 0 = empty).

| band number | band energies (eV) | occupation |
| --- | --- | --- |
| 1 | -69.3154 | 1 |
| 2 | -69.2512 | 1 |
| 3 | -69.2252 | 1 |
| 4 | -69.0917 | 1 |
| 5 | -69.091 | 1 |
| 6 | -69.0847 | 1 |
| 7 | -69.0806 | 1 |
| 8 | -69.0785 | 1 |
| 9 | -69.0528 | 1 |
| 10 | -69.052 | 1 |
| 11 | -69.0368 | 1 |
| 12 | -69.0318 | 1 |
| 13 | -68.9857 | 1 |
| 14 | -67.9955 | 1 |
| 15 | -67.9935 | 1 |
| 16 | -67.1082 | 1 |
| 17 | -45.5982 | 1 |
| 18 | -45.5461 | 1 |
| 19 | -45.5453 | 1 |
| 20 | -45.535 | 1 |
| 21 | -45.261 | 1 |
| 22 | -45.2567 | 1 |
| 23 | -45.2539 | 1 |
| 24 | -45.242 | 1 |
| 25 | -45.2239 | 1 |
| 26 | -45.2001 | 1 |
| 27 | -45.196 | 1 |
| 28 | -45.1911 | 1 |
| 29 | -45.1849 | 1 |
| 30 | -45.1825 | 1 |
| 31 | -45.1579 | 1 |
| 32 | -45.1544 | 1 |
| 33 | -45.1314 | 1 |
| 34 | -45.1303 | 1 |
| 35 | -45.0853 | 1 |
| 36 | -45.0831 | 1 |
| 37 | -45.0802 | 1 |
| 38 | -45.0782 | 1 |
| 39 | -45.0695 | 1 |
| 40 | -45.0668 | 1 |
| 41 | -45.0498 | 1 |
| 42 | -45.0446 | 1 |
| 43 | -45.0413 | 1 |
| 44 | -45.0383 | 1 |
| 45 | -45.0058 | 1 |
| 46 | -45.0021 | 1 |
| 47 | -44.9816 | 1 |
| 48 | -44.9769 | 1 |
| 49 | -44.9618 | 1 |
| 50 | -44.9596 | 1 |
| 51 | -44.9575 | 1 |
| 52 | -44.955 | 1 |
| 53 | -44.9231 | 1 |
| 54 | -44.9207 | 1 |
| 55 | -44.8838 | 1 |
| 56 | -44.8812 | 1 |
| 57 | -44.8534 | 1 |
| 58 | -44.8477 | 1 |
| 59 | -44.8401 | 1 |
| 60 | -44.8375 | 1 |
| 61 | -44.7307 | 1 |
| 62 | -44.7273 | 1 |
| 63 | -41.5564 | 1 |
| 64 | -41.5399 | 1 |
| 65 | -41.5368 | 1 |
| 66 | -41.4984 | 1 |
| 67 | -41.4816 | 1 |
| 68 | -41.4754 | 1 |
| 69 | -41.4725 | 1 |
| 70 | -41.4428 | 1 |
| 71 | -41.442 | 1 |
| 72 | -41.3389 | 1 |
| 73 | -41.3381 | 1 |
| 74 | -41.3274 | 1 |
| 75 | -41.3268 | 1 |
| 76 | -41.325 | 1 |
| 77 | -41.3241 | 1 |
| 78 | -41.3218 | 1 |
| 79 | -41.316 | 1 |
| 80 | -41.3153 | 1 |
| 81 | -41.3045 | 1 |
| 82 | -41.3008 | 1 |
| 83 | -41.2967 | 1 |
| 84 | -41.2948 | 1 |
| 85 | -41.2947 | 1 |
| 86 | -41.2926 | 1 |
| 87 | -41.2874 | 1 |
| 88 | -41.2867 | 1 |
| 89 | -41.2781 | 1 |
| 90 | -41.2765 | 1 |
| 91 | -41.2759 | 1 |
| 92 | -41.27 | 1 |
| 93 | -41.2693 | 1 |
| 94 | -41.2631 | 1 |
| 95 | -41.2605 | 1 |
| 96 | -41.2573 | 1 |
| 97 | -41.256 | 1 |
| 98 | -41.2518 | 1 |
| 99 | -41.2185 | 1 |
| 100 | -41.2103 | 1 |
| 101 | -41.2009 | 1 |
| 102 | -40.3551 | 1 |
| 103 | -40.3197 | 1 |
| 104 | -40.298 | 1 |
| 105 | -40.282 | 1 |
| 106 | -40.2784 | 1 |
| 107 | -40.2332 | 1 |
| 108 | -40.2308 | 1 |
| 109 | -40.0011 | 1 |
| 110 | -39.6308 | 1 |
| 111 | -39.626 | 1 |
| 112 | -38.8418 | 1 |
| 113 | -20.414 | 1 |
| 114 | -20.2817 | 1 |
| 115 | -20.1514 | 1 |
| 116 | -20.1425 | 1 |
| 117 | -20.1058 | 1 |
| 118 | -20.0254 | 1 |
| 119 | -18.3442 | 1 |
| 120 | -18.2817 | 1 |
| 121 | -18.2299 | 1 |
| 122 | -18.1834 | 1 |
| 123 | -18.1007 | 1 |
| 124 | -18.0893 | 1 |
| 125 | -18.0801 | 1 |
| 126 | -18.073 | 1 |
| 127 | -18.0318 | 1 |
| 128 | -18.0157 | 1 |
| 129 | -18.0031 | 1 |
| 130 | -17.9761 | 1 |
| 131 | -17.9432 | 1 |
| 132 | -17.8322 | 1 |
| 133 | -17.8317 | 1 |
| 134 | -17.8245 | 1 |
| 135 | -17.8078 | 1 |
| 136 | -17.7648 | 1 |
| 137 | -17.7318 | 1 |
| 138 | -17.7298 | 1 |
| 139 | -17.7053 | 1 |
| 140 | -17.7013 | 1 |
| 141 | -17.6755 | 1 |
| 142 | -17.6744 | 1 |
| 143 | -17.6658 | 1 |
| 144 | -17.6582 | 1 |
| 145 | -17.648 | 1 |
| 146 | -17.6299 | 1 |
| 147 | -17.6278 | 1 |
| 148 | -17.6142 | 1 |
| 149 | -17.6122 | 1 |
| 150 | -17.5917 | 1 |
| 151 | -17.5874 | 1 |
| 152 | -17.586 | 1 |
| 153 | -17.5799 | 1 |
| 154 | -17.579 | 1 |
| 155 | -17.5703 | 1 |
| 156 | -17.5691 | 1 |
| 157 | -17.5572 | 1 |
| 158 | -17.5468 | 1 |
| 159 | -17.5442 | 1 |
| 160 | -17.5359 | 1 |
| 161 | -17.5339 | 1 |
| 162 | -17.5237 | 1 |
| 163 | -17.517 | 1 |
| 164 | -17.5162 | 1 |
| 165 | -17.5089 | 1 |
| 166 | -17.4935 | 1 |
| 167 | -17.4871 | 1 |
| 168 | -17.4847 | 1 |
| 169 | -17.4794 | 1 |
| 170 | -17.4521 | 1 |
| 171 | -17.4413 | 1 |
| 172 | -17.1951 | 1 |
| 173 | -17.0872 | 1 |
| 174 | -17.0729 | 1 |
| 175 | -16.9755 | 1 |
| 176 | -16.8343 | 1 |
| 177 | -16.8069 | 1 |
| 178 | -16.7967 | 1 |
| 179 | -16.7786 | 1 |
| 180 | -16.6436 | 1 |
| 181 | -16.5556 | 1 |
| 182 | -4.0752 | 1 |
| 183 | -3.9859 | 1 |
| 184 | -3.967 | 1 |
| 185 | -3.9373 | 1 |
| 186 | -3.9278 | 1 |
| 187 | -3.9253 | 1 |
| 188 | -3.8972 | 1 |
| 189 | -3.8693 | 1 |
| 190 | -3.8494 | 1 |
| 191 | -3.8444 | 1 |
| 192 | -3.8253 | 1 |
| 193 | -3.798 | 1 |
| 194 | -3.7945 | 1 |
| 195 | -3.7652 | 1 |
| 196 | -3.7611 | 1 |
| 197 | -3.741 | 1 |
| 198 | -3.7129 | 1 |
| 199 | -3.7115 | 1 |
| 200 | -3.7014 | 1 |
| 201 | -3.6791 | 1 |
| 202 | -3.649 | 1 |
| 203 | -3.6421 | 1 |
| 204 | -3.6171 | 1 |
| 205 | -3.6127 | 1 |
| 206 | -3.5886 | 1 |
| 207 | -3.5695 | 1 |
| 208 | -3.559 | 1 |
| 209 | -3.555 | 1 |
| 210 | -3.537 | 1 |
| 211 | -3.5315 | 1 |
| 212 | -3.5162 | 1 |
| 213 | -3.4961 | 1 |
| 214 | -3.4678 | 1 |
| 215 | -3.4521 | 1 |
| 216 | -3.425 | 1 |
| 217 | -3.4212 | 1 |
| 218 | -3.3929 | 1 |
| 219 | -3.3826 | 1 |
| 220 | -3.379 | 1 |
| 221 | -3.3614 | 1 |
| 222 | -3.3542 | 1 |
| 223 | -3.3308 | 1 |
| 224 | -3.3299 | 1 |
| 225 | -3.2756 | 1 |
| 226 | -3.2533 | 1 |
| 227 | -3.2164 | 1 |
| 228 | -3.204 | 1 |
| 229 | -3.1606 | 1 |
| 230 | -3.141 | 1 |
| 231 | -3.1361 | 1 |
| 232 | -3.1249 | 1 |
| 233 | -3.1227 | 1 |
| 234 | -3.1186 | 1 |
| 235 | -3.1084 | 1 |
| 236 | -3.0752 | 1 |
| 237 | -3.0746 | 1 |
| 238 | -3.035 | 1 |
| 239 | -3.0275 | 1 |
| 240 | -3.0179 | 1 |
| 241 | -2.9908 | 1 |
| 242 | -2.9634 | 1 |
| 243 | -2.9609 | 1 |
| 244 | -2.9295 | 1 |
| 245 | -2.9262 | 1 |
| 246 | -2.9113 | 1 |
| 247 | -2.8972 | 1 |
| 248 | -2.868 | 1 |
| 249 | -2.866 | 1 |
| 250 | -2.8554 | 1 |
| 251 | -2.8256 | 1 |
| 252 | -2.8023 | 1 |
| 253 | -2.796 | 1 |
| 254 | -2.7858 | 1 |
| 255 | -2.7689 | 1 |
| 256 | -2.7598 | 1 |
| 257 | -2.738 | 1 |
| 258 | -2.7112 | 1 |
| 259 | -2.7021 | 1 |
| 260 | -2.6842 | 1 |
| 261 | -2.6505 | 1 |
| 262 | -2.6386 | 1 |
| 263 | -2.6049 | 1 |
| 264 | -2.5468 | 1 |
| 265 | -2.5351 | 1 |
| 266 | -2.5243 | 1 |
| 267 | -2.4957 | 1 |
| 268 | -2.4826 | 1 |
| 269 | -2.4468 | 1 |
| 270 | -2.4446 | 1 |
| 271 | -2.3888 | 1 |
| 272 | -2.3789 | 1 |
| 273 | -2.3742 | 1 |
| 274 | -2.3316 | 1 |
| 275 | -2.3304 | 1 |
| 276 | -2.2712 | 1 |
| 277 | -2.2548 | 1 |
| 278 | -2.2258 | 1 |
| 279 | -2.2125 | 1 |
| 280 | -2.2106 | 1 |
| 281 | -2.2013 | 1 |
| 282 | -2.1578 | 1 |
| 283 | -2.1357 | 1 |
| 284 | -2.1161 | 1 |
| 285 | -2.1157 | 1 |
| 286 | -2.1081 | 1 |
| 287 | -2.0998 | 1 |
| 288 | -2.0768 | 1 |
| 289 | -2.0614 | 1 |
| 290 | -2.019 | 1 |
| 291 | -1.9754 | 1 |
| 292 | -1.966 | 1 |
| 293 | -1.9656 | 1 |
| 294 | -1.9145 | 1 |
| 295 | -1.9021 | 1 |
| 296 | -1.8859 | 1 |
| 297 | -1.8725 | 1 |
| 298 | -1.8609 | 1 |
| 299 | -1.8544 | 1 |
| 300 | -1.8089 | 1 |
| 301 | -1.793 | 1 |
| 302 | -1.7881 | 1 |
| 303 | -1.7265 | 1 |
| 304 | -1.6992 | 1 |
| 305 | -1.6834 | 1 |
| 306 | -1.664 | 1 |
| 307 | -1.6525 | 1 |
| 308 | -1.6479 | 1 |
| 309 | -1.5821 | 1 |
| 310 | -1.5814 | 1 |
| 311 | -1.5545 | 1 |
| 312 | -1.5351 | 1 |
| 313 | -1.4999 | 1 |
| 314 | -1.4896 | 1 |
| 315 | -1.4407 | 1 |
| 316 | -1.4303 | 1 |
| 317 | -1.4099 | 1 |
| 318 | -1.3871 | 1 |
| 319 | -1.3767 | 1 |
| 320 | -1.3616 | 1 |
| 321 | -1.347 | 1 |
| 322 | -1.3115 | 1 |
| 323 | -1.2978 | 1 |
| 324 | -1.2873 | 1 |
| 325 | -1.2534 | 1 |
| 326 | -1.2461 | 1 |
| 327 | -1.2084 | 1 |
| 328 | -1.2062 | 1 |
| 329 | -1.1957 | 1 |
| 330 | -1.1827 | 1 |
| 331 | -1.1728 | 1 |
| 332 | -1.1436 | 1 |
| 333 | -1.1238 | 1 |
| 334 | -1.1122 | 1 |
| 335 | -1.0972 | 1 |
| 336 | -1.0792 | 1 |
| 337 | -1.0681 | 1 |
| 338 | -1.0529 | 1 |
| 339 | -1.0484 | 1 |
| 340 | -1.0468 | 1 |
| 341 | -0.9997 | 1 |
| 342 | -0.9911 | 1 |
| 343 | -0.9853 | 1 |
| 344 | -0.9749 | 1 |
| 345 | -0.9552 | 1 |
| 346 | -0.9399 | 1 |
| 347 | -0.9339 | 1 |
| 348 | -0.9151 | 1 |
| 349 | -0.899 | 1 |
| 350 | -0.885 | 1 |
| 351 | -0.8731 | 1 |
| 352 | -0.8587 | 1 |
| 353 | -0.8462 | 1 |
| 354 | -0.8305 | 1 |
| 355 | -0.8173 | 1 |
| 356 | -0.8067 | 1 |
| 357 | -0.7906 | 1 |
| 358 | -0.7855 | 1 |
| 359 | -0.7498 | 1 |
| 360 | -0.7366 | 1 |
| 361 | -0.7261 | 1 |
| 362 | -0.717 | 1 |
| 363 | -0.69 | 1 |
| 364 | -0.6638 | 1 |
| 365 | -0.5938 | 1 |
| 366 | -0.4946 | 1 |
| 367 | -0.4906 | 1 |
| 368 | -0.3723 | 1 |
| 369 | -0.3583 | 1 |
| **370** | **-0.2991** | **1** |
| **371** | **1.3489** | **1** |
| **372** | **1.4033** | **1** |
| **373** | **1.6693** | **1** |
| **374** | **5.0603** | **0** |
| 375 | 5.066 | 0 |
| 376 | 5.0973 | 0 |
| 377 | 5.1414 | 0 |
| 378 | 5.1559 | 0 |
| 379 | 5.2102 | 0 |
| 380 | 5.287 | 0 |
| 381 | 5.2925 | 0 |
| 382 | 5.2968 | 0 |
| 383 | 5.3127 | 0 |
| 384 | 5.3163 | 0 |
| 385 | 5.3175 | 0 |
| 386 | 5.3345 | 0 |
| 387 | 5.3432 | 0 |
| 388 | 5.3588 | 0 |
| 389 | 5.3722 | 0 |
| 390 | 5.3752 | 0 |
| 391 | 5.3785 | 0 |
| 392 | 5.3922 | 0 |
| 393 | 5.3947 | 0 |
| 394 | 5.4169 | 0 |
| 395 | 5.4259 | 0 |
| 396 | 5.4308 | 0 |
| 397 | 5.4548 | 0 |
| 398 | 5.4886 | 0 |
| 399 | 5.4946 | 0 |
| 400 | 5.5714 | 0 |
| 401 | 5.6627 | 0 |
| 402 | 5.6853 | 0 |
| 403 | 5.6932 | 0 |
| 404 | 5.8044 | 0 |
| 405 | 5.8278 | 0 |
| 406 | 5.828 | 0 |
| 407 | 5.8605 | 0 |
| 408 | 5.9063 | 0 |
| 409 | 5.9298 | 0 |
| 410 | 5.9466 | 0 |
| 411 | 5.9598 | 0 |
| 412 | 5.9651 | 0 |
| 413 | 6.0167 | 0 |
| 414 | 6.0178 | 0 |
| 415 | 6.0303 | 0 |
| 416 | 6.0676 | 0 |
| 417 | 6.0688 | 0 |
| 418 | 6.0751 | 0 |
| 419 | 6.0914 | 0 |
| 420 | 6.1096 | 0 |
| 421 | 6.1284 | 0 |
| 422 | 6.1523 | 0 |
| 423 | 6.1616 | 0 |
| 424 | 6.1825 | 0 |
| 425 | 6.2043 | 0 |
| 426 | 6.2176 | 0 |
| 427 | 6.2243 | 0 |
| 428 | 6.2415 | 0 |
| 429 | 6.2424 | 0 |
| 430 | 6.2632 | 0 |
| 431 | 6.2717 | 0 |
| 432 | 6.2979 | 0 |
| 433 | 6.3213 | 0 |
| 434 | 6.3364 | 0 |
| 435 | 6.3525 | 0 |
| 436 | 6.4026 | 0 |
| 437 | 6.4091 | 0 |
| 438 | 6.4292 | 0 |
| 439 | 6.4719 | 0 |
| 440 | 6.4937 | 0 |
| 441 | 6.5198 | 0 |
| 442 | 6.5453 | 0 |
| 443 | 6.5677 | 0 |
| 444 | 6.6105 | 0 |
| 445 | 6.6554 | 0 |
| 446 | 6.6975 | 0 |
| 447 | 6.8133 | 0 |
| 448 | 6.9627 | 0 |
| 449 | 6.9917 | 0 |
| 450 | 7.234 | 0 |
| 451 | 7.3302 | 0 |
| 452 | 7.3486 | 0 |
| 453 | 8.2695 | 0 |
| 454 | 8.4089 | 0 |
| 455 | 8.4765 | 0 |
| 456 | 8.6337 | 0 |
| 457 | 8.8781 | 0 |
| 458 | 8.9736 | 0 |
| 459 | 9.0513 | 0 |
| 460 | 9.1577 | 0 |
| 461 | 9.3177 | 0 |
| 462 | 9.3488 | 0 |
| 463 | 9.3695 | 0 |
| 464 | 9.4253 | 0 |
| 465 | 9.4541 | 0 |
| 466 | 9.5618 | 0 |
| 467 | 9.6266 | 0 |
| 468 | 9.6705 | 0 |
| 469 | 9.6911 | 0 |
| 470 | 9.7285 | 0 |
| 471 | 9.7773 | 0 |
| 472 | 9.9869 | 0 |
| 473 | 10.0574 | 0 |
| 474 | 10.1637 | 0 |
| 475 | 10.2157 | 0 |
| 476 | 10.2444 | 0 |
| 477 | 10.2856 | 0 |
| 478 | 10.3795 | 0 |
| 479 | 10.4376 | 0 |
| 480 | 10.4505 | 0 |
| 481 | 10.5033 | 0 |
| 482 | 10.5558 | 0 |
| 483 | 10.5979 | 0 |
| 484 | 10.612 | 0 |
| 485 | 10.707 | 0 |
| 486 | 10.7357 | 0 |
| 487 | 10.7892 | 0 |
| 488 | 10.8742 | 0 |
| 489 | 10.934 | 0 |
| 490 | 10.9577 | 0 |
| 491 | 11.0673 | 0 |
| 492 | 11.1764 | 0 |
| 493 | 11.1957 | 0 |
| 494 | 11.3617 | 0 |
| 495 | 11.4892 | 0 |
| 496 | 11.5365 | 0 |
| 497 | 11.5916 | 0 |
| 498 | 11.6604 | 0 |
| 499 | 11.6793 | 0 |
| 500 | 11.6993 | 0 |
| 501 | 11.7135 | 0 |
| 502 | 11.8102 | 0 |
| 503 | 11.8207 | 0 |
| 504 | 11.8507 | 0 |
| 505 | 11.9501 | 0 |
| 506 | 11.9695 | 0 |
| 507 | 12.1524 | 0 |
| 508 | 12.2422 | 0 |
| 509 | 12.3118 | 0 |
| 510 | 12.3941 | 0 |

**Supplementary Table 13.** DFT calculation result on electron occupation of Ca-doped Li_3_VO_4_ with formation of oxygen vacancy (spin component 2; occupation: 1 = filled, 0 = empty).

| band number | band energies (eV) | occupation |
| --- | --- | --- |
| 1 | -69.3148 | 1 |
| 2 | -69.2516 | 1 |
| 3 | -69.2253 | 1 |
| 4 | -69.0916 | 1 |
| 5 | -69.0909 | 1 |
| 6 | -69.0846 | 1 |
| 7 | -69.08 | 1 |
| 8 | -69.0779 | 1 |
| 9 | -69.0528 | 1 |
| 10 | -69.0521 | 1 |
| 11 | -69.0366 | 1 |
| 12 | -69.0317 | 1 |
| 13 | -68.9856 | 1 |
| 14 | -67.1128 | 1 |
| 15 | -66.7316 | 1 |
| 16 | -66.7294 | 1 |
| 17 | -45.5981 | 1 |
| 18 | -45.5452 | 1 |
| 19 | -45.5445 | 1 |
| 20 | -45.5349 | 1 |
| 21 | -45.261 | 1 |
| 22 | -45.2567 | 1 |
| 23 | -45.2539 | 1 |
| 24 | -45.242 | 1 |
| 25 | -45.2239 | 1 |
| 26 | -45.2 | 1 |
| 27 | -45.196 | 1 |
| 28 | -45.1911 | 1 |
| 29 | -45.1849 | 1 |
| 30 | -45.1825 | 1 |
| 31 | -45.1579 | 1 |
| 32 | -45.1544 | 1 |
| 33 | -45.1317 | 1 |
| 34 | -45.1306 | 1 |
| 35 | -45.0853 | 1 |
| 36 | -45.0831 | 1 |
| 37 | -45.0802 | 1 |
| 38 | -45.0782 | 1 |
| 39 | -45.0696 | 1 |
| 40 | -45.0668 | 1 |
| 41 | -45.0497 | 1 |
| 42 | -45.0446 | 1 |
| 43 | -45.0413 | 1 |
| 44 | -45.0382 | 1 |
| 45 | -45.004 | 1 |
| 46 | -45.0003 | 1 |
| 47 | -44.9804 | 1 |
| 48 | -44.9758 | 1 |
| 49 | -44.9596 | 1 |
| 50 | -44.9574 | 1 |
| 51 | -44.9568 | 1 |
| 52 | -44.9544 | 1 |
| 53 | -44.9234 | 1 |
| 54 | -44.9211 | 1 |
| 55 | -44.8834 | 1 |
| 56 | -44.8807 | 1 |
| 57 | -44.8533 | 1 |
| 58 | -44.8476 | 1 |
| 59 | -44.8395 | 1 |
| 60 | -44.8369 | 1 |
| 61 | -44.7284 | 1 |
| 62 | -44.725 | 1 |
| 63 | -41.5555 | 1 |
| 64 | -41.5394 | 1 |
| 65 | -41.5361 | 1 |
| 66 | -41.4987 | 1 |
| 67 | -41.4816 | 1 |
| 68 | -41.4757 | 1 |
| 69 | -41.4729 | 1 |
| 70 | -41.4429 | 1 |
| 71 | -41.4421 | 1 |
| 72 | -41.3388 | 1 |
| 73 | -41.338 | 1 |
| 74 | -41.3274 | 1 |
| 75 | -41.3267 | 1 |
| 76 | -41.3249 | 1 |
| 77 | -41.3233 | 1 |
| 78 | -41.321 | 1 |
| 79 | -41.3159 | 1 |
| 80 | -41.3152 | 1 |
| 81 | -41.3044 | 1 |
| 82 | -41.3006 | 1 |
| 83 | -41.2962 | 1 |
| 84 | -41.2943 | 1 |
| 85 | -41.2942 | 1 |
| 86 | -41.292 | 1 |
| 87 | -41.2874 | 1 |
| 88 | -41.2867 | 1 |
| 89 | -41.2779 | 1 |
| 90 | -41.2765 | 1 |
| 91 | -41.2759 | 1 |
| 92 | -41.2701 | 1 |
| 93 | -41.2693 | 1 |
| 94 | -41.263 | 1 |
| 95 | -41.2602 | 1 |
| 96 | -41.2571 | 1 |
| 97 | -41.2556 | 1 |
| 98 | -41.2517 | 1 |
| 99 | -41.2184 | 1 |
| 100 | -41.2101 | 1 |
| 101 | -41.2008 | 1 |
| 102 | -40.325 | 1 |
| 103 | -40.0063 | 1 |
| 104 | -39.6465 | 1 |
| 105 | -39.6357 | 1 |
| 106 | -39.633 | 1 |
| 107 | -39.6308 | 1 |
| 108 | -39.0104 | 1 |
| 109 | -38.9859 | 1 |
| 110 | -38.8491 | 1 |
| 111 | -38.1283 | 1 |
| 112 | -38.1218 | 1 |
| 113 | -20.4124 | 1 |
| 114 | -20.2786 | 1 |
| 115 | -20.1509 | 1 |
| 116 | -20.1414 | 1 |
| 117 | -20.1048 | 1 |
| 118 | -20.0258 | 1 |
| 119 | -18.3442 | 1 |
| 120 | -18.2815 | 1 |
| 121 | -18.23 | 1 |
| 122 | -18.1835 | 1 |
| 123 | -18.1009 | 1 |
| 124 | -18.0894 | 1 |
| 125 | -18.0801 | 1 |
| 126 | -18.0734 | 1 |
| 127 | -18.032 | 1 |
| 128 | -18.0157 | 1 |
| 129 | -18.0032 | 1 |
| 130 | -17.9763 | 1 |
| 131 | -17.9435 | 1 |
| 132 | -17.8323 | 1 |
| 133 | -17.8314 | 1 |
| 134 | -17.8244 | 1 |
| 135 | -17.8072 | 1 |
| 136 | -17.7647 | 1 |
| 137 | -17.7315 | 1 |
| 138 | -17.7299 | 1 |
| 139 | -17.7054 | 1 |
| 140 | -17.7015 | 1 |
| 141 | -17.6755 | 1 |
| 142 | -17.6745 | 1 |
| 143 | -17.666 | 1 |
| 144 | -17.6584 | 1 |
| 145 | -17.6482 | 1 |
| 146 | -17.63 | 1 |
| 147 | -17.6279 | 1 |
| 148 | -17.6143 | 1 |
| 149 | -17.6123 | 1 |
| 150 | -17.5918 | 1 |
| 151 | -17.5877 | 1 |
| 152 | -17.586 | 1 |
| 153 | -17.5802 | 1 |
| 154 | -17.5792 | 1 |
| 155 | -17.5704 | 1 |
| 156 | -17.5692 | 1 |
| 157 | -17.5575 | 1 |
| 158 | -17.5469 | 1 |
| 159 | -17.5444 | 1 |
| 160 | -17.5363 | 1 |
| 161 | -17.5341 | 1 |
| 162 | -17.5241 | 1 |
| 163 | -17.5173 | 1 |
| 164 | -17.5165 | 1 |
| 165 | -17.5091 | 1 |
| 166 | -17.4939 | 1 |
| 167 | -17.4872 | 1 |
| 168 | -17.4851 | 1 |
| 169 | -17.4795 | 1 |
| 170 | -17.4522 | 1 |
| 171 | -17.4417 | 1 |
| 172 | -17.1943 | 1 |
| 173 | -17.0868 | 1 |
| 174 | -17.0716 | 1 |
| 175 | -16.9759 | 1 |
| 176 | -16.8178 | 1 |
| 177 | -16.7982 | 1 |
| 178 | -16.7791 | 1 |
| 179 | -16.7702 | 1 |
| 180 | -16.6426 | 1 |
| 181 | -16.5428 | 1 |
| 182 | -4.0739 | 1 |
| 183 | -3.985 | 1 |
| 184 | -3.9652 | 1 |
| 185 | -3.9322 | 1 |
| 186 | -3.9277 | 1 |
| 187 | -3.9225 | 1 |
| 188 | -3.8922 | 1 |
| 189 | -3.8542 | 1 |
| 190 | -3.8459 | 1 |
| 191 | -3.8248 | 1 |
| 192 | -3.8192 | 1 |
| 193 | -3.7929 | 1 |
| 194 | -3.7917 | 1 |
| 195 | -3.764 | 1 |
| 196 | -3.7484 | 1 |
| 197 | -3.7213 | 1 |
| 198 | -3.7097 | 1 |
| 199 | -3.7034 | 1 |
| 200 | -3.67 | 1 |
| 201 | -3.6559 | 1 |
| 202 | -3.6486 | 1 |
| 203 | -3.6249 | 1 |
| 204 | -3.6131 | 1 |
| 205 | -3.6074 | 1 |
| 206 | -3.5781 | 1 |
| 207 | -3.5528 | 1 |
| 208 | -3.5473 | 1 |
| 209 | -3.5373 | 1 |
| 210 | -3.5264 | 1 |
| 211 | -3.5023 | 1 |
| 212 | -3.5013 | 1 |
| 213 | -3.4661 | 1 |
| 214 | -3.4496 | 1 |
| 215 | -3.4215 | 1 |
| 216 | -3.4158 | 1 |
| 217 | -3.4036 | 1 |
| 218 | -3.3876 | 1 |
| 219 | -3.3759 | 1 |
| 220 | -3.362 | 1 |
| 221 | -3.3511 | 1 |
| 222 | -3.3492 | 1 |
| 223 | -3.2774 | 1 |
| 224 | -3.2493 | 1 |
| 225 | -3.2452 | 1 |
| 226 | -3.2169 | 1 |
| 227 | -3.1913 | 1 |
| 228 | -3.1547 | 1 |
| 229 | -3.1379 | 1 |
| 230 | -3.1294 | 1 |
| 231 | -3.1227 | 1 |
| 232 | -3.1209 | 1 |
| 233 | -3.1104 | 1 |
| 234 | -3.0795 | 1 |
| 235 | -3.0762 | 1 |
| 236 | -3.0709 | 1 |
| 237 | -3.0348 | 1 |
| 238 | -3.0153 | 1 |
| 239 | -2.9999 | 1 |
| 240 | -2.9626 | 1 |
| 241 | -2.9598 | 1 |
| 242 | -2.9414 | 1 |
| 243 | -2.9276 | 1 |
| 244 | -2.9111 | 1 |
| 245 | -2.8959 | 1 |
| 246 | -2.8665 | 1 |
| 247 | -2.8662 | 1 |
| 248 | -2.8547 | 1 |
| 249 | -2.8257 | 1 |
| 250 | -2.8072 | 1 |
| 251 | -2.795 | 1 |
| 252 | -2.7765 | 1 |
| 253 | -2.7643 | 1 |
| 254 | -2.7635 | 1 |
| 255 | -2.7365 | 1 |
| 256 | -2.7096 | 1 |
| 257 | -2.7051 | 1 |
| 258 | -2.686 | 1 |
| 259 | -2.67 | 1 |
| 260 | -2.638 | 1 |
| 261 | -2.6077 | 1 |
| 262 | -2.5549 | 1 |
| 263 | -2.5523 | 1 |
| 264 | -2.5277 | 1 |
| 265 | -2.5026 | 1 |
| 266 | -2.4929 | 1 |
| 267 | -2.4267 | 1 |
| 268 | -2.4248 | 1 |
| 269 | -2.4074 | 1 |
| 270 | -2.3808 | 1 |
| 271 | -2.3598 | 1 |
| 272 | -2.3247 | 1 |
| 273 | -2.2997 | 1 |
| 274 | -2.2499 | 1 |
| 275 | -2.2441 | 1 |
| 276 | -2.2204 | 1 |
| 277 | -2.2198 | 1 |
| 278 | -2.1978 | 1 |
| 279 | -2.1722 | 1 |
| 280 | -2.1598 | 1 |
| 281 | -2.1324 | 1 |
| 282 | -2.1315 | 1 |
| 283 | -2.1136 | 1 |
| 284 | -2.1032 | 1 |
| 285 | -2.0959 | 1 |
| 286 | -2.0688 | 1 |
| 287 | -2.0327 | 1 |
| 288 | -2.0199 | 1 |
| 289 | -1.9929 | 1 |
| 290 | -1.9676 | 1 |
| 291 | -1.9618 | 1 |
| 292 | -1.9336 | 1 |
| 293 | -1.9023 | 1 |
| 294 | -1.9018 | 1 |
| 295 | -1.8795 | 1 |
| 296 | -1.8596 | 1 |
| 297 | -1.8575 | 1 |
| 298 | -1.8275 | 1 |
| 299 | -1.8178 | 1 |
| 300 | -1.8015 | 1 |
| 301 | -1.7824 | 1 |
| 302 | -1.7227 | 1 |
| 303 | -1.7023 | 1 |
| 304 | -1.6703 | 1 |
| 305 | -1.6377 | 1 |
| 306 | -1.6364 | 1 |
| 307 | -1.6156 | 1 |
| 308 | -1.593 | 1 |
| 309 | -1.5674 | 1 |
| 310 | -1.5548 | 1 |
| 311 | -1.5305 | 1 |
| 312 | -1.5029 | 1 |
| 313 | -1.4877 | 1 |
| 314 | -1.4872 | 1 |
| 315 | -1.4268 | 1 |
| 316 | -1.4059 | 1 |
| 317 | -1.404 | 1 |
| 318 | -1.378 | 1 |
| 319 | -1.3663 | 1 |
| 320 | -1.3301 | 1 |
| 321 | -1.3087 | 1 |
| 322 | -1.3076 | 1 |
| 323 | -1.2961 | 1 |
| 324 | -1.2766 | 1 |
| 325 | -1.2365 | 1 |
| 326 | -1.2203 | 1 |
| 327 | -1.2048 | 1 |
| 328 | -1.1933 | 1 |
| 329 | -1.1847 | 1 |
| 330 | -1.1801 | 1 |
| 331 | -1.1655 | 1 |
| 332 | -1.1228 | 1 |
| 333 | -1.1201 | 1 |
| 334 | -1.0998 | 1 |
| 335 | -1.0867 | 1 |
| 336 | -1.0622 | 1 |
| 337 | -1.047 | 1 |
| 338 | -1.0383 | 1 |
| 339 | -1.0367 | 1 |
| 340 | -1.0165 | 1 |
| 341 | -0.9975 | 1 |
| 342 | -0.9931 | 1 |
| 343 | -0.9811 | 1 |
| 344 | -0.9709 | 1 |
| 345 | -0.9495 | 1 |
| 346 | -0.9329 | 1 |
| 347 | -0.9322 | 1 |
| 348 | -0.9103 | 1 |
| 349 | -0.8902 | 1 |
| 350 | -0.883 | 1 |
| 351 | -0.8677 | 1 |
| 352 | -0.8587 | 1 |
| 353 | -0.8355 | 1 |
| 354 | -0.8291 | 1 |
| 355 | -0.8166 | 1 |
| 356 | -0.805 | 1 |
| 357 | -0.7895 | 1 |
| 358 | -0.7847 | 1 |
| 359 | -0.7436 | 1 |
| 360 | -0.735 | 1 |
| 361 | -0.7204 | 1 |
| 362 | -0.7136 | 1 |
| 363 | -0.687 | 1 |
| 364 | -0.6627 | 1 |
| 365 | -0.5202 | 1 |
| 366 | -0.4512 | 1 |
| 367 | -0.4105 | 1 |
| 368 | -0.3943 | 1 |
| 369 | -0.3546 | 1 |
| **370** | **-0.3408** | **1** |
| **371** | **1.6393** | **1** |
| **372** | **5.0661** | **0** |
| 373 | 5.068 | 0 |
| 374 | 5.0995 | 0 |
| 375 | 5.1451 | 0 |
| 376 | 5.1558 | 0 |
| 377 | 5.2107 | 0 |
| 378 | 5.2877 | 0 |
| 379 | 5.2939 | 0 |
| 380 | 5.2995 | 0 |
| 381 | 5.3168 | 0 |
| 382 | 5.3177 | 0 |
| 383 | 5.3181 | 0 |
| 384 | 5.3352 | 0 |
| 385 | 5.3434 | 0 |
| 386 | 5.3589 | 0 |
| 387 | 5.373 | 0 |
| 388 | 5.3756 | 0 |
| 389 | 5.3787 | 0 |
| 390 | 5.3929 | 0 |
| 391 | 5.3953 | 0 |
| 392 | 5.4175 | 0 |
| 393 | 5.4269 | 0 |
| 394 | 5.4309 | 0 |
| 395 | 5.4564 | 0 |
| 396 | 5.4896 | 0 |
| 397 | 5.4955 | 0 |
| 398 | 5.5944 | 0 |
| 399 | 5.6722 | 0 |
| 400 | 5.7015 | 0 |
| 401 | 5.784 | 0 |
| 402 | 5.8067 | 0 |
| 403 | 5.835 | 0 |
| 404 | 5.9074 | 0 |
| 405 | 5.9285 | 0 |
| 406 | 5.9476 | 0 |
| 407 | 5.9504 | 0 |
| 408 | 5.9663 | 0 |
| 409 | 6.0174 | 0 |
| 410 | 6.0262 | 0 |
| 411 | 6.0503 | 0 |
| 412 | 6.0602 | 0 |
| 413 | 6.0648 | 0 |
| 414 | 6.0747 | 0 |
| 415 | 6.1091 | 0 |
| 416 | 6.115 | 0 |
| 417 | 6.1293 | 0 |
| 418 | 6.136 | 0 |
| 419 | 6.1645 | 0 |
| 420 | 6.1864 | 0 |
| 421 | 6.2046 | 0 |
| 422 | 6.2197 | 0 |
| 423 | 6.2276 | 0 |
| 424 | 6.2428 | 0 |
| 425 | 6.2532 | 0 |
| 426 | 6.2656 | 0 |
| 427 | 6.2755 | 0 |
| 428 | 6.3019 | 0 |
| 429 | 6.3197 | 0 |
| 430 | 6.3224 | 0 |
| 431 | 6.3262 | 0 |
| 432 | 6.3532 | 0 |
| 433 | 6.4085 | 0 |
| 434 | 6.4679 | 0 |
| 435 | 6.4687 | 0 |
| 436 | 6.5509 | 0 |
| 437 | 6.5728 | 0 |
| 438 | 6.5737 | 0 |
| 439 | 6.6214 | 0 |
| 440 | 6.6466 | 0 |
| 441 | 6.6797 | 0 |
| 442 | 6.9314 | 0 |
| 443 | 6.9333 | 0 |
| 444 | 7.1278 | 0 |
| 445 | 7.2622 | 0 |
| 446 | 7.2777 | 0 |
| 447 | 7.3295 | 0 |
| 448 | 7.3638 | 0 |
| 449 | 7.4441 | 0 |
| 450 | 7.4853 | 0 |
| 451 | 7.786 | 0 |
| 452 | 7.7918 | 0 |
| 453 | 8.3943 | 0 |
| 454 | 8.4364 | 0 |
| 455 | 8.485 | 0 |
| 456 | 8.6612 | 0 |
| 457 | 8.926 | 0 |
| 458 | 8.9804 | 0 |
| 459 | 9.0754 | 0 |
| 460 | 9.1699 | 0 |
| 461 | 9.3571 | 0 |
| 462 | 9.3937 | 0 |
| 463 | 9.411 | 0 |
| 464 | 9.4482 | 0 |
| 465 | 9.4783 | 0 |
| 466 | 9.5671 | 0 |
| 467 | 9.639 | 0 |
| 468 | 9.6842 | 0 |
| 469 | 9.7347 | 0 |
| 470 | 9.7472 | 0 |
| 471 | 9.8783 | 0 |
| 472 | 10.0028 | 0 |
| 473 | 10.0647 | 0 |
| 474 | 10.1742 | 0 |
| 475 | 10.2187 | 0 |
| 476 | 10.2586 | 0 |
| 477 | 10.2991 | 0 |
| 478 | 10.3991 | 0 |
| 479 | 10.4527 | 0 |
| 480 | 10.4954 | 0 |
| 481 | 10.5438 | 0 |
| 482 | 10.5711 | 0 |
| 483 | 10.6031 | 0 |
| 484 | 10.6167 | 0 |
| 485 | 10.716 | 0 |
| 486 | 10.7596 | 0 |
| 487 | 10.7944 | 0 |
| 488 | 10.8786 | 0 |
| 489 | 10.9433 | 0 |
| 490 | 10.9712 | 0 |
| 491 | 11.0874 | 0 |
| 492 | 11.1943 | 0 |
| 493 | 11.2025 | 0 |
| 494 | 11.3727 | 0 |
| 495 | 11.4982 | 0 |
| 496 | 11.5392 | 0 |
| 497 | 11.5979 | 0 |
| 498 | 11.6655 | 0 |
| 499 | 11.6835 | 0 |
| 500 | 11.7066 | 0 |
| 501 | 11.7173 | 0 |
| 502 | 11.8171 | 0 |
| 503 | 11.8309 | 0 |
| 504 | 11.8622 | 0 |
| 505 | 11.9648 | 0 |
| 506 | 11.9808 | 0 |
| 507 | 12.1552 | 0 |
| 508 | 12.26 | 0 |
| 509 | 12.2865 | 0 |
| 510 | 12.3176 | 0 |

**Supplementary Table 14.** Electrochemical performance comparison of 3LCVO-ABR to the previously reported Li_3_VO­_4_-based anode materials.

| materials | current density (mA⸳g^-1^) | specific capacity (mAh⸳g^-1^) | number of cycle | reference |
| --- | --- | --- | --- | --- |
| Li_3_VO_4_ microbox/graphene | 4000 | 223 | 500 | Nano Lett. 2013, 13, 4715−4720 |
| Li_3_VO_4_/graphene nanosheets | 2000 | 163 | 5000 | Chem. Commun., 2015,51, 229-231 |
| Li_3_VO_4_/C | 20 | 245 | 50 | J. Mater. Chem. A, 2015,3, 11253-11260 |
| Li_3_VO_4_/MXene | 2000 | 146 | 1000 | J. Mater. Chem. A, 2019,7, 11250-11256 |
| Li_3_VO_4_/Ag | 150 | 498 | 150 | J. Electrochem. Soc., 2019, 166, A5295 |
| Li_3_VO_4_ cuboid/C | 4000 | 340 | 1000 | ACS Appl. Mater. Interfaces 2016, 8, 1, 680–688 |
| Li_3_VO_4_/C/rGO | 100 | 560 | 100 | ACS Appl. Mater. Interfaces 2018, 10, 23938−23944 |
|  | 2000 | 350 | 1000 |  |
| Fe-doped Li_3_VO_4_ | 100 | 484 | 100 | Electrochim. Acta, 2019, 308, 185e194 |
|  | 200 | 345 | 200 |  |
| Ti-doped Li_3_VO_4_ | 1000 | 328.3 | 500 | J. Phys. Chem. C 2017, 121, 47, 26196–26201 |
| Nb-doped Li_3_VO_4_ | 200 | 418 | 100 | J. Power Sources 378 (2018) 618–627619 |
| Cr-doped Li_3_VO_4_ | 1200 | 147 | 1000 | Funct. Mater. Lett., 2020, 13, 2050005 |
| Mo-doped Li_3_VO_4_ | 2000 | 439 | 200 | *ACS Appl. Mater. Interfaces* 2017, 9, 33, 27688–27696 |
| F-doped Li_3_VO_4_ | 500 | 427 | 1100 | Electrochimica Acta, 2020, 354, 136655 |
| Cu-doped Li_3_VO_­4_ | 1000 | 450 | 150 | ChemElectroChem, 2018, 5, 478– 482 |
| Na-doped Li_3_VO_4_ | 400 | 398.3 | 150 | Solid State Ionics 322 (2018) 30–38 |
| **Ca-doped Li_3_VO_4_** | **100** | **543.1** | **200** | **This work** |
|  | **1000** | **477.1** | **1000** |  |
|  | **4000** | **337.2** | **1000** |  |

**Supplementary Table 15.** Summary of active materials synthesized from ABR process for lithium ion batteries (LIBs) and sodium ion batteries (SIBs).

| materials | application | | capacity  (mAh⸳g^-1^) | current density (mA⸳g^-1^) | cyclability (cycles) | retention (%) |
| --- | --- | --- | --- | --- | --- | --- |
|  | LIBs | SIBs |  |  |  |  |
| LiVO_3_ | cathode |  | 145.6 | 50 | 50 | 70.85 |
| NaVO_3_ orthorhombic |  | cathode | 225.5 | 50 | 50 | 73.77 |
| Li_3_VO_4_ | anode |  | 268.7 | 100 | 200 | 90.27 |
| BiVO_4_ |  | anode | 351.2 | 100 | 100 | 87.62 |
| Na_2_MoO_4_ | anode |  | 305.2 | 30 | 100 | 75.62 |
| Li_2_MoO_4_ | anode |  | 423.2 | 100 | 200 | 69.15 |
| Bi_2_(MoO_4_)_3_ |  | anode | 401.2 | 100 | 200 | 91.25 |
| NiMoO_4_⸳*x*H_2_O | anode |  | 452.3 | 100 | 50 | 74.65 |
| Fe_2_(MoO_4_)_3_ | anode |  | 1983.6 | 100 | 400 | - |
| BiOCl | anode |  | 350.5 | 100 | 20 | 45.51 |
| BiOBr | anode |  | 225.2 | 100 | 20 | 42.53 |
| BiOI | anode |  | 195.6 | 100 | 20 | 45.06 |
| FeFe(CN)_6_ | cathode |  | 131.5 | 100 | 100 | 80.21 |
|  |  | cathode | 145.5 | 100 | 100 | 85.62 |
| Na_2_Zn_3_[Fe(CN)_6_]_2_ |  | cathode | 58.6 | 62 | 1000 | 95.68 |
| Li_2_GeO_3_ | anode |  | 401.1 | 100 | 50 | 84.32 |

**Supplementary References**

1. Wex H, Stratmann F, Topping D, McFiggans G. The Kelvin versus the Raoult term in the Köhler equation. *J Atmos Sci* **65**, 4004-4016 (2008).

2. Topping D, McFiggans G. Tight coupling of particle size, number and composition in atmospheric cloud droplet activation. *Atmos Chem Phys* **12**, 3253 (2012).

3. Thomson GW. The Antoine Equation for Vapor-pressure Data. *Chem Rev* **38**, 1-39 (1946).

4. Grieb M, Brümmer A. Effects of spontaneous condensation in steam-driven screw expanders. *IOP Conference Series: Mater Sci Eng* **232**, 012078 (2017).

5. McDonald JE. Homogeneous Nucleation of Vapor Condensation. I. Thermodynamic Aspects. *Am J Phys* **30**, 870 (1962).

6. Tran Huu H, Nguyen Thi XD, Nguyen Van K, Kim SJ, Vo V. A Facile Synthesis of MoS_2_/g-C_3_N_4_ Composite as an Anode Material with Improved Lithium Storage Capacity. *Materials (Basel)* **12**, (2019).

7. Yu F, Liu Z, Zhou R, Tan D, Wang H, Wang F. Pseudocapacitance contribution in boron-doped graphite sheets for anion storage enables high-performance sodium-ion capacitors. *Mater Horiz* **5**, 529-535 (2018).

8. Cook JB, Kim H-S, Lin TC, Lai C-H, Dunn B, Tolbert SH. Pseudocapacitive Charge Storage in Thick Composite MoS_2_ Nanocrystal-Based Electrodes. *Adv Energy Mater* **7**, (2017).

9. Xiang Y*, et al.* Pseudocapacitive behavior of the Fe_2_O_3_ anode and its contribution to high reversible capacity in lithium ion batteries. *Nanoscale* **10**, 18010-18018 (2018).

10. Chen C*, et al.* Na^+^ intercalation pseudocapacitance in graphene-coupled titanium oxide enabling ultra-fast sodium storage and long-term cycling. *Nat Commun* **6**, 1-8 (2015).

11. Bard AJ, Faulkner LR, Leddy J, Zoski CG. *Electrochemical methods: fundamentals and applications*. wiley New York (1980).

12. Jiang Y, Liu J. Definitions of Pseudocapacitive Materials: A Brief Review. *Energy Environ Mater* **2**, 30-37 (2019).

13. Liu TC, Pell W, Conway B, Roberson S. Behavior of molybdenum nitrides as materials for electrochemical capacitors: comparison with ruthenium oxide. *J Electrochem Soc* **145**, 1882 (1998).
